# Determinants of clinical response to injection laryngoplasty in unilateral vocal fold paralysis: a systematic review and meta-analysis

**DOI:** 10.1097/JS9.0000000000001978

**Published:** 2024-08-14

**Authors:** Alaa Safia, Uday Abd Elhadi, Mohamad Roch, Kenan Kassem, Rabia Safiya, Ahmed Zubeidat, Nader Nofal, Anwar Heib, Shlomo Merchavy, Taiser Bishara

**Affiliations:** a Department of Otolaryngology, Head & Neck Surgery Unit, Rebecca Ziv Medical Center, Safed; b True Doctor, Research Wing, Israel

**Keywords:** injection laryngoplasty, meta-regression, predictors, unilateral, vocal fold paralysis

## Abstract

**Background:**

Unilateral vocal fold paralysis (UVFP) significantly impairs vocal function, affecting patients’ quality of life. Injection laryngoplasty, a primary treatment modality for UVFP, varies in effectiveness based on the material used, injection volume, and procedural nuances. This study aims to systematically analyze how these factors influence treatment outcomes to optimize intervention strategies.

**Materials and methods:**

The authors conducted a comprehensive meta-analysis and meta-regression using data extracted from 82 studies identified through a robust literature search on databases including PubMed, Scopus, and Web of Science, up to 13 February 2024. Eligible studies were single-armed observational or experimental that reported pre and postoperative data on UVFP patients undergoing their first injection laryngoplasty. The primary outcomes analyzed included maximum phonation time, harmonics-to-noise ratio, fundamental frequency, jitter, shimmer, and subjective voice measures such as the Voice Handicap Index and GRBAS scale components.

**Results:**

The meta-analysis revealed significant improvements in maximum phonation time (MPT) and harmonics-to-noise ratio (HNR) post-injection, with variability in outcomes influenced by injection material and procedural techniques. Meta-regression identified the injection volume and the timing of the procedure as significant predictors of MPT and HNR outcomes, respectively. Materials such as polydimethylsiloxane (PDMS) and autologous fat significantly improved MPT and reduced the grade of dysphonia and roughness, respectively. The type of injection material, volume, and approach was crucial in reducing symptoms of voice handicap and enhancing the overall vocal quality.

**Conclusions:**

Injection laryngoplasty significantly improves vocal outcomes in UVFP patients. The choice of injection material, volume, and timing of the intervention play pivotal roles in determining the effectiveness of the procedure. Tailored treatment approaches based on these factors are recommended to enhance therapeutic efficacy and patient satisfaction.

## Introduction

Highlights

- Polydimethylsiloxane (PDMS) and transoral injection techniques significantly enhance phonatory outcomes in unilateral vocal fold paralysis, improving maximum phonation time and harmonics-to-noise ratio.
- Injection volume and timing critically influence treatment efficacy, with smaller volumes and earlier interventions showing superior results in reducing jitter and mean flow rate, enhancing vocal stability and efficiency.
- Meta-regression analysis reveals that the type of injection material and procedural nuances significantly dictate clinical outcomes, underscoring the need for personalized treatment strategies based on individual patient characteristics.

Vocal fold paralysis (VFP) significantly impairs voice, swallowing, and breathing, resulting in substantial functional and psychosocial issues^1^. Unilateral vocal fold paralysis (UVFP), often caused by recurrent laryngeal nerve damage^2^, leads to dysphonia—characterized by hoarseness and breathiness—severely affecting quality-of-life^3^. Management of UVFP is complex, necessitating tailored interventions^4^ including voice therapy and surgical procedures, with injection laryngoplasty being a primary treatment option^5^.

This procedure involves the injection of various biomaterials into the paralyzed vocal fold to improve glottal closure during phonation, thereby enhancing voice quality and vocal efficiency^6^. The choice of injection material—ranging from autologous fat^7^ to synthetic substances like polydimethylsiloxane (PDMS)^8^ and hyaluronic acid^9^—depends on factors such as the desired duration of correction, the individual patient’s anatomy, and pathology, as well as the specific properties of the injectable material.

Despite widespread clinical adoption, the effectiveness of injection laryngoplasty can vary widely, influenced by numerous factors such as the choice of injection material, the specific technique used, and the timing of the intervention^10^. Current literature provides an extensive array of studies on various aspects of injection laryngoplasty^11–20^, yet there remains a significant gap in systematically understanding how these diverse factors collectively influence treatment outcomes. This lack of comprehensive analysis has led to inconsistencies in treatment practices and outcomes, underscoring the need for a robust synthesis of existing research.

This investigation aims to identify the key factors that play a role in determining treatment effects. The lack of a common comparator group hinders the conduct of a network meta-analysis. Thus, we employed subgroup and meta-regression analyses to rigorously examine the impact of several key injection-related characteristics on the effectiveness of treatment.

## Materials and methods

### Design and literature search

The study protocol was previously registered on PROSPERO. This work has been reported in line with the PRISMA, Supplemental Digital Content 1, http://links.lww.com/JS9/D290 ^21^ (Preferred Reporting Items for Systematic Reviews and Meta-Analyses) and AMSTAR, Supplemental Digital Content 2, http://links.lww.com/JS9/D291 ^22^ (Assessing the methodological quality of systematic reviews) Guidelines. We searched PubMed, Scopus, Web of Science, Cochrane Library, and Google Scholar (first 200 citations)^23^ up to 13 February 2024. The search strategy, outlined in SDC, Table 1, Supplemental Digital Content 3, http://links.lww.com/JS9/D292, was adjusted per searched databases. Citations were filtered based on their titles and abstracts. No restrictions were applied regarding the original language of publication. Manual searches included reviewing reference lists and related articles on PubMed^24^.

### Selection strategy

Studies were selected using the PICOS framework^25^. Single-armed and comparative studies of UVFP patients receiving their first injection laryngoplasty were included only if preoperative and postoperative data were provided. Meanwhile, we excluded the following studies: (1) non-original research, (2) non-UVFP patients, (3) repeated injections, (4) lack of information on injection material or timing, (5) multiple injections without data stratification, and (6) overlapping datasets. In studies lacking individual patients’ data, the corresponding/first authors of these studies were contacted by e-mail. In case of no response or authors’ refusal, only the reported data were extracted and later analyzed; otherwise, studies with insufficient data for analysis were excluded.

### Data collection and outcomes

The senior author designed the data collection sheet using Microsoft Excel. The first part covered studies’ (design, follow-up), patients’ (sample, age, sex, UVFP causes, and the mean interval between paralysis and injection), and injections’ characteristics (material, volume, approach). The second part covered patient/study outcomes. Acoustic measures included mean phonation time (MPT), harmonics-to-noise ratio (HNR), fundamental frequency (F0), jitter (%), shimmer (%), normalized noise energy (NNE), mean sound pressure level (SPL) during voicing, amplitude perturbation quotient (APQ), period perturbation quotient (PPQ), and mean flow rate (MFR). The clinical voice assessments included subjective measures like the GRBAS (Grade of dysphonia, Roughness, Breathiness, Asthenia, and Strain) scale, while the objective assessments included glottic gap and voice handicap index (VHI-10 or 30) scales. Other clinical parameters included paralysis resolution or recovery, persistent paralysis, repeated injection, further surgery (thyroplasty), quality-of-life (QoL), and mortality. Non-English studies were translated from Spanish^26^, Chinese^12,27–29^, French^30,31^, Germany^32^, Japanese^33^, and Korean^19,34,35^ before data collection.

### Risk of bias assessment

The risk of bias (RoB) was examined using the MINORS RoB tool for single-armed studies^36^. Five key criteria and seven other parameters were examined. Each point is scored 0 (no reporting), 1 (incomplete reporting), or 2 (satisfactory reporting). The overall score ranges from 0–26, with scores less than 20 indicating high, 20–23 moderate, and 24–26 low risk.

### Statistical analysis

Proper data handling was essential before statistical analysis. We aggregated individual patient data to calculate percentages for binary outcomes and means (standard deviation) for continuous variables, assuming data normality verified by histograms and the Shapiro–Wilk test. Skewed data were expressed as medians (interquartile ranges). Key transformations included converting medians to means using validated formulas^37,38^, standardizing measurement units (e.g. ml/sec for mean flow rate), and adjusting harmonics-to-noise ratio calculations (HNR=1/NHR). We standardized other measurement scales across studies $\left(\mathrm{Shimmer}=10\left(\frac{\mathrm{Shimmer}(\mathrm{dB})}{20}\right)\right)$ , except for VHI, where differences were handled via a standardized mean difference formula ( $\mathrm{smd}=\frac{M1-M2}{{\mathrm{SD}}_{\mathrm{pooled}}}$ )^39^.

Statistical analyses used STATA, following a predefined plan without adjustments. We employed a random-effects model and used the last observation carried forward method to handle data heterogeneity and minimize missing data risks^40^. Heterogeneity was quantified using the I^2^ statistic, with significant heterogeneity defined as I^2^ greater than 40%^41^. Sensitivity analyses tested the robustness of results with Galbraith plots identifying outliers, and publication bias was assessed with funnel plots and asymmetry tests (if >10 studies are reported)^42^.

Subgroup analyses examined variables like follow-up, design, and injection volume, timing, approach, and material. The volume was low (<3 ml) or high (≥3 ml)^43^, and the timing of injection was early (<6) or late (≥6 months)^44^. Meta-regression assessed the impact of study-level covariates, adjusting for multicollinearity, which was evaluated using variance inflation factors (>5 indicates problematic multicollinearity)^45^. Model fit was assessed with the adjusted R-squared (higher values reflect better fit). Variables reported by at least 10 studies were eligible for subgroup and meta-regression (significant heterogeneity is mandatory)^46^.

## Results

### Literature search results

**Figure 1:**
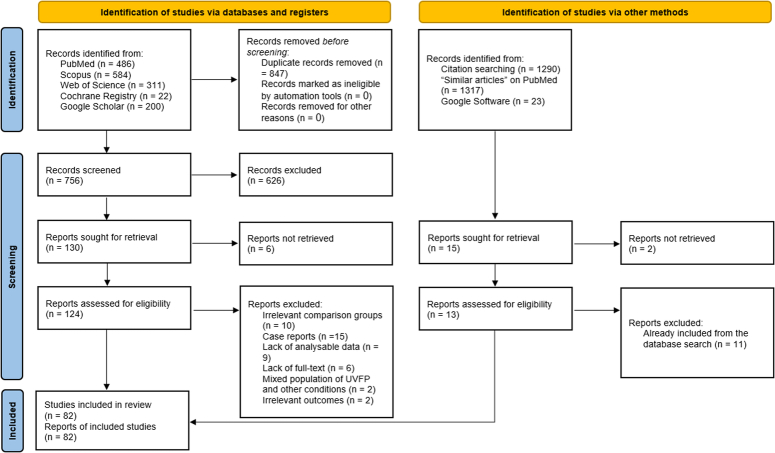
PRISMA flow diagram showing the literature search, manual search, and screening results.

The literature search and screening process yielded 1603 citations, with 847 duplicates identified using EndNote (Fig. 1). After removing duplicates, 756 articles remained, from which 626 were excluded during title/abstract screening. We could not retrieve the full-text for six articles, leaving 124 for full-text review. Of these, 44 were excluded due to reasons such as irrelevant comparison groups, single case reports, lack of analyzable data, abstract-only studies, mixed populations, and irrelevant outcomes. Additionally, a manual search added two relevant articles, resulting in 82 studies being eligible for data synthesis^8,10–16,18–20,26–35,43,44,47–105^.

### Baseline characteristics of examined studies and patients

The characteristics of the included studies, patient demographics, and injection data are detailed in Table 1. The studies primarily involved retrospective cohorts (42.5%), followed by prospective cohorts (32.5%) and case series (20%). The average patient age across 76 studies was 55.05 years. The follow-up period varied, with most studies assessing outcomes at 12 months. Of the 39 studies reporting the timing of injection laryngoplasty, 64.1% noted late injections (beyond 6 months). Autologous fat (30.49%) was the most common injection material, followed by hyaluronic acid (26.83%), calcium hydroxyapatite (9.76%), and collagen (7.32%). The mean injection volume was 1.18 ml, with 94.34% of studies using volumes less than 3 ml. Injections were predominantly administered trans-cervically (58.82%), with the rest performed trans-orally.

**Table 1:** Baseline characteristics of included studies investigating injection laryngoplasty in patients with unilateral vocal fold paralysis.

			Sample			Gender			Injection Laryngoplasty			FU (mo)
Author (YOP)	Country	Design	Pts.	Inj.	Mean Age (yr)	Male	Female	Mean paralysis time (mo)	Material	Volume (mL or cc)	Approach	Mean
Anis (2018)^[48]^	USA	CS	21	20	68	11	10	4	Carboxymethyl cellulose	0.71 ± 0.22 mL	Transoral	4.61
Arviso (2010)^[49]^	USA	RC	42	48	-	28	14	-	Human Dermal Matrix	0.6-3 CC	-	11.73
Bergamini (2010)^[8]^	Italy	LS	15	15	54.67	7	8	<6	Vox implant	2 mL	Transoral	21.67
Chang (1996)^[50]^	China	RC	17	17	-	-	-	-	Autologous Fat	-	-	
Chen (2022)^[105]^	Taiwan	RC	23	23	58.65	9	14	12	Autologous Fat	0.79 ± 0.17	Transcervical	-
Cheng (2009)^[52]^	China	RC	12	12	44.3	8	4	>6	Autologous Fat	2.0-2.5 mL	Transoral	16.6
Cho (2022)^[53]^	Korea	RC	24	24	58.33	13	11	-	Calcium hydroxylapatite	1.5-2.0 mL	Transcervical	11.41
Choi (2020)^[54]^	Korea	RC	76	134	58.4	52	24	-	Collagen	0.5-0.7 mL	Transcervical	-
Chow (2021)^[44]^	Malaysia	CS	29	29	44.69	7	22	6	Hyaluronic acid	0.5-1 mL	Transcervical	-
Chu (1997)^[55]^	Taiwan	RC	20	20	59	14	6	-	Teflon	-	Transoral	-
Cormier (1978)^[56]^	USA	CS	10	10	-	-	-	-	Teflon	0.2-0.6 mL	-	-
Damrose (2010)^[57]^	USA	CS	38	38	60	26	12	-	Collagen	0.5-2 mL	Transoral	-
Elbadan (2017)^[58]^	Egypt	RC	16	16	39.75	11	5	13.13	Autologous Fat	5 cc	Transcervical	-
Elsaeed (2021)^[59]^	Egypt	PC	9	9	41.56	3	6	16.7	Hyaluronic acid	0.75-1.5 mL	Transcervical	-
Fang (2015)^[60]^	Taiwan	RC	34	34	53.5	26	8	4.3	Hyaluronic acid	<1mL	Transcervical	13
Fang (2009)^[61]^	USA	C-S	28	28	50	6	22	26	Autologous Fat	0.5-2 mL (1.2)	Transoral	26
Fang (2010)^[62]^	Taiwan	LS	33	33	45.9	9	24	>6	Autologous Fat	0.5-2 mL	-	-
Francis (2016)^[63]^	USA	RC	115	115	61.1	64	51	9	Human Dermal Matrix	-	-	-
Gandhi (2020)^[64]^	India	RC	60	60	48	22	38	>6	Autologous Fat	0.75-1.5 mL	Transoral	-
Gao (2023)^[65]^	USA	PC	17	22	62	10	7	12	Calcium hydroxylapatite	0.2-0.6 mL	Data Not Stratified	-
Gotxi-Erezuma (2017)^[26]^	Spain	PC	28	28	59.61	8	20	69	Hyaluronic acid	2 mL	-	-
Graboyes (2011)^[11]^	USA	RC	20	20	57.7	15	5	15	Carboxymethyl cellulose	1.1 mL (0.6-1.6)	-	-
Hagemann (2008)^[66]^	Switzerland	CS	21	21	62.33	18	3	43	PDMS	0.5-1 mL	-	-
Hernandez (2016)^[67]^	USA	CS	33	33	63.31	13	20	-	Human Dermal Matrix	-	Data Not Stratified	16.9
Hesaka (1994)^[12]^	Japan	CS	20	20	55	12	8	-	Collagen	0.86 ± 0.32 mL	-	
Hirano (1995)^[68]^	Japan	RC	240	266	59	146	94	-	Silicone	-	Data Not Stratified	93.73
Hirano (1990)^[69]^	Japan	RC	51	53	56.7	30	21	-	Silicone	2mL	Transcervical	-
Hu (2023)^[70]^	China	RC	163	163	34.5	86	77	-	Autologous Fat	-	-	72
Huang (2021)^[27]^	China	RC	22	22	50.66	9	13	-	SVF Gel	1-3 mL	-	-
Hughes (2005)^[13]^	Canada	PC	19	19	71	9	12	6	Calcium hydroxylapatite	0.5-1	-	
Huh (2022)^[71]^	Korea	PC	40	40	61.2	32	8	5.9	Hyaluronic acid	1 ± 0.4	Transcervical	-
Kanazawa (2017)^[72]^	Japan	PC	19	38	60.3	12	7	>3	Human bFGF	50 µg	-	-
Kang (2021)^[73]^	Korea	CS	15	15	67	10	5	-	Hyaluronic acid	0.5-1 mL	Transcervical	-
Karpenko (2003)^[14]^	USA	CS	10	10	-	-	-	-	Human Dermal Matrix	1 mL	Transcervical	-
Khadivi (2016)^[75]^	Iran	PC	20	20	43.4	7	13	7.1	Autologous Fat	1.5-3 mL (1.9 ± 0.3)	-	13.6
Kim (2018a)^[28]^	Korea	PC	50	50	63.1	33	17		Hyaluronic acid	-	Transcervical	-
Kim (2018b)^[76]^	Korea	RC	36	36	64	27	9	12.45	Hyaluronic acid	0.65 mL	Transcervical	-
Koçdor (2014)^[77]^	USA	RC	68	91	59.5	32	36	-	Calcium hydroxylapatite	-	-	13.85
Koelmel (2013)^[29]^	Germany	PC	20	20	-	7	13	-	PDMS	0.6-1 mL	Transoral	-
Laccourreye (1999)^[30]^	France	RC	46	55	55.08	28	18	-	Autologous Fat	-	-	20
Laccourreye (2003)^[31]^	France	RC	80	80	58	53	27	3	Autologous Fat	-	Transoral	
Lee (2017)^[32]^	Korea	RC	43	86	49.67	18	25	-	Hyaluronic acid	-	-	-
Lee (2018)^[78]^	Korea	PC	7	7	69	5	2	-	Calcium hydroxylapatite	-	Transcervical	-
Lee (2011)^[33]^	Korea	CS	16	27	59.94	13	3	-	Calcium hydroxylapatite	0.2-0.65 CC	Transcervical	-
Lee (2010)^[79]^	Korea	PC	34	34	54.6	-	-	-	Hyaluronic acid	0.76 ± 0.2 mL	Data Not Stratified	-
Lim (2012)^[15]^	Korea	RC	29	29	52	14	15	-	Autologous Fat	0.5 mL	Transoral	22.94
Lin (2020)^[10]^	China	PC	73	73	54.71	36	37	-	Autologous Fat	30-40 mL	-	-
Liu (2022)^[51]^	Taiwan	PC	71	71	52.6	47	24	<6	Hyaluronic acid	0.5 mL	Transcervical	-
Lu (2024)^[80]^	Taiwan	PC	22	22	56.2	12	10	87	Hyaluronic acid	90 mL	Transcervical	-
Mattioli (2017)^[82]^	Italy	RC	26	26	60	11	15	<6	PDMS	2 mL	Transoral	73
Milstein (2005)^[16]^	USA	CS	20	20	56.6	7	13	45.1	Human Dermal Matrix	0.76 ± 0.59 mL	Transoral	11.2
Min (2008)^[83]^	Korea	RC	96	132	55	57	39	12.8	Collagen	0.49 ± 0.15	Transcervical	-
Mori (2000)^[34]^	China	RC	325	325	51.3	191	111	-	Silicone	-	-	
Ng (2018)^[84]^	China	PC	30	30	-	-	-	-	Hyaluronic acid	-	Transcervical	-
Oguz (2013)^[85]^	North Cyprus	PC	17	17	41	5	13	>12	Hyaluronic acid	-	-	14
Pagano (2016)^[86]^	Belgium	PC	18	19	61.5	8	10	>9	Autologous Fat	0.75 ± 0.36 mL	Transoral	19.63
Pei (2018)^[88]^	Taiwan	PC	68	68	53.1	-	-	<6	Hyaluronic acid	-	Transcervical	-
Pei (2019)^[87]^	Taiwan	CS	75	75	55.5	47	28	<6	Hyaluronic acid	-	Transcervical	-
Prstacic (2020)^[89]^	Croatia	RC	78	86	52.5	22	56	3	Autologous Fat	-	-	-
Reijonen (2009)^[90]^	Finland	RC	43	43	52.69	4	9	13.2	Autologous Fat	-	-	71.56
Remacle (2006)^[91]^	Belgium	PC	23	23	56	11	12	-	Collagen	0.60-0.2 mL	Transcervical	8
Maccarini (2018)^[81]^	Italy	CS	22	22	60	11	11	>6	Autologous Fat	2-3 CC	Transcervical	-
Rudolf (2012)^[92]^	Germany	PC	19	19	55.2	5	14	>12	Hyaluronic acid	0.9 ± 0.2 mL	-	10.03
Sethi (2018)^[18]^	India	RC	34	34	46	-	-	<6	Hyaluronic acid	-	Transcervical	12 m - 24 m
Shiotani (2009)^[93]^	Japan	CS	56	56	68	-	-	-	Calcium hydroxylapatite	3 cc	Transoral	23.8
Sielska-Badurek (2017)^[94]^	Poland	PC	14	14	57.8	9	5	-	Calcium hydroxylapatite	-	Transoral	-
Sun (2021)^[95]^	China	RC	22	22	49.54	9	13	>6	Autologous Fat	0.5-1.5 mL	Transoral	-
Tanaka (1991)^[19]^	China	RC	55	55	39.1	31	24	-	Autologous Fat	-	-	
Thakar (2021)^[96]^	India	PC	25	25	33.64	16	9	-	Autologous Fat	0.75-1.5 mL	Transoral	-
Tran (2019)^[97]^	Vietnam	LS	41	41	44	13	28	>6	Autologous Fat	0.5-1.5 mL	Transoral	-
Umeno (2007)^[35]^	China	RC	57	57	45	28	29	-	Autologous Fat	-	-	36
Umeno (2022)^[43]^	Japan	RC	73	73	58	31	42	-	Autologous Fat	Low (<3mL); High (>=3mL)	-	
Kara (2018)^[74]^	Turkey	RC	5	5	62.8	4	1		Hyaluronic acid	1 cc	-	-
Wang (2012)^[100]^	Taiwan	PC	20	20	51	6	14	3.85	Hyaluronic acid	1 cc	Transcervical	8.55
Wang (2015a)^[99]^	Taiwan	PC	60	79	52	31	43	-	Hyaluronic acid	1 cc	Transcervical	17.4
Wang (2015b)^[20]^	Taiwan	CS	20	20	64.37	10	10	-	Collagen	0.5-1.5 mL	Transcervical	-
Wong (2016)^[101]^	China	PC	11	11	55	6	5	-	Hyaluronic acid	-	Transcervical	-
Woo (2013)^[102]^	Korea	PC	11	17	53.73	8	3	-	Autologous PPP Gel	1.2 mL	Transcervical	8.36
Yun (2022)^[103]^	Korea	RC	59	59	62.65	31	28	-	Hyaluronic acid	-	Transcervical	10.14
Zuniga (2017)^[104]^	USA	CS	24	24	66.65	12	12	-	Autologous Fat	-	-	
Alaskarov (2024)^[47]^	Turkey	RC	40	40	49.5	18	22	-	Hyaluronic acid	0.7 (0.4-1.1mL)	-	
Umeno (2005)^[98]^	Japan	RC	41	41	57	22	19	>6	Autologous Fat	(0.5-6) mL	-	24

CS, case series; C-S, cross-sectional; FU, follow-up; inj, number of injections; LS, longitudinal study; mo, month; PC, prospective cohort; PDMS, polydimethylsiloxane; pt, number of patients; RC, retrospective cohort; YOP, year of publication; yr, year.

### Risk of bias of included studies

The summary of the risk of bias in each study is presented in Table 2. In summary, 77 studies had a low risk of bias, while the remaining five had a moderate risk of bias with no studies having a high risk of bias.

**Table 2:** Risk of bias of included single-armed studies using the Modified Cowley’s criteria.

	Key Criteria					Other Criteria									
Author (YOP)	P1	P2	P3	P4	P5	P6	P7	P8	P9	P10	P11	P12	P13	Overall Scoring	Overall Bias
Anis (2018)^[48]^	2	2	2	2	2	2	2	2	2	2	2	2	0	24	Low risk
Arviso (2010)^[49]^	2	2	2	2	2	2	2	0	2	2	2	2	1	23	Moderate risk
Bergamini (2010)^[8]^	2	2	2	2	2	2	2	2	2	2	2	2	2	26	Low risk
Chang (1996)^[50]^	2	2	2	2	2	2	2	0	2	2	2	2	0	22	Moderate risk
Chen (2022)^[105]^	2	2	2	2	2	2	2	2	2	2	2	2	2	26	Low risk
Cheng (2009)^[52]^	2	2	2	2	2	2	2	2	2	2	2	2	1	25	Low risk
Cho (2022)^[53]^	2	2	2	2	2	2	2	2	2	2	2	2	0	24	Low risk
Choi (2020)^[54]^	2	2	2	2	2	2	2	2	2	2	2	2	2	26	Low risk
Chow (2021)^[44]^	2	2	2	2	2	2	2	2	2	2	2	2	0	24	Low risk
Chu (1997)^[55]^	2	2	2	2	2	2	2	2	2	2	2	2	1	25	Low risk
Cormier (1978)^[56]^	2	2	0	2	2	2	2	0	2	2	2	2	2	22	Moderate risk
Damrose (2010)^[57]^	2	2	2	2	2	2	2	2	2	2	2	2	1	25	Low risk
Elbadan (2017)^[58]^	2	2	2	2	2	2	2	2	2	2	2	2	2	26	Low risk
Elsaeed (2021)^[59]^	2	2	2	2	2	2	2	2	2	2	2	2	0	24	Low risk
Fang (2015)^[60]^	2	2	2	2	2	2	2	2	2	2	2	2	0	24	Low risk
Fang (2009)^[61]^	2	2	2	2	2	2	2	2	2	2	2	2	1	25	Low risk
Fang (2010)^[62]^	2	2	2	2	2	2	2	2	2	2	2	2	1	25	Low risk
Francis (2016)^[63]^	2	2	2	2	2	2	2	2	2	2	2	2	2	26	Low risk
Gandhi (2020)^[64]^	2	2	2	2	2	2	2	2	2	2	2	2	1	25	Low risk
Gao (2023)^[65]^	2	2	2	2	2	2	2	2	2	2	2	2	0	24	Low risk
Gotxi-Erezuma (2017)^[26]^	2	2	2	2	2	2	2	2	2	2	2	2	0	24	Low risk
Graboyes (2011)^[11]^	2	2	2	2	2	2	2	2	2	2	2	2	1	25	Low risk
Hagemann (2008)^[66]^	2	2	2	2	2	2	2	2	2	2	2	2	2	26	Low risk
Hernandez (2016)^[67]^	2	2	2	2	2	2	2	2	2	2	2	2	1	25	Low risk
Hesaka (1994)^[12]^	2	2	0	2	2	2	2	2	2	2	2	2	2	24	Low risk
Hirano (1995)^[68]^	2	2	2	2	2	2	2	2	2	2	2	2	0	24	Low risk
Hirano (1990)^[69]^	2	2	2	2	2	2	2	2	2	2	2	2	1	25	Low risk
Hu (2023)^[70]^	2	2	2	2	2	2	2	2	2	2	2	2	2	26	Low risk
Huang (2021)^[27]^	2	2	2	2	2	2	2	2	2	2	2	2	1	25	Low risk
Hughes (2005)^[13]^	2	2	2	2	2	2	2	2	2	2	2	2	2	26	Low risk
Huh (2022)^[71]^	2	2	2	2	2	2	2	2	2	2	2	2	0	24	Low risk
Kanazawa (2017)^[72]^	2	2	2	2	2	2	2	2	2	2	2	2	2	26	Low risk
Kang (2021)^[73]^	2	2	2	2	2	2	2	2	2	2	2	2	1	25	Low risk
Karpenko (2003)^[14]^	2	2	2	2	2	2	2	0	2	2	2	2	1	23	Moderate risk
Khadivi (2016)^[75]^	2	2	2	2	2	2	2	2	2	2	2	2	2	26	Low risk
Kim (2018a)^[28]^	2	2	2	2	2	2	2	2	2	2	2	2	1	25	Low risk
Kim (2018b)^[76]^	2	2	2	2	2	2	2	2	2	2	2	2	2	26	Low risk
Koçdor (2014)^[77]^	2	2	2	2	2	2	2	2	2	2	2	2	0	24	Low risk
Koelmel (2013)^[29]^	2	2	2	2	2	2	2	0	2	2	2	2	2	24	Low risk
Laccourreye (1999)^[30]^	2	2	2	2	2	2	2	2	2	2	2	2	1	25	Low risk
Laccourreye (2003)^[31]^	2	2	2	2	2	2	2	2	2	2	2	2	1	25	Low risk
Lee (2017)^[32]^	2	2	2	2	2	2	2	2	2	2	2	2	2	26	Low risk
Lee (2018)^[78]^	2	2	2	2	2	2	2	2	2	2	2	2	0	24	Low risk
Lee (2011)^[33]^	2	2	2	2	2	2	2	2	2	2	2	2	2	26	Low risk
Lee (2010)^[79]^	2	2	2	2	2	2	2	2	2	2	2	2	1	25	Low risk
Lim (2012)^[15]^	2	2	2	2	2	2	2	2	2	2	2	2	1	25	Low risk
Lin (2020)^[10]^	2	2	2	2	2	2	2	2	2	2	2	2	2	26	Low risk
Liu (2022)^[51]^	2	2	2	2	2	2	2	2	2	2	2	2	0	24	Low risk
Lu (2024)^[80]^	2	2	2	2	2	2	2	2	2	2	2	2	0	24	Low risk
Mattioli (2017)^[82]^	2	2	2	2	2	2	2	2	2	2	2	2	2	26	Low risk
Milstein (2005)^[16]^	2	2	2	2	2	2	2	2	2	2	2	2	0	24	Low risk
Min (2008)^[83]^	2	2	2	2	2	2	2	2	2	2	2	2	2	26	Low risk
Mori (2000)^[34]^	2	2	2	2	2	2	2	2	2	2	2	2	1	25	Low risk
Ng (2018)^[84]^	2	2	2	2	2	2	2	0	2	2	2	2	1	23	Moderate risk
Oguz (2013)^[85]^	2	2	2	2	2	2	2	2	2	2	2	2	1	25	Low risk
Pagano (2016)^[86]^	2	2	2	2	2	2	2	2	2	2	2	2	1	25	Low risk
Pei (2018)^[88]^	2	2	2	2	2	2	2	2	2	2	2	2	1	25	Low risk
Pei (2019)^[87]^	2	2	2	2	2	2	2	2	2	2	2	2	1	25	Low risk
Prstacic (2020)^[89]^	2	2	2	2	2	2	2	2	2	2	2	2	2	26	Low risk
Reijonen (2009)^[90]^	2	2	2	2	2	2	2	2	2	2	2	2	0	24	Low risk
Remacle (2006)^[91]^	2	2	2	2	2	2	2	2	2	2	2	2	2	26	Low risk
Maccarini (2018)^[81]^	2	2	2	2	2	2	2	2	2	2	2	2	1	25	Low risk
Rudolf (2012)^[92]^	2	2	2	2	2	2	2	2	2	2	2	2	1	25	Low risk
Sethi (2018)^[18]^	2	2	2	2	2	2	2	2	2	2	2	2	2	26	Low risk
Shiotani (2009)^[93]^	2	2	2	2	2	2	2	2	2	2	2	2	0	24	Low risk
Sielska-Badurek (2017)^[94]^	2	2	2	2	2	2	2	2	2	2	2	2	1	25	Low risk
Sun (2021)^[95]^	2	2	2	2	2	2	2	2	2	2	2	2	1	25	Low risk
Tanaka (1991)^[19]^	2	2	2	2	2	2	2	2	2	2	2	2	1	25	Low risk
Thakar (2021)^[96]^	2	2	2	2	2	2	2	2	2	2	2	2	2	26	Low risk
Tran (2019)^[97]^	2	2	2	2	2	2	2	2	2	2	2	2	2	26	Low risk
Umeno (2007)^[35]^	2	2	2	2	2	2	2	2	2	2	2	2	0	24	Low risk
Umeno (2022)^[43]^	2	2	2	2	2	2	2	2	2	2	2	2	1	25	Low risk
Kara (2018)^[74]^	2	2	2	2	2	2	2	2	2	2	2	2	2	26	Low risk
Wang (2012)^[100]^	2	2	2	2	2	2	2	2	2	2	2	2	0	24	Low risk
Wang (2015a)^[99]^	2	2	2	2	2	2	2	2	2	2	2	2	2	26	Low risk
Wang (2015b)^[20]^	2	2	2	2	2	2	2	2	2	2	2	2	1	25	Low risk
Wong (2016)^[101]^	2	2	2	2	2	2	2	2	2	2	2	2	2	26	Low risk
Woo (2013)^[102]^	2	2	2	2	2	2	2	2	2	2	2	2	0	24	Low risk
Yun (2022)^[103]^	2	2	2	2	2	2	2	2	2	2	2	2	0	24	Low risk
Zuniga (2017)^[104]^	2	2	0	2	2	2	2	2	2	2	2	2	0	22	Low risk
Alaskarov (2024)^[47]^	2	2	2	2	2	2	2	2	2	2	2	2	1	25	Low risk
Umeno (2005)^[98]^	2	2	2	2	2	2	2	2	2	2	2	2	1	25	Low risk

For each point, a score of 2, 1, or 0 was given for satisfactory reporting, partial reporting, and no reporting, respectively. Low risk of bias is given a score of 24-26; moderate risk is give a score of 20-23; high risk is given a score <20. YOP: year of publication.

P1: Method of selection of patients identified and appropriate.

P2: Number of patients deceased or lost to follow-up are either reported or included in appropriate statistical analysis.

P3: Follow-up period, range and mean mentioned.

P4: Interventional strategy specified.

P5: Well-defined criteria for outcomes measurement.

P6: Valid statistical analysis undertaken.

P7: Data mentioned for deceased individuals.

P8: Age range and mean age stated.

P9: Type of lesion stated.

P10: Pre-operative diagnosis and percentages of patients given.

P11: Quantification of outcomes.

P12: Clinical outcomes reported at follow-up.

P13: Independence of investigators (no conflict of interest).

### Acoustic measures

A summary of the pooled acoustic measures is provided in Table 3.

**Table 3:** A summary of the pooled meta-analytical estimate of measured outcomes in patients with unilateral vocal fold paralysis receiving injection laryngoplasty

		Postoperative	Preoperative			
Outcome	K	Effect size	95% CI	I^2^ (*P* value)	Sensitivity analysis	Publication bias
Acoustic measures						
MPT (sec)	54	*n*=1781	*n*=1981			
		4.71	(3.99: 5.43)	93.22% (0.001)	NS change	Shift to the right
HNR	29	*n*=1072	*n*=1091			
		2.8	(1.87: 3.73)	93.89% (0.001)	NS change	Shift to the right
F0 (dB)	23	797	808			
		1.27	(−5.14: 7.68)	49.57% (0.01)	NS change	Shift to the right
Jitter (%)	35	*n*=1120	*n*=1145			
		−2	(−2.40: −1.60)	93.44% (0.001)	NS change	NS
Shimmer (%)	35	*n*=1193	*n*=1207)			
		−2.42	(−3.20: −1.65)	97.07% (0.001)	NS change	Shift to the left
NNE (dB)	8	*n*=508	*n*=508			
		−7.07	(−7.95: −6.20)	20.56% (0.23)	NA	NA
Mean SPL during voicing (dB)	5	*n*=375	*n*=375			
		5.51	(1.77: 9.25)	93.81% (0.001)	NS change	NA
APQ	5	*n*=297	*n*=297			
		−3.09	(−3.47: −2.71)	47.35% (0.11)	NA	NA
PPQ	4	*n*=257	*n*=257			
		−1.78	(−3.07: −0.49)	96.89% (0.001)	Sign change	NA
MFR (ml/sec)	16	*n*=1011	*n*=1211			
		−234.1	(−282.27: −185.93)	97.59% (0.001)	NS change	Shift to the right
Clinical voice assessments						
Dysphonia—GRBAS						
Grade	34	*n*=1203	*n*=1214			
		−1.13	(−1.32: −0.95)	90.72% (0.001)	NS change	NS
Roughness	24	*n*=770	*n*=781			
		−0.64	(−0.83: −0.45)	89.10% (0.001)	NS change	NS
Breathiness	31	*n*=790	*n*=809			
		−1.25	(−1.47: −1.03)	90.69% (0.001)	NS change	NS
Asthenia	20	*n*=696	*n*=697			
		−0.96	(−1.27: −0.66)	97.69% (0.001)	NS change	Shift to the right
Strain	19	*n*=606	*n*=607			
		−0.42	(−0.66: −0.19)	96.26% (0.001)	NS change	Shift to the left
Glottic Gap	7	*n*=178	*n*=179			
		−0.89	(−1.65: −0.13)	95.88% (0.001)	Sign change	NA
VHI	33	*n*=1171	*n*=1729			
		−1.83	(−2.19: −1.48)	99.44% (0.001)	NS change	NS
Clinical outcomes						
Vocal fold paralysis						
Resolved	2	*n*=83				
		57%	(45–69%)	20.99% (0.26)	NA	NA
Full recovery	7	*n*=223				
		38%	(22–54%)	85.21% (0.001)	NS change	NA
Partial recovery	7	*n*=185				
		38%	(15–61%)	94.44% (0.001)	NS change	NA
Persistent paralysis	3	*n*=69				
		13%	(5–20%)	0% (0.78)	NA	NA
Repeated injection	9	*n*=493				
		8%	(4–12%)	63.77% (0.01)	NS change	NA
Need for thyroplasty	13	*n*=833				
		13%	(7–19%)	92.34% (0.001)	NS change	NS
Quality of life	2	*n*=56				
		18.86	(−30.92: 68.63)	97.56% (0.001)	NA	NA
Mortality	6	*n*=224				
		22%	(13–31%)	60.69 (0.02)	NS change	NA

APQ, amplitude perturbation period; F0, fundamental frequency; HNR, harmonics-to-noise ratio; K, number of analyzed studies; K, number of analyzed studies; MFR, mean flow rate; MPT, maximum phonation time; NA, not applicable; n, number of analyzed patients; NNE, normalized noise energy; NS, non-significant; PPQ, period perturbation quotient; SPL, sound pressure level; VHI, voice handicap index.

#### Maximum phonation time (sec)

MPT was improved post-injection [MD=4.71; 95% CI: 3.99: 5.43, I^2^=93.22%]. No change was observed with the sensitivity analysis (SDC, Figure 1, Supplemental Digital Content 3, http://links.lww.com/JS9/D292), and publication bias was observed (SDC, Figure 2, Supplemental Digital Content 3, http://links.lww.com/JS9/D292).

**Figure 2:**
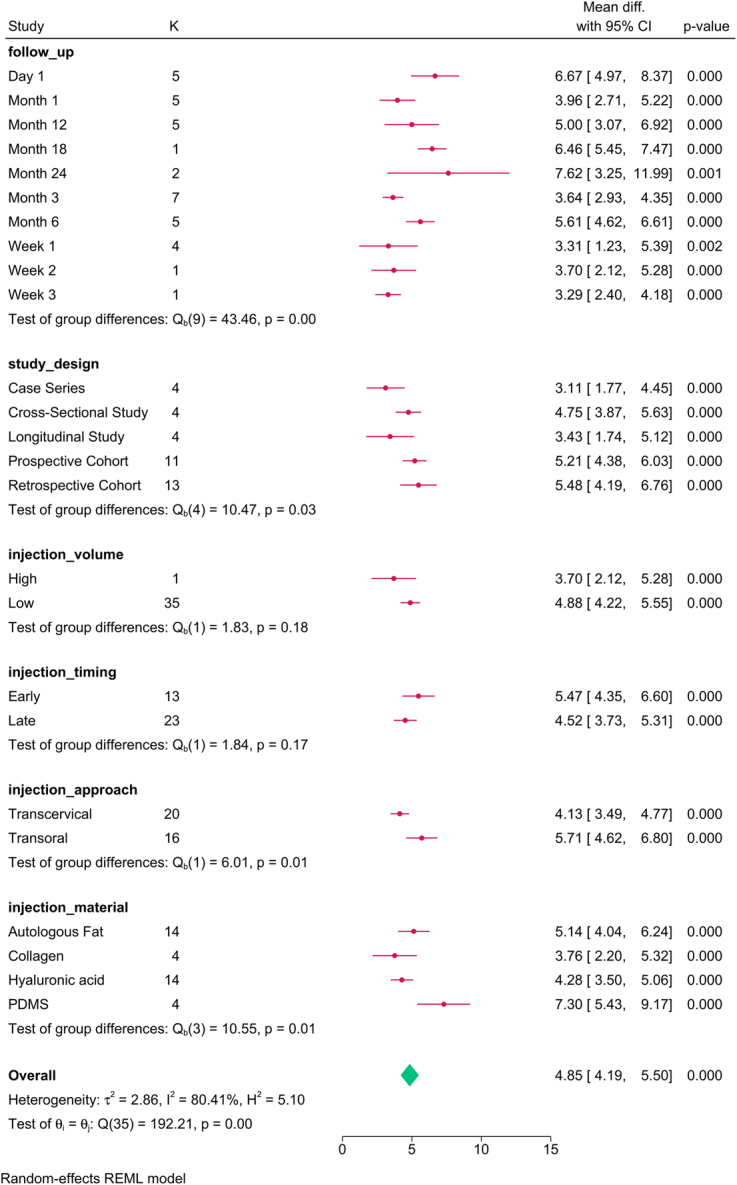
Subgroup analysis of maximum phonation time post-injection based on study design, follow-up, and injection characteristics. K, number of studies per subgroup; PDMS, polydimethylsiloxane.

Study design (*P*=0.03), follow-up (*P*=0.001), injection approach (*P*=0.01), and material (*P*=0.01) modified the reported effect (Fig. 2). MPT significantly increased over time, with the transoral approach and PDMS showing the greatest improvement.

The meta-regression revealed that injection volume (*P*=0.028) and study design accounted for the variation in MPT (Table 4). For each mL increase in the injection volume, the MPT was reduced by 1.358 sec.

**Table 4:** Adjusted meta-regression model of predictors of acoustic measures following injection laryngoplasty

	MPT (sec)		HNR		F0 (dB)		Jitter (%)		Shimmer (%)		MFR (mL/sec)	
	Coefficient	*P*	Coefficient	*P*	Coefficient	*P*	Coefficient	*P*	Coefficient	*P*	Coefficient	*P*
Follow-up (month)	−0.002	0.958	0.115	0.033	−0.056	0.888	−0.015	0.170	−0.625	0.052	−0.527	0.670
Injection volume	−1.358	**0.028**	1.548	0.184	−35.112	0.197	−0.760	**0.000**	−3.137	**0.000**	262.084	**0.001**
Paralysis-to-injection (month)	−1.220	0.193	−2.751	**0.027**	13.856	0.214	1.528	**0.005**	−3.578	**0.005**	−1149.838	**0.001**
Injection approach (transcervical)	−0.632	0.764	−6.852	0.128	129.518	0.238	−0.464	0.600	13.629	**0.000**	−1243.851	**0.000**
Study design [reference: retrospective cohort]												
Case series	−1.390	0.238	−2.750	**0.028**	0.000	omitted	−0.305	0.685	−4.562	**0.001**	0.000	Omitted
Cross-sectional	−2.073	**0.012**	6.270	**0.000**	−0.669	0.947	−1.567	**0.000**	1.384	**0.002**	0.000	Omitted
Longitudinal study	−4.391	**0.000**	6.050	**0.000**	0.000	omitted	−1.568	**0.000**	−7.548	**0.000**	0.000	Omitted
Prospective cohort	0.948	0.252	−2.090	0.082	4.452	0.612	−0.391	0.390	0.077	0.895	—	—
Injection material [reference: autologous fat]												
PPP	0.000	Omitted	0.000	Omitted	0.000	Omitted	0.000	Omitted	0.000	Omitted	0.000	Omitted
Calcium	0.000	Omitted	0.000	Omitted	0.000	Omitted	0.000	Omitted	0.000	Omitted	0.000	Omitted
Carboxymethyl cellulose	0.000	Omitted	0.000	Omitted	0.000	Omitted	0.000	Omitted	0.000	Omitted	0.000	Omitted
bFGF	0.000	Omitted	0.000	Omitted	0.000	Omitted	0.000	Omitted	0.000	Omitted	0.000	Omitted
Dermal Matrix	0.000	Omitted	0.000	Omitted	0.000	Omitted	0.000	Omitted	0.000	Omitted	0.000	Omitted
Hyaluronic acid	−3.583	0.119	6.320	0.227	−154.690	0.194	−1.072	0.314	−18.630	**0.000**	0.000	Omitted
PDMS	2.059	0.129	0.000	Omitted	64.641	**0.046**	0.000	Omitted	0.000	Omitted	0.000	Omitted
Silicone	0.000	Omitted	0.000	Omitted	0.000	Omitted	0.000	Omitted	0.000	Omitted	0.000	Omitted
SVF	0.000	Omitted	0.000	Omitted	0.000	Omitted	0.000	Omitted	0.000	Omitted	0.000	Omitted
Teflon	0.000	Omitted	0.000	Omitted	0.000	Omitted	0.000	Omitted	0.000	Omitted	0.000	Omitted
PAAG	0.000	Omitted	0.000	Omitted	0.000	Omitted	0.000	Omitted	0.000	Omitted	0.000	Omitted
Collagen	−3.369	0.166	5.299	0.326	0.000	Omitted	−1.503	0.149	−17.950	**0.000**	0.000	Omitted
	Adjusted R^2^=71.92		Adjusted R^2^=97.41		Adjusted R^2^=100		Adjusted R^2^=97.72		Adjusted R^2^=95.91		Adjusted R^2^=91.84	
Residual heterogeneity	46.29%		48.04%		0%		26.31%		57.85%		82.36%	

Bold values indicate statistically significant *P*<0.001.

R^2^ reflects the model performance and the ability of included covariates to accurately predict injection laryngoplasty outcomes (a higher score indicates a higher prediction performance; perfect prediction=100).

bFGF, basic fibroblast growth factor; F0, fundamental frequency; HNR, harmonics-to-noise ratio; MFR, mean flow rate; MPT, maximum phonation time; PAAG, polyacrylamide hydrogel; PDMS, polydimethylsiloxane PPP, platelet-poor plasma; SVF, stromal vascular fraction.

#### Harmonics-to-noise ratio

HNR was improved post-injection [MD=2.8; 95% CI: 1.87: 3.73, I^2^=93.89%]. No change was observed with the sensitivity analysis (SDC, Figure 3, Supplemental Digital Content 3, http://links.lww.com/JS9/D292), and publication bias was observed (SDC, Figure 4, Supplemental Digital Content 3, http://links.lww.com/JS9/D292).

**Figure 3:**
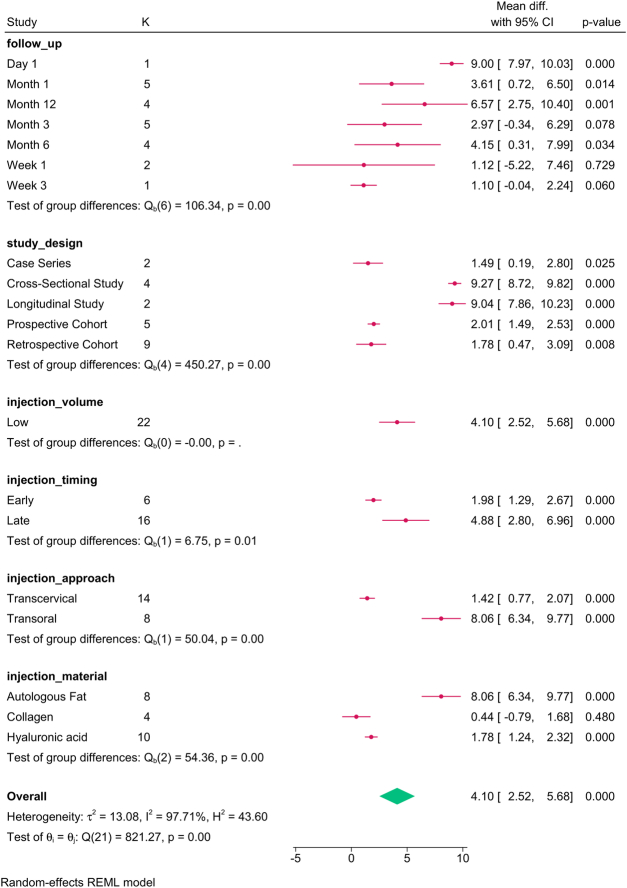
Subgroup analysis of harmonics-to-noise ratio post-injection based on study design, follow-up, and injection characteristics. K, number of studies per subgroup.

The subgroup analysis (Fig. 3) revealed that study design (*P*=0.001), follow-up (*P*=0.001), injection approach (*P*=0.001), timing (*P*=0.01), and material (*P*=0.01) modified the reported effect. HNR increased over time, with the transoral approach, late injections, and autologous fat showing the greatest improvement in HNR.

The meta-regression revealed that the paralysis-to-injection time and study design accounted for the variation in HNR (Table 4).

#### F0 (dB)

F0 showed no improvement post-injection [MD=1.27; 95% CI: −5.14: 7.68, I^2^=49.57%]. No change was observed with the sensitivity analysis (SDC, Figure 5, Supplemental Digital Content 3, http://links.lww.com/JS9/D292), and publication bias was observed (SDC, Figure 6, Supplemental Digital Content 3, http://links.lww.com/JS9/D292).

**Figure 4:**
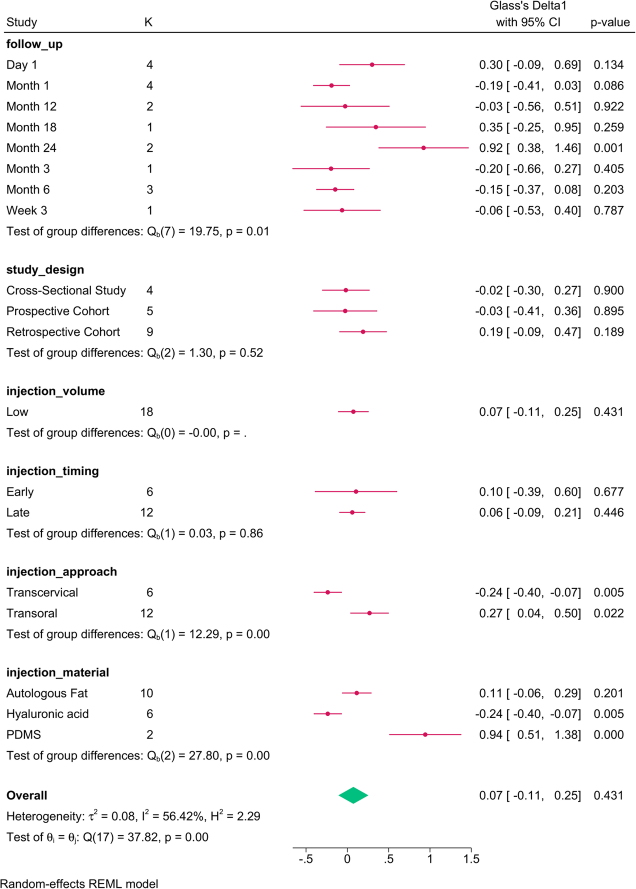
Subgroup analysis of fundamental frequency post-injection based on study design, follow-up, and injection characteristics. K, number of studies per subgroup; PDMS, polydimethylsiloxane.

The subgroup analysis (Fig. 4) revealed that follow-up (*P*=0.01), and injection material (*P*=0.001) modified the reported effect. The change in F0 over time was inconsistent. PDMS increased and hyaluronic acid reduced F0 with autologous fat showing no effect.

The meta-regression revealed that the injection material (*P*=0.046) was the only factor that accounted for the variation in F0 (Table 4). PDMS increased the F0 score by 64.641, compared to autologous fat.

#### Jitter (%)

Jitter was reduced post-injection [MD=−2.00; 95% CI:−2.4:−1.6, I^2^=93.44%]. No change was observed with the sensitivity analysis (SDC, Figure 7, Supplemental Digital Content 3, http://links.lww.com/JS9/D292), and no publication bias was observed (SDC, Figure 8, Supplemental Digital Content 3, http://links.lww.com/JS9/D292).

**Figure 5:**
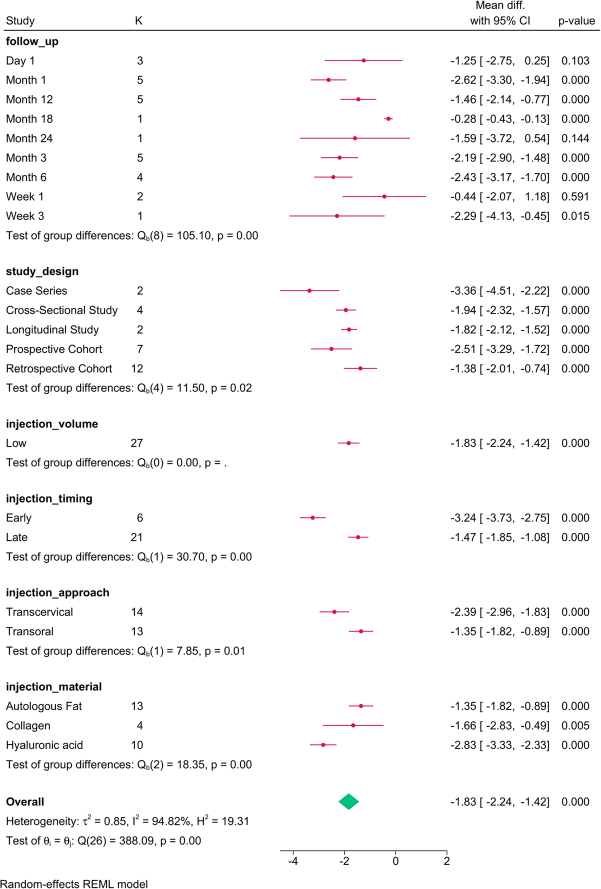
Subgroup analysis of Jitter (%) post-injection based on study design, follow-up, and injection characteristics. K, number of studies per subgroup.

The subgroup analysis (Fig. 5) revealed that follow-up (*P*=0.001) and injection approach (*P*=0.01), timing (*P*=0.01), and material (*P*=0.01) modified the reported effect. Jitter increased over time, with the transcervical approach, early injections, and hyaluronic acid showing the greatest reduction.

The meta-regression revealed that the injection volume (*P*=0.0001), paralysis-injection interval (*P*=0.005), and study design accounted for the variation in Jitter (Table 4). Per each unit increase in the injection volume, the jitter score reduced by 0.76 points, while per each unit increase in the interval between paralysis occurrence to injection, the Jitter score increased by 1.528 points.

#### Shimmer (%)

Shimmer was reduced post-injection [MD=−2.42; 95% CI:−3.2:−1.65, I^2^=97.07%]. No change was observed with the sensitivity analysis (SDC, Figure 9, Supplemental Digital Content 3, http://links.lww.com/JS9/D292), and publication bias was observed (SDC, Figure 10, Supplemental Digital Content 3, http://links.lww.com/JS9/D292).

**Figure 6:**
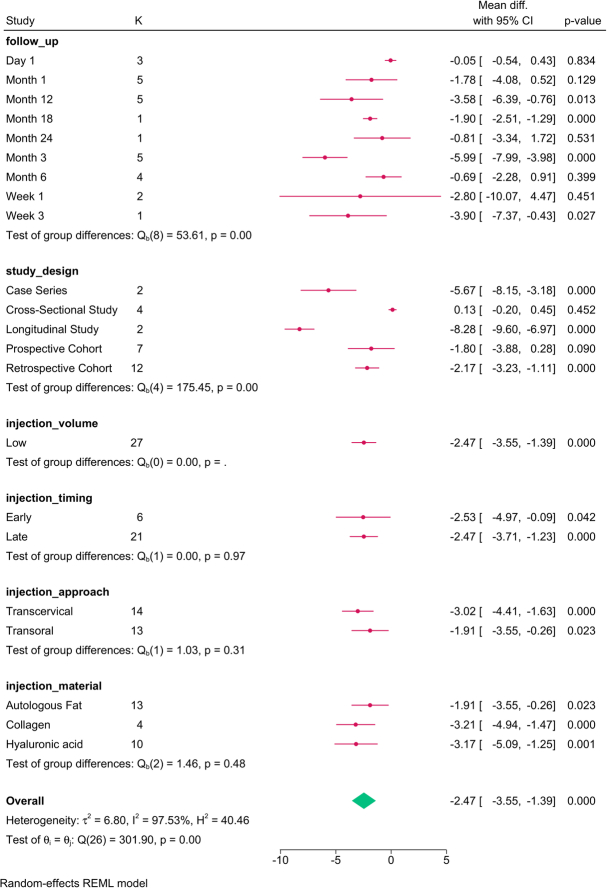
Subgroup analysis of Shimmer (%) post-injection based on study design, follow-up, and injection characteristics. K, number of studies per subgroup.

The subgroup analysis (Fig. 6) revealed that study design (*P*=0.001) and follow-up (*P*=0.001) were the only effect modifiers. The greatest reduction in shimmer was observed at 3 months with a negative trend over time.

The meta-regression revealed that the injection volume (*P*=0.0001), paralysis-injection interval (*P*=0.005), injection approach (*P*=0.0001), and study design accounted for the variability (Table 4). Per each unit increase in the injection volume and per each month increase in the interval between paralysis occurrence to injection, the Jitter score was reduced by 3.137 and 3.578, respectively. The transcervical approach, compared to the transoral approach, increased shimmer by 13.629 points.

#### Mean flow rate (ml/sec)

MFR was reduced post-injection [MD=−234.1; 95% CI:−282.27:−185.93; I^2^=97.59%]. No change was observed with the sensitivity analysis (SDC, Figure 11, Supplemental Digital Content 3, http://links.lww.com/JS9/D292), and publication bias was observed (SDC, Figure 12, Supplemental Digital Content 3, http://links.lww.com/JS9/D292).

**Figure 7:**
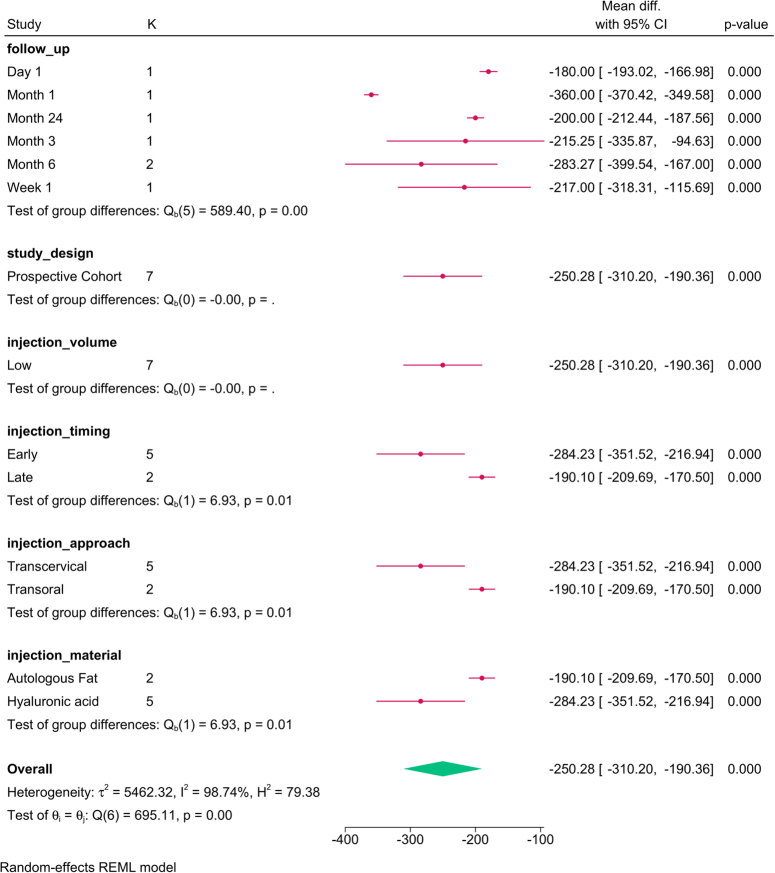
Subgroup analysis of mean flow rate post-injection based on study design, follow-up, and injection characteristics. K, number of studies per subgroup.

The subgroup analysis (Fig. 7) revealed that study design (*P*=0.02), follow-up (*P*=0.001), and injection approach (*P*=0.01), timing (*P*=0.001), and material (*P*=0.01) modified the reported effect. The reduction in MFR was greatest at 1 month with reduced magnitude over time. The transcervical approach, early injections, and hyaluronic acid showed the greatest reduction.

The meta-regression revealed that the injection volume (*P*=0.001), paralysis-injection interval (*P*=0.001), and injection approach (*P*=0.0001) accounted for the variation in MFR (Table 4). Per each unit increase in the injection volume, the MFR increased by 262.084 points. However, per each month’s increase in the interval between paralysis and injection, MFR was reduced by 1149.838 points. The transcervical approach, compared to the transoral approach, reduced MFR score by 1243.851 points.

#### Normalized noise energy (dB)

**Figure 8:**
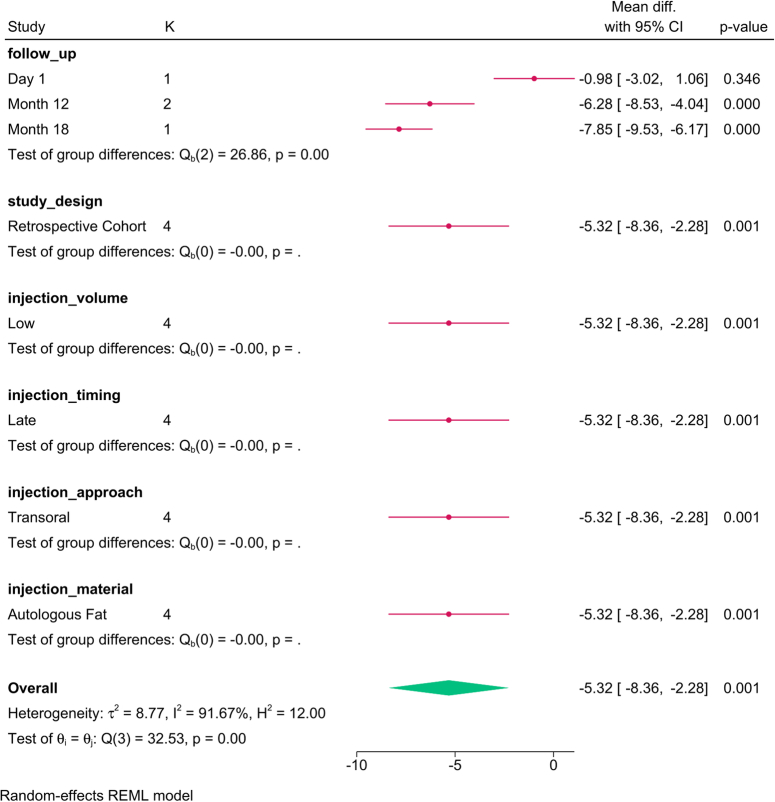
Subgroup analysis of normalized noise energy post-injection based on study design, follow-up, and injection characteristics. K, number of studies per subgroup.

NNE was reduced post-injection [MD=−7.07; 95% CI:−7.95:−6.2, I^2^=20.56%]. The subgroup analysis (Fig. 8) revealed that follow-up (*P*=0.001) was the only effect modifier. The findings were inconclusive as NNE was measured in patients who received late injection, trans-orally, in a low volume of autologous fat.

#### Sound pressure level

SPL was improved post-injection [MD=5.51; 95% CI: 1.77: 9.25, I^2^=93.81%]. No change was observed with the sensitivity analysis (SDC, Figure 13, Supplemental Digital Content 3, http://links.lww.com/JS9/D292).

#### APQ and PPQ

APQ was reduced post-injection [MD=−3.09; 95% CI:−3.47:−2.71, I^2^=47.35%]. Meanwhile, PPQ was reduced post-injection [MD=−1.78; 95% CI:−3.07:−0.49, I^2^=96.89%]. The sensitivity analysis revealed changes in the reported estimate (SDC, Figure 14, Supplemental Digital Content 3, http://links.lww.com/JS9/D292).

### Subjective voice measures (GRBAS)

#### Grade of dysphonia

Dysphonia grade was reduced post-injection [MD=−1.13; 95% CI:−1.32:−0.95, I^2^=90.72%]. No change was observed with the sensitivity analysis (SDC, Figure 15, Supplemental Digital Content 3, http://links.lww.com/JS9/D292), and no publication bias was observed (SDC, Figure 16, Supplemental Digital Content 3, http://links.lww.com/JS9/D292).

**Figure 9:**
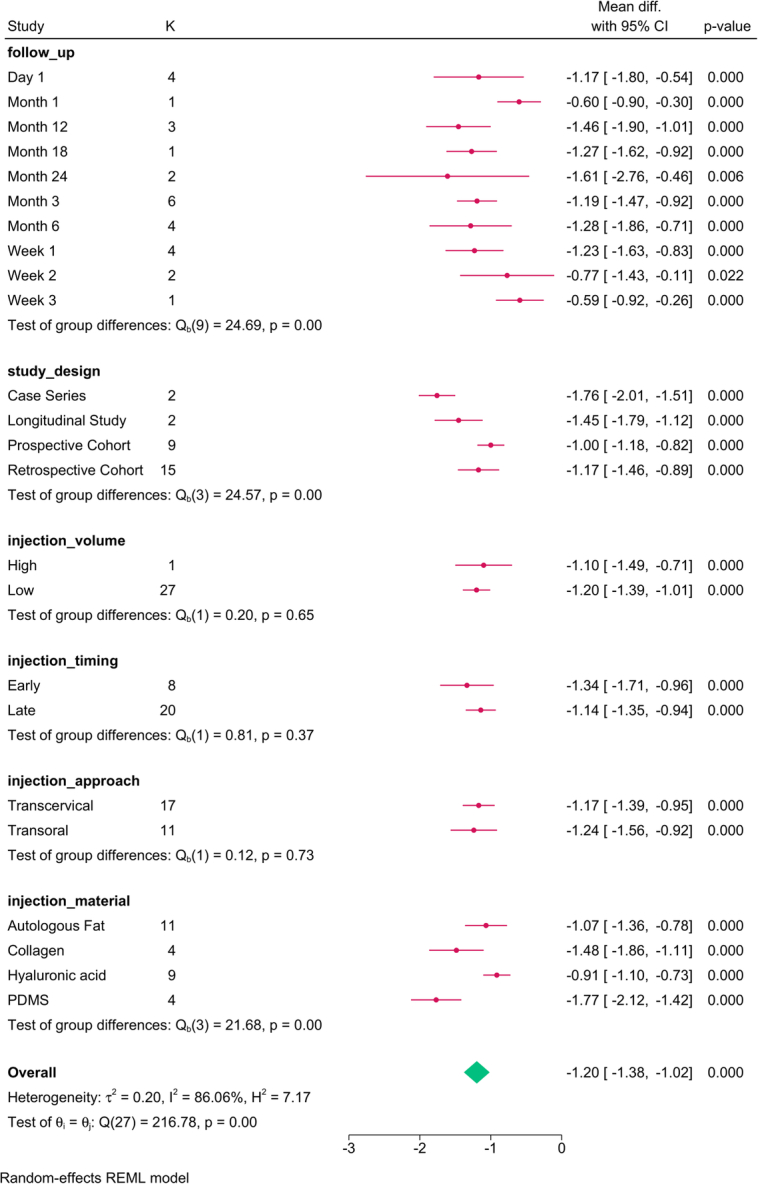
Subgroup analysis of grade of dysphonia post-injection based on study design, follow-up, and injection characteristics. K, number of studies per subgroup; PDMS, polydimethylsiloxane.

The subgroup analysis (Fig. 9) revealed that study design (*P*=0.02), follow-up (*P*=0.001), and injection material (*P*=0.01) modified the reported effect. The improvement in the grade of dysphonia was consistent over time, with PDMS showing the greatest reduction in dysphonia grade.

The meta-regression revealed that follow-up (*P*=0.002), study design (*P*=0.003), and injection material (*P*=0.001) accounted for the variability in treatment effect (Table 5). Per each month’s increase in the follow-up, the grade was reduced by 0.029 points. PDMS and collagen, compared to autologous fat, reduced the grade by 1.202 and 0.633 points, respectively.

**Table 5:** Adjusted meta-regression model of predictors of acoustic measures following injection laryngoplasty

	Grade		Roughness		Breathiness		Asthenia		Strain		VHI	
	Coefficient	*P*	Coefficient	*P*	Coefficient	*P*	Coefficient	*P*	Coefficient	*P*	Coefficient	*P*
Follow-up (month)	−0.029	**0.002**	0.007	0.428	0.007	0.644	−0.008	0.706	−0.004	0.621	−0.318	0.554
Injection volume	−0.105	0.210	0.170	0.289	−0.089	0.460	−0.181	0.729	−0.207	**0.037**	−3.720	0.680
Paralysis-to-injection (month)	−0.208	0.424	0.237	0.466	−0.117	0.729	−0.872	0.349	0.542	0.073	0.525	0.965
Injection approach (transcervical)	−0.180	0.414	0.382	0.145	−0.102	0.770	−0.086	0.912	0.992	**0.000**	9.706	0.415
Study design [reference: retrospective cohort]												
Case series	−0.806	**0.003**	−1.101	**0.002**	−0.907	**0.012**	−0.854	0.420	0.793	**0.000**	17.550	0.196
Cross-sectional	0.000	Omitted	0.000	Omitted	0.000	Omitted	0.000	Omitted	0.000	Omitted	0.000	Omitted
Longitudinal study	−0.294	0.177	0.000	Omitted	0.000	Omitted	0.000	Omitted	0.000	Omitted	−21.090	0.066
Prospective cohort	0.256	0.396	0.256	0.313	−0.687	**0.017**	−1.309	0.064	0.658	**0.011**	1.163	0.916
Injection material [reference: autologous fat]												
PPP	0.000	Omitted	0.000	Omitted	0.000	Omitted	0.000	Omitted	0.000	Omitted	0.000	Omitted
Calcium	0.000	Omitted	0.000	Omitted	0.000	Omitted	0.000	Omitted	0.000	Omitted	0.000	Omitted
Carboxymethyl cellulose	0.000	Omitted	0.000	Omitted	0.000	Omitted	0.000	Omitted	0.000	Omitted	0.000	Omitted
bFGF	0.000	Omitted	0.000	Omitted	0.000	omitted	0.000	Omitted	0.000	Omitted	0.000	Omitted
Dermal Matrix	0.000	Omitted	0.000	Omitted	0.000	Omitted	0.000	Omitted	0.000	Omitted	0.000	Omitted
Hyaluronic acid	−0.008	0.974	−0.389	**0.043**	0.364	0.295	0.196	0.681	−0.318	**0.047**	−6.720	0.557
PDMS	−1.202	**0.001**	−0.491	0.184	−0.499	0.350	−1.363	0.195	1.658	**0.000**	18.897	0.413
Silicone	0.000	Omitted	0.000	Omitted	0.000	Omitted	0.000	Omitted	0.000	Omitted	0.000	Omitted
SVF	0.000	Omitted	0.000	Omitted	0.000	Omitted	0.000	Omitted	0.000	Omitted	0.000	Omitted
Teflon	0.000	Omitted	0.000	Omitted	0.000	Omitted	0.000	Omitted	0.000	Omitted	0.000	Omitted
PAAG	0.000	Omitted	0.000	Omitted	0.000	Omitted	0.000	Omitted	0.000	Omitted	0.000	Omitted
Collagen	−0.633	**0.007**	0.000	Omitted	0.000	Omitted	0.000	Omitted	0.000	Omitted	0.000	Omitted
	Adjusted R2=80.13		Adjusted R2=93.99		Adjusted R2=91.09		Adjusted R2=41.97		Adjusted R2=92.00		Adjusted R2=23.59	
Residual heterogeneity	55.39%		10.78%		71.87%		88.83%		52.27%		98.5	

Bold values indicate statistically significant *P*<0.001.

bFGF, basic fibroblast growth factor; PAAG, polyacrylamide hydrogel; PDMS, polydimethylsiloxane; PPP, platelet-poor plasma; SVF, stromal vascular fraction VHI, voice handicap index.

#### Roughness

Roughness was reduced post-injection [MD=−0.64; 95% CI:−0.83:−0.45, I^2^=89.10%]. No change was observed with the sensitivity analysis (SDC, Figure 17, Supplemental Digital Content 3, http://links.lww.com/JS9/D292), and no publication bias was observed (SDC, Figure 18, Supplemental Digital Content 3, http://links.lww.com/JS9/D292).

**Figure 10:**
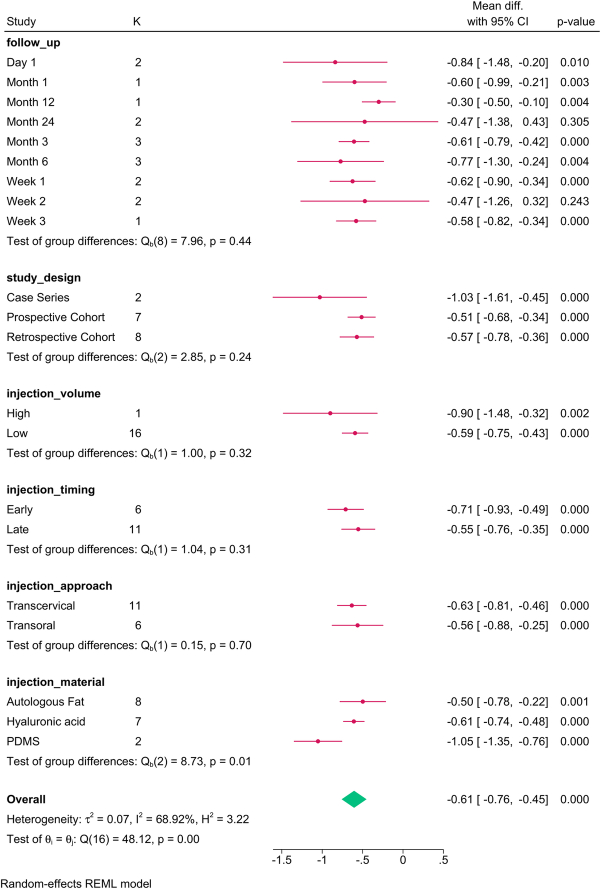
Subgroup analysis of roughness post-injection based on study design, follow-up, and injection characteristics. K, number of studies per subgroup; PDMS, polydimethylsiloxane.

The subgroup analysis (Fig. 10) revealed that injection material (*P*=0.01) was the one factor that modified the reported effect. PDMS resulted in the greatest reduction in roughness followed by hyaluronic acid and autologous fat.

The meta-regression revealed that the study design (*P*=0.002) and injection material (*P*=0.043) accounted for the variation in roughness (Table 5). Hyaluronic acid, compared to autologous fat, reduced roughness by 0.389 points.

#### Breathiness

Breathiness was reduced post-injection [MD=−1.25; 95% CI:−1.47:−1.03, I^2^=90.69%]. No change was observed with the sensitivity analysis (SDC, Figure 19, Supplemental Digital Content 3, http://links.lww.com/JS9/D292), and no publication bias was observed (SDC, Figure 20, Supplemental Digital Content 3, http://links.lww.com/JS9/D292).

**Figure 11:**
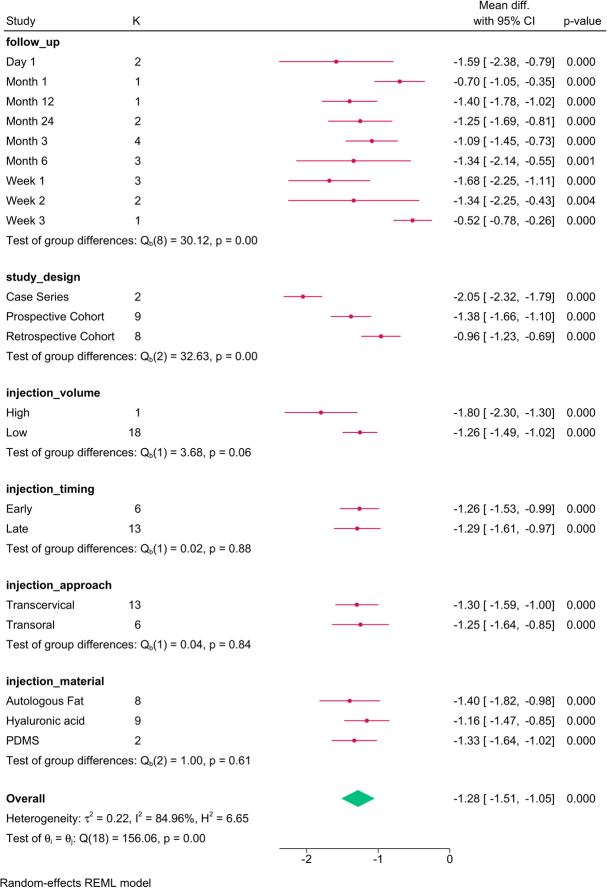
Subgroup analysis of breathiness post-injection based on study design, follow-up, and injection characteristics. K, number of studies per subgroup; PDMS, polydimethylsiloxane.

The subgroup analysis (Fig. 11) revealed that study design (*P*=0.001) and follow-up (*P*=0.001) were the only factors that modified the reported effect. The reduction in breathiness was greatest at week 1, which tended to decrease over time. The meta-regression revealed that the study design (*P*=0.002) accounted for the variation in roughness (Table 5).

#### Asthenia

Asthenia was reduced post-injection [MD=−0.96; 95% CI:−1.27:−0.66, I^2^=97.69%]. No change was observed with the sensitivity analysis (SDC, Figure 21, Supplemental Digital Content 3, http://links.lww.com/JS9/D292), and publication bias was observed (SDC, Figure 22, Supplemental Digital Content 3, http://links.lww.com/JS9/D292).

**Figure 12:**
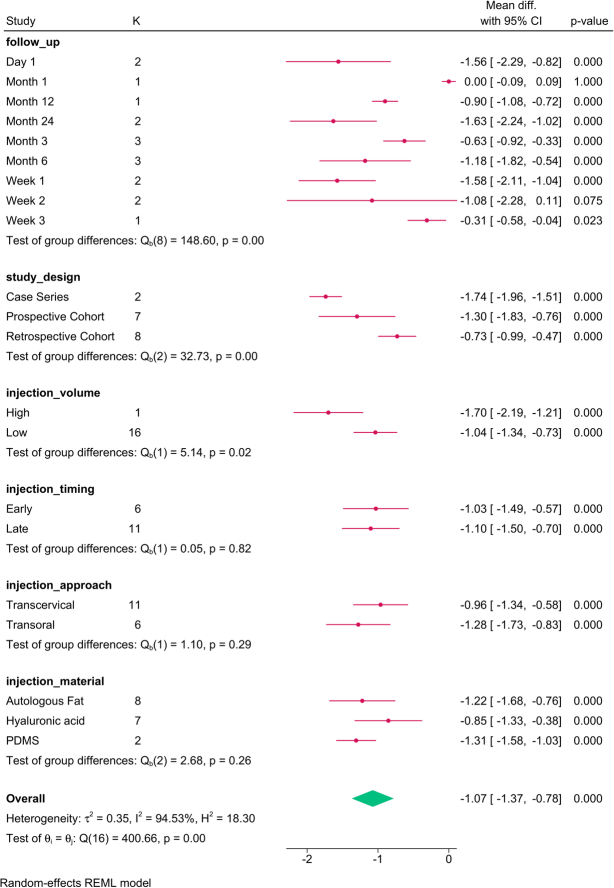
Subgroup analysis of asthenia post-injection based on study design, follow-up, and injection characteristics. K, number of studies per subgroup; PDMS, polydimethylsiloxane.

The subgroup analysis (Fig. 12) revealed that study design (*P*=0.001), follow-up (*P*=0.001), and injection volume (*P*=0.02) modified the reported effect. The improvement in asthenia was minimal during the early follow-up period; however, it was magnified over time. High volumes resulted in the greatest reduction in asthenia.

The meta-regression revealed that none of the analyzed variables accounted for the observed change in the reported effect (Table 5).

#### Strain

Strain was reduced post-injection [MD=−0.42; 95% CI:−0.66:−0.19, I^2^=96.26%]. No change was observed with the sensitivity analysis (SDC, Figure 23, Supplemental Digital Content 3, http://links.lww.com/JS9/D292), and publication bias was observed (SDC, Figure 24, Supplemental Digital Content 3, http://links.lww.com/JS9/D292).

**Figure 13:**
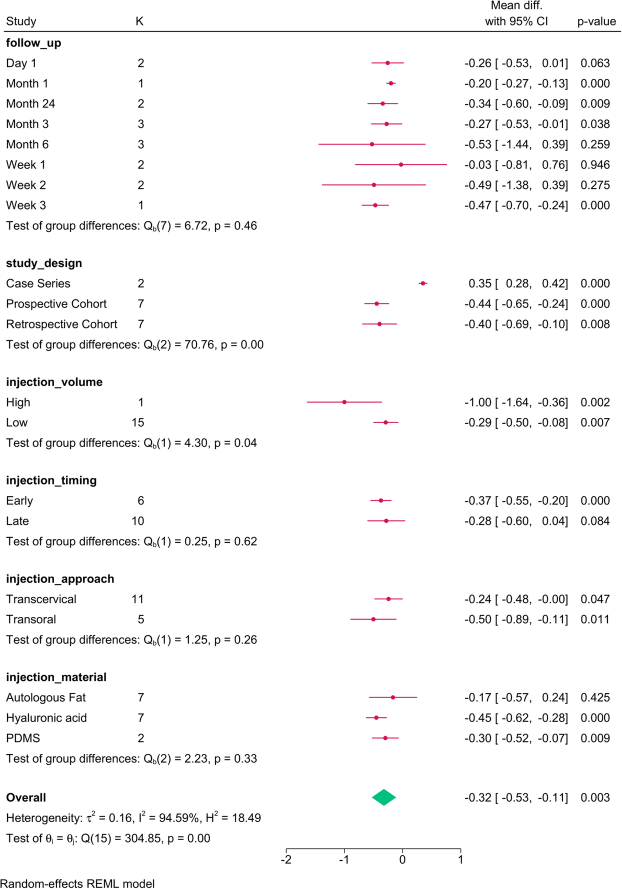
Subgroup analysis of strain post-injection based on study design, follow-up, and injection characteristics. K, number of studies per subgroup; PDMS, polydimethylsiloxane.

The subgroup analysis (Fig. 13) revealed that study design (*P*=0.001) and injection volume (*P*=0.04) modified the reported effect. The strain improvement was consistent over time, with high injection volumes resulting in the greatest benefit.

The meta-regression revealed that injection volume (*P*=0.037), injection approach (*P*=0.0001), study design, and injection material (*P*=0.0.001) accounted for the strain variation (Table 5). Per each mL increase in injected volume, the strain was reduced by 0.207 points. Transcervical injection, compared to trans-orally, increased strain by 0.992 points. Compared to autologous fat, hyaluronic acid reduced strain by 0.318 points, and PDMS increased strain by 1.658 points.

### Objective voice measures

#### Voice handicap index

VHI was reduced post-injection [SMD=−1.83; 95% CI:−2.19:−1.48, I^2^=99.44%]. No change was observed with the sensitivity analysis (SDC, Figure 25, Supplemental Digital Content 3, http://links.lww.com/JS9/D292), and no publication bias was observed (SDC, Figure 26, Supplemental Digital Content 3, http://links.lww.com/JS9/D292).

**Figure 14:**
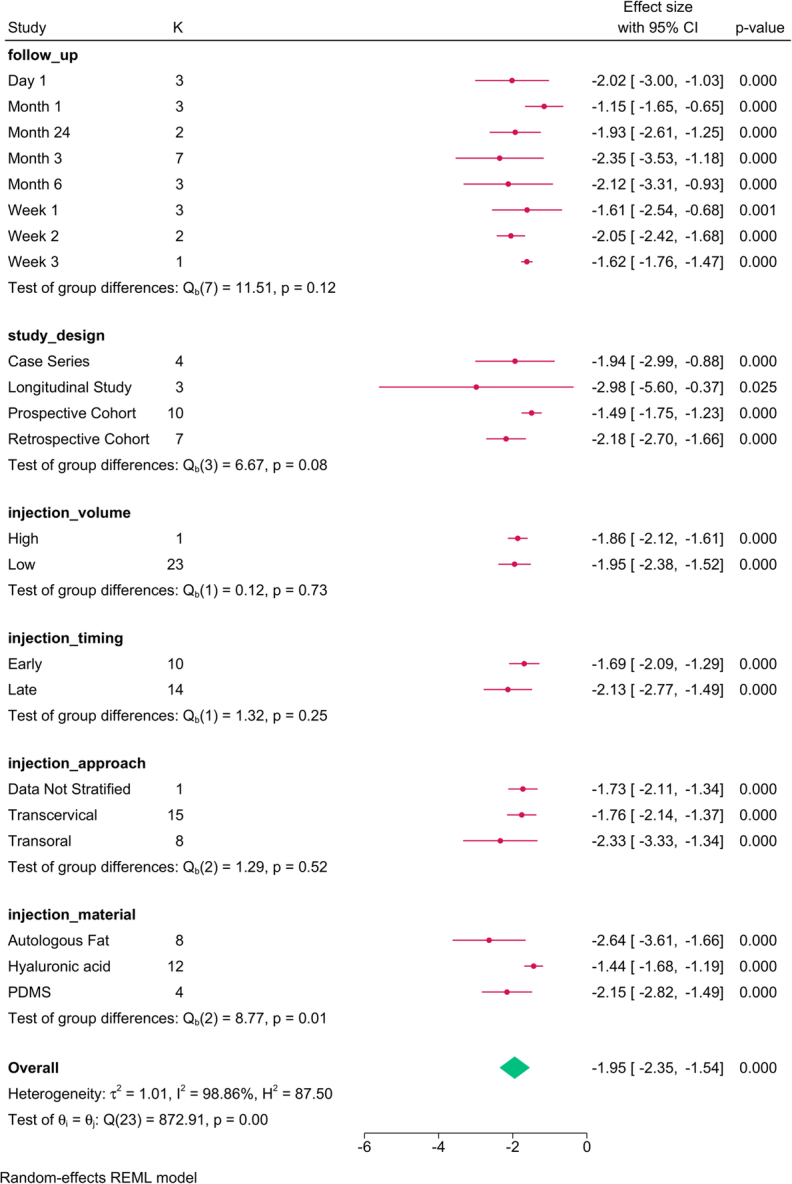
Subgroup analysis of voice handicap index post-injection based on study design, follow-up, and injection characteristics. K, number of studies per subgroup; PDMS, polydimethylsiloxane.

The subgroup analysis (Fig. 14) revealed that the injection material (*P*=0.01) was the only factor that modified the reported effect, with autologous fat showing the greatest benefit. The meta-regression revealed that none of the analyzed covariates accounted for the variation in VHI (Table 5).

#### Glottic gap

**Figure 15:**
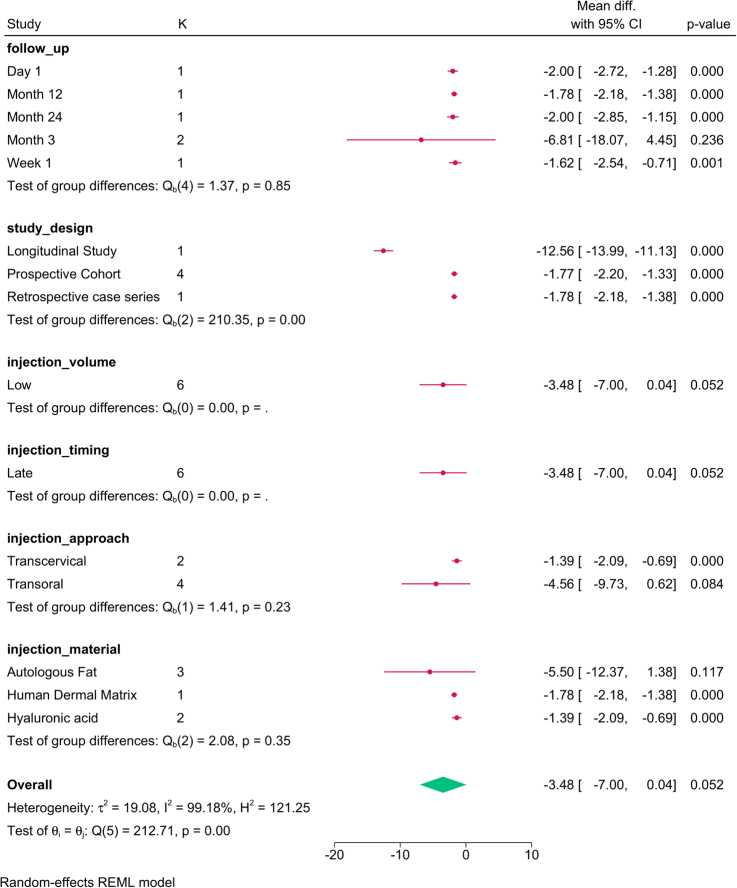
Subgroup analysis of glottic gap post-injection based on study design, follow-up, and injection characteristics. K, number of studies per subgroup.

The glottic gap was improved post-injection [MD=−0.89; 95% CI:−1.65: −0.13, I^2^=95.88%]. The sensitivity analysis showed a change in the reported estimate (SDC, Figure 27, Supplemental Digital Content 3, http://links.lww.com/JS9/D292). The subgroup analysis (Fig. 15) revealed that study design (*P*=0.001) was the only factor that modified the reported effect.

### Clinical Outcomes

#### UVFP resolution and full/partial recovery

Out of 83 patients, 57% [95% CI: 45–69%, I^2^=20.99%] revealed resolution of the paralysis. Of 223 patients, 38% [95% CI: 22–54%, I^2^=85.21%] experienced a full recovery. The sensitivity analysis showed no change in the reported estimate (SDC, Figure 28, Supplemental Digital Content 3, http://links.lww.com/JS9/D292). Of 185 patients, 38% [95% CI: 15–61%, I^2^=94.44%] had partial recovery. The sensitivity analysis showed no change in the reported estimate (SDC, Figure 29, Supplemental Digital Content 3, http://links.lww.com/JS9/D292).

#### Persistent paralysis and repeated injection

Of 69 patients, 13% [95% CI: 5–20%, I^2^=0%] revealed persistent paralysis. Of 493 patients, 8% [95% CI: 4–12%, I^2^=63.77%] required repeated injections. The sensitivity analysis showed no change in the reported estimate (SDC, Figure 30, Supplemental Digital Content 3, http://links.lww.com/JS9/D292).

#### The need for thyroplasty

Of 833 patients, 13% [95% CI: 7–19%, I^2^=92.34%] required thyroplasty medialization. The sensitivity analysis showed no change in the reported estimate (SDC, Figure 31, Supplemental Digital Content 3, http://links.lww.com/JS9/D292), and no publication bias assessment was observed (SDC, Figure 32, Supplemental Digital Content 3, http://links.lww.com/JS9/D292).

#### Quality-of-life and mortality

QoL did not improve post-injection [MD=18.86; 95% CI:−30.92: 68.63, I^2^=97.56%]. Of 224 patients, 22% [95% CI: 13–31%, I^2^=60.69%] died. The sensitivity analysis showed no change in the reported estimate (SDC, Figure 33, Supplemental Digital Content 3, http://links.lww.com/JS9/D292).

## Discussion

The comprehensive synthesis of 82 studies in this meta-analysis provides robust evidence of the efficacy of injection laryngoplasty in improving various vocal and paralytic dysphonia outcomes. Although various systematic reviews have been conducted, none of them analyzed the determinants of clinical response to injection laryngoplasty in UVFP (SDC, Table 2, Supplemental Digital Content 3, http://links.lww.com/JS9/D292).

### Acoustic measures

The significant improvement in MPT, especially notable in studies utilizing the transoral approach and PDMS as the injection material, suggests these factors optimize vocal fold mass and closure, consequently enhancing phonatory duration. These differences can stem from how closely the material can be placed relative to the vocal fold layers^106^. The inverse relationship between MPT and injection volume indicates that smaller volumes may be preferable for achieving optimal vocal fold vibration without excessive stiffness, as supported by our meta-regression^17^. While larger volumes might achieve immediate vocal fold closure, they could also stiffen the vocal folds, reducing their ability to sustain longer phonation.

The findings indicated an inconsistent change in F0 across different time points post-injection, with some materials like PDMS showing a significant increase. This variability may reflect the differential impact of materials on the mass and tension of the vocal folds, which can alter pitch. PDMS specifically was found to increase F0, suggesting that its physical properties might better support the vocal fold structure in a way that enhances vibratory function, leading to higher pitch levels.

The reduction in MFR was most significant early post-injection and decreased over time. The transcervical approach showed the greatest reduction. These findings suggest that early intervention, particularly via the transcervical route, effectively reduces air leakage through the glottis, which could be crucial for patients with significant voice air escape issues^107^. Injection volume and timing post-paralysis were significant predictors, indicating that larger volumes and earlier interventions might more effectively reduce glottic insufficiency, leading to better closure and less airflow during phonation.

The substantial enhancement in HNR post-injection underscores the treatment’s effectiveness in reducing voice signal noise, which is crucial for patients with breathy and weak voices. The greater improvements noted with late injections and autologous fat injections could be attributed to the progressive adaptation and integration of the injection material into the vocal fold tissue, improving its viscoelastic properties^108^. Additionally, late injections leading to better outcomes may indicate the benefits of allowing acute inflammatory responses to subside before intervention.

Reductions in jitter and shimmer post-injection, with significant effects from early injections and specific materials like hyaluronic acid, point towards the procedure’s capacity to stabilize vocal fold vibration, which are essential for a clearer and more consistent voice quality. Injection volume and the interval between paralysis and injection were predictors of jitter improvements. This finding supports the idea that both the quantity of material and the timing of its administration can influence the dynamic properties of the vocal fold vibration. Meanwhile, the greatest reduction in shimmer observed at 3 months post-injection with reductions tapering over time might indicate the initial effectiveness of the treatment, which may diminish as the injected material assimilates or degrades over time^109^. Similar to jitter, the injection volume, and the paralysis-injection interval significantly impacted shimmer, with larger volumes and earlier interventions showing better outcomes. This could relate to the immediate effect of volume in altering vocal fold mass and tension.

### Subjective voice measures

The marked improvements in dysphonia grade and other aspects like roughness and breathiness signify a perceptible enhancement in voice quality from the patient’s perspective. These improvements were markedly sustained over time, suggesting lasting benefits of the injection materials used, especially PDMS and collagen. This reflects a key contribution to the field, as our findings highlight specific factors (such as follow-up period and injection material) that significantly influence patient-reported outcomes. This insight is valuable for clinicians tailoring interventions based on patient needs and expected recovery timelines.

PDMS emerged as a particularly effective material, showing the greatest reduction in both the grade of dysphonia and roughness, with subgroup analyses and meta-regression highlighting its ability to smooth the vibratory pattern of the vocal folds and sustain improvements over time^110^. Interestingly, while PDMS and collagen significantly reduced dysphonia, hyaluronic acid was also effective in diminishing roughness, albeit to a lesser extent than PDMS. The impact on breathiness was notable immediately post-injection but tended to diminish over time. For asthenia, high volumes of injections were linked with significant long-term reductions, though no clear predictors were identified through meta-regression, suggesting a complex interaction of factors at play. Lastly, the strain was consistently improved across different timeframes, particularly with higher injection volumes, indicating that the quantity and delivery method of the injection are key to achieving optimal results in reducing vocal strain.

### Objective voice measures

The significant reduction in VHI reflects the positive impact of injection laryngoplasty on patients’ perceived handicap, thereby enhancing their quality of life. Autologous fat showed the greatest benefit in reducing VHI, indicating its potential superiority in improving perceived voice disability. However, no clear predictors were identified. This suggests a complex interplay of factors beyond the procedural specifics, possibly including patient psychological and social adjustments.

### Clinical outcomes

While a considerable proportion of patients showed resolution or recovery from unilateral vocal fold paralysis (UVFP), the variable results across studies underscore the heterogeneity in recovery potential, which may be influenced by underlying etiological factors, the extent of nerve injury, and timing of intervention. The relatively low re-injection rate and modest requirement for further surgical interventions like thyroplasty indicate the efficacy of injection laryngoplasty as a primary treatment. However, the ongoing need in some patients highlights the importance of tailored treatment plans based on individual response and recovery trajectories.

### Study limitations and future research directions

We observed several limitations. Although we did not restrict our criteria to a certain age group, all of our findings are limited to the adult population. Meanwhile, data on pediatric patients are still lacking. Publication bias in some analyses could overestimate effects. Variations in follow-up lengths could impact the long-term reliability of improvements. The small sample sizes for certain outcomes hindered comprehensive meta-regression analysis, limiting insights into specific injection effects. Clinicians are advised to select the injection material and approach tailored to each patient’s specific characteristics to optimize vocal outcomes and reduce complications. Future research should explore less frequently reported outcomes like injection complications and post-injection effects, which are crucial for a more comprehensive understanding of treatment impacts. Additionally, research should extend to underrepresented populations and various healthcare settings to improve the generalizability of the results. Investigating patient-specific factors influencing recovery will also be crucial in personalizing treatment approaches, thereby maximizing patient outcomes.

## Conclusion

Injection laryngoplasty appears to be a highly effective treatment for improving voice quality and reducing symptoms of vocal fold paralysis. Its role in enhancing both objective and subjective voice measures demonstrates its utility as a critical intervention in the otolaryngology arsenal. However, the treatment’s success is moderated by factors such as injection material, approach, and timing, underscoring the need for personalized treatment planning to optimize outcomes.

## Ethical approval

Not applicable.

## Consent

Not applicable.

## Source of funding

This research received no funding from any institutional or non-institutional organizations.

## Author contribution

Conceptualization: A.S. Data curation: M.R., K.K., R.S., A.Z., N.N., A.H., and T.B. Formal analysis: A.S. and S.M. Funding acquisition: Not applicable. Investigation: A.S. Methodology: U.A.E., M.R., K.K., R.S., A.Z., N.N., A.H., and T.B. Project administration: A.S. Resources: S.M., A.S. Software: S.M. Supervision: S.M. Validation: S.M. and A.S. Writing—original draft: U.A.E., M.R., K.K., and R.S. Writing—review and editing: A.S., S.M., A.Z., N.N., A.H., and T.B. Data analysis and interpretation: A.S. and S.M.

## Conflicts of interest disclosure

The authors declare no competing interests associated with this research.

## Research registration unique identifying number (UIN)

CRD42024521954.

## Guarantor

Alaa Safia.

## Data availability statement

The data used and analyzed in this study can be made available upon reasonable request by contacting the corresponding author.

## Provenance and peer review

Not commissioned, externally peer-reviewed.

## Supplementary Material
