# Supplementary material for: Determinants of clinical response to injection laryngoplasty in unilateral vocal fold paralysis: a systematic review and meta-analysis
Source: Int J Surg. 2024 Aug 14;110(11):7281–310. doi: 10.1097/JS9.0000000000001978 (PMC11573085; doi:10.1097/JS9.0000000000001978)
Supplement: Supplementary file 3 [file js9-110-7281-s003.docx]

**Determinants of Clinical Response to Injection Laryngoplasty in Unilateral Vocal Fold Paralysis: A Systematic Review and Meta-Analysis**

**SDC, Table 1.** The detailed search strategy employed in the literature search

| Database | No | Search Query | Results |
| --- | --- | --- | --- |
| PubMed | #1 | Inject*[tiab] OR “injection laryngoplasty”[tiab] OR “injection medialization laryngoplasty”[tiab] OR “hyaluronic acid”[tiab] OR hyaluronate[tiab] OR Restylane[tiab] OR collagen[tiab] OR Cymetra[tiab] OR Zyplast[tiab] OR Zyderm[tiab] OR “Radiesse Voice Gel”[tiab] OR Gelfoam[tiab] OR “calcium hydroxyapatite”[tiab] OR “calcium hydroxylapatite”[tiab] OR fat[tiab] OR fascia[tiab] OR polydimethylsiloxane[tiab] OR Bioplastique[tiab] OR “basic fibroblast growth factor”[tiab] OR “fat injection”[tiab] OR teflon[tiab] OR “cartilage injection” OR “calcium phosphate cement”[tiab] OR “micronized acellular dermis”[tiab] OR “fibrin gel”[tiab] OR hydroxyapatite[tiab] OR juvederm[tiab] OR “plasma gel”[tiab] OR lipoinjection[tiab] OR "Injections"[Mesh] | 1623498 |
|  | #2 | “Vocal cord paralysis”[tiab] OR “vocal cord palsy”[tiab] OR “vocal cord paralyses”[tiab] OR “vocal cord palsies”[tiab] OR “vocal cord paresis”[tiab] OR “vocal fold paralysis”[tiab] OR “vocal fold palsy”[tiab] OR “vocal fold paralyses”[tiab] OR “vocal fold palsies”[tiab] OR “vocal fold paresis”[tiab] OR "Vocal Cord Paralysis"[Mesh] | 9090 |
|  | #3 | Unilateral[tiab] | 156208 |
|  | #4 | #1 AND #2 AND #3 | 486 |
| Scopus | #1 | TITLE-ABS-KEY(Inject*) OR TITLE-ABS-KEY(“injection laryngoplasty”) OR TITLE-ABS-KEY(“injection medialization laryngoplasty”) OR TITLE-ABS-KEY(“hyaluronic acid”) OR TITLE-ABS-KEY(hyaluronate) OR TITLE-ABS-KEY(Restylane) OR TITLE-ABS-KEY(collagen) OR TITLE-ABS-KEY(Cymetra) OR TITLE-ABS-KEY(Zyplast) OR TITLE-ABS-KEY(Zyderm) OR TITLE-ABS-KEY(“Radiesse Voice Gel”) OR TITLE-ABS-KEY(Gelfoam) OR TITLE-ABS-KEY(“calcium hydroxyapatite”) OR TITLE-ABS-KEY(“calcium hydroxylapatite”) OR TITLE-ABS-KEY(fat) OR TITLE-ABS-KEY(fascia) OR TITLE-ABS-KEY(polydimethylsiloxane) OR TITLE-ABS-KEY(Bioplastique) OR TITLE-ABS-KEY(“basic fibroblast growth factor”) OR TITLE-ABS-KEY(“fat injection”) OR TITLE-ABS-KEY(teflon) OR TITLE-ABS-KEY(“cartilage injection”) OR TITLE-ABS-KEY(“calcium phosphate cement”) OR TITLE-ABS-KEY(“micronized acellular dermis”) OR TITLE-ABS-KEY(“fibrin gel”) OR TITLE-ABS-KEY(hydroxyapatite) OR TITLE-ABS-KEY(juvederm) OR TITLE-ABS-KEY(“plasma gel”) OR TITLE-ABS-KEY(lipoinjection) | 2868711 |
|  | #2 | TITLE-ABS-KEY(“Vocal cord paralysis”) OR TITLE-ABS-KEY(“vocal cord palsy”) OR TITLE-ABS-KEY(“vocal cord paralyses”) OR TITLE-ABS-KEY(“vocal cord palsies”) OR TITLE-ABS-KEY(“vocal cord paresis”) OR TITLE-ABS-KEY(“vocal fold paralysis”) OR TITLE-ABS-KEY(“vocal fold palsy”) OR TITLE-ABS-KEY(“vocal fold paralyses”) OR TITLE-ABS-KEY(“vocal fold palsies”) OR TITLE-ABS-KEY(“vocal fold paresis”) | 12638 |
|  | #3 | TITLE-ABS-KEY(Unilateral) | 200148 |
|  | #4 | #1 AND #2 AND #3 | 584 |
| Web of Science | #1 | AB=Inject* OR AB=“injection laryngoplasty” OR AB=“injection medialization laryngoplasty” OR AB=“hyaluronic acid” OR AB=hyaluronate OR AB=Restylane OR AB=collagen OR AB=Cymetra OR AB=Zyplast OR AB=Zyderm OR AB=“Radiesse Voice Gel” OR AB=Gelfoam OR AB=“calcium hydroxyapatite” OR AB=“calcium hydroxylapatite” OR AB=fat OR AB=fascia OR AB=polydimethylsiloxane OR AB=Bioplastique OR AB=“basic fibroblast growth factor” OR AB=“fat injection” OR AB=teflon OR AB=“cartilage injection” OR AB=“calcium phosphate cement” OR AB=“micronized acellular dermis” OR AB=“fibrin gel” OR AB=hydroxyapatite OR AB=juvederm OR AB=“plasma gel” OR AB=lipoinjection | 1574473 |
|  | #2 | AB=“Vocal cord paralysis” OR AB=“vocal cord palsy” OR AB=“vocal cord paralyses” OR AB=“vocal cord palsies” OR AB=“vocal cord paresis” OR AB=“vocal fold paralysis” OR AB=“vocal fold palsy” OR AB=“vocal fold paralyses” OR AB=“vocal fold palsies” OR AB=“vocal fold paresis” | 3503 |
|  | #3 | AB=Unilateral | 124929 |
|  | #4 | #1 AND #2 AND #3 | 311 |
| CENTRAL | #1 | Inject* OR “injection laryngoplasty” OR “injection medialization laryngoplasty” OR “hyaluronic acid” OR hyaluronate OR Restylane OR collagen OR Cymetra OR Zyplast OR Zyderm OR “Radiesse Voice Gel” OR Gelfoam OR “calcium hydroxyapatite” OR “calcium hydroxylapatite” OR fat OR fascia OR polydimethylsiloxane OR Bioplastique OR “basic fibroblast growth factor” OR “fat injection” OR teflon OR “cartilage injection” OR “calcium phosphate cement” OR “micronized acellular dermis” OR “fibrin gel” OR hydroxyapatite OR juvederm OR “plasma gel” OR lipoinjection | 187981 |
|  | #2 | “Vocal cord paralysis” OR “vocal cord palsy” OR “vocal cord paralyses” OR “vocal cord palsies” OR “vocal cord paresis” OR “vocal fold paralysis” OR “vocal fold palsy” OR “vocal fold paralyses” OR “vocal fold palsies” OR “vocal fold paresis” | 304 |
|  | #3 | Unilateral | 16463 |
|  | #4 | #1 AND #2 AND #3 | 22 |
| Google Scholar | With all of the words | unilateral | - |
|  | With the exact phrase | Vocal cord paralysis | - |
|  | With at least one of the words | Injection injectable inject “injection laryngoplasty” “injection medialization laryngoplasty” “hyaluronic acid” hyaluronate Restylane collagen Cymetra Zyplast OR Zyderm “Radiesse Voice Gel” Gelfoam “calcium hydroxyapatite” “calcium hydroxylapatite” fat fascia polydimethylsiloxane Bioplastique “basic fibroblast growth factor” “fat injection” teflon “cartilage injection” “calcium phosphate cement” “micronized acellular dermis” “fibrin gel” hydroxyapatite juvederm “plasma gel” lipoinjection | - |
|  | Total | - | 200 |

**SDC, Table 2.** A summary of various systematic reviews conducted on injection laryngoplasty in UVFP

| Author (YOP) | Material | Sample | Findings | Measured Outcomes | Notes |
| --- | --- | --- | --- | --- | --- |
| Wang (2020) | Hyaluronic Acid | 14 studies | Improved | Glottal Closure | The authors analyzed outcomes based on the follow-up period alone without doing meta-regression analyses to identify the determinants of analyzed outcomes |
|  | | | Prolonged | MPT |  |
|  |  |  | Improved | QoL |  |
|  |  |  | Increased in all domains | GRBAS |  |
|  |  |  | 6.18 - 7.33% | Normalized Glottal Gap (%) |  |
| Siu (2015) | Not clearly described | 3 studies | Higher score | VHI | The authors compared various interventions for UVFP without doing any meta-analyses |
|  | | | No difference | Subjective measures (Jitter, Shimmer, and HNR) |  |
| Švejdová (2022) | Hyaluronic Acid | 13 studies | Higher score | VHI | The authors investigated the effect of injection amount on measured outcomes. However, other factors like injection timing were not examined |
|  | | | Large-particle HA gels had longer durability than small-particle HA | Acoustic and aerodynamic measures |  |
|  |  |  | Low rate | Complications |  |
| Vila (2017) | Not clearly described | 4 studies | Early injections had a lower relative risk of thyroplasty compared to late or no injections | Thyroplasty rate | Heterogeneity was high and the authors did not perform any analyses to determine the sources of heterogeneity |
| Liao (2022) | Fat, PAAG, hyaluronic acid, and PMMA | 5 studies | Improved | MPT | The authors selectively chose only 2 outcomes to report without examining other measures |
|  | | | Improved | Jitter |  |
| Haddad (2021) | Lipoinjection | 49 studies | Improved | MPT | The authors intended to carry out a subgroup analysis based on timing but they could not due to inconsistent reporting |
|  | | | Improved | Jitter |  |
|  |  |  | Improvement was only observed for grade, breathiness, and asthenia | GRBAS |  |
|  |  |  | Improved | VHI |  |

YOP: year of publication; QoL: quality of life; MPT: maximum phonation time; VHI: voice handicap index; GRBAS: grade, roughness, breathiness, asthenia, and strain; HNR: harmonics-to-noise ratio; PAAG: polyacrylamide hydrogel; PMMA: polymethylmethacrylate; UVFP: unilateral vocal fold paralysis.


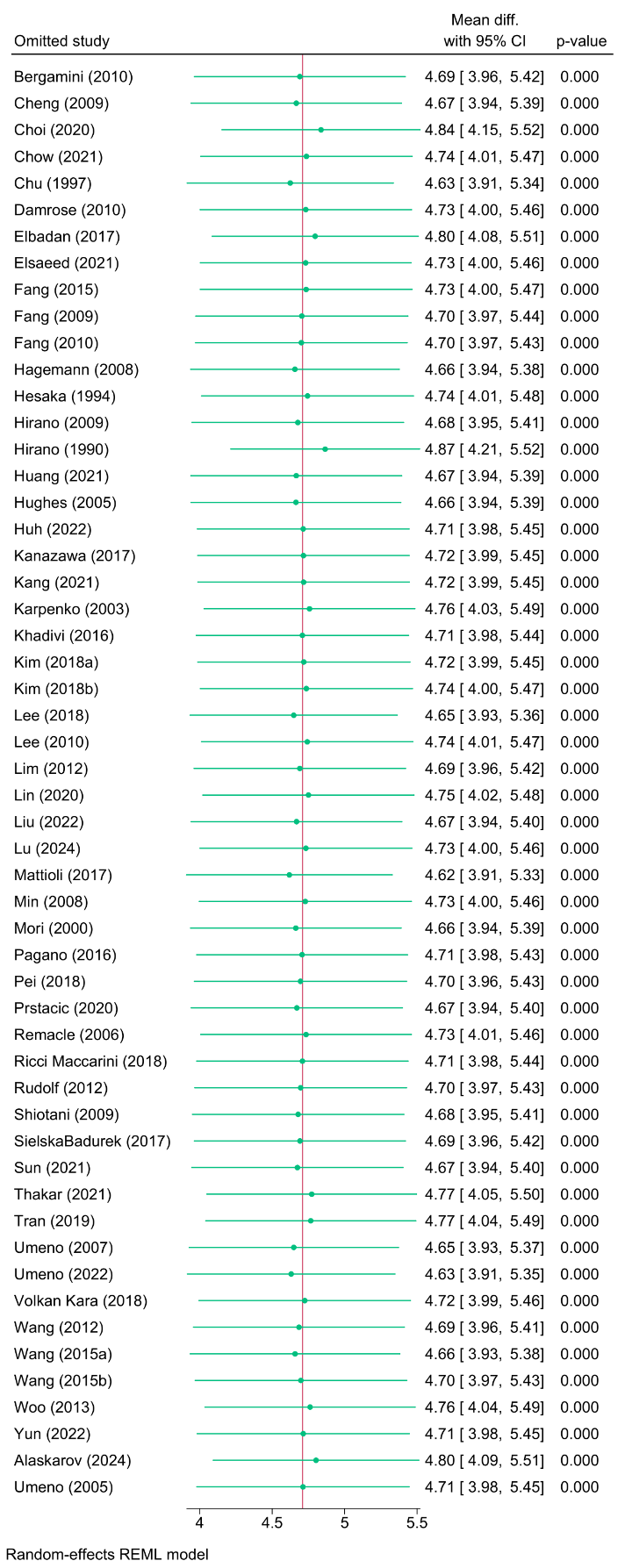


**SDC, Figure 1.** Leave-one-out sensitivity analysis of the difference in maximum phonation time post-injection


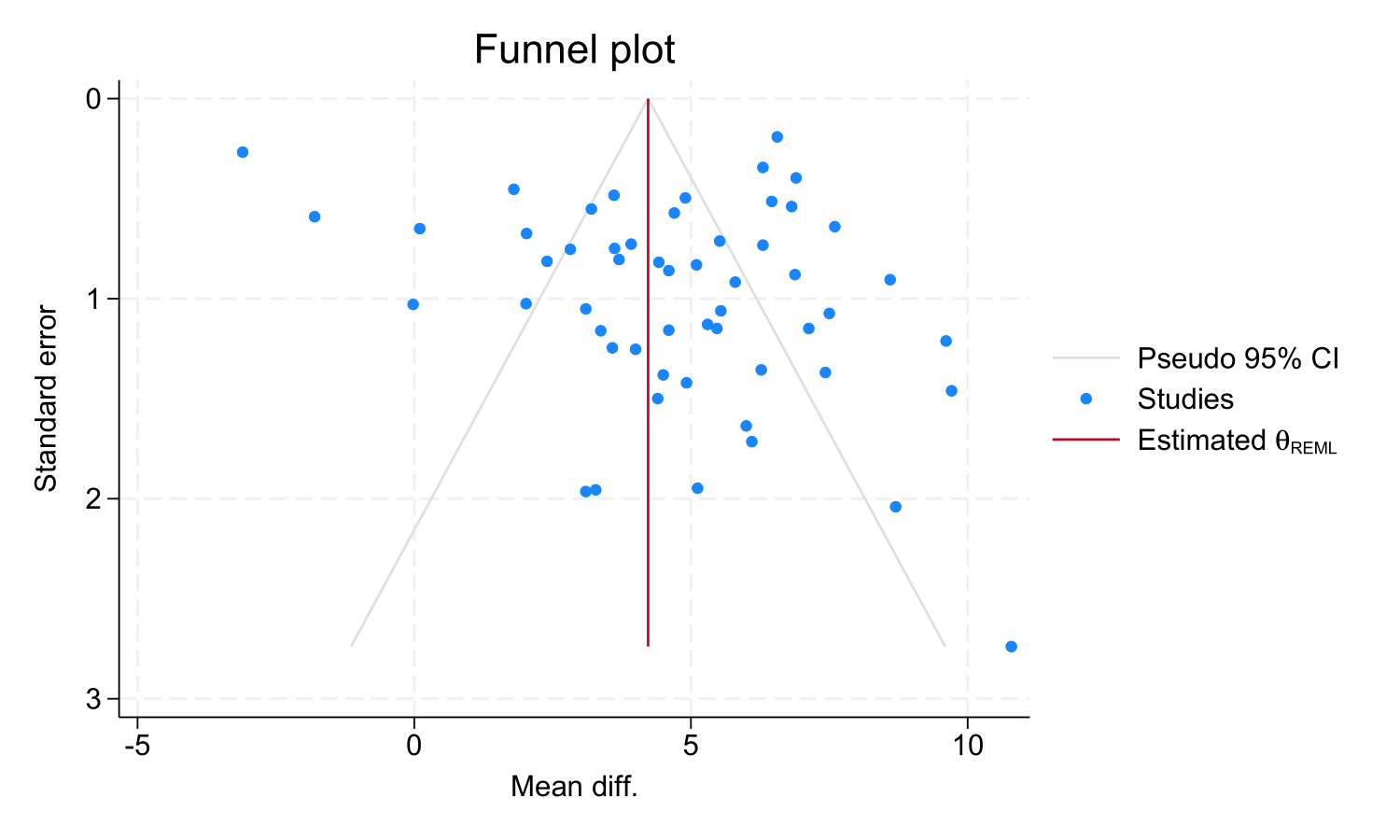


**SDC, Figure 2.** Funnel plot showing the risk of publication bias of maximum phonation time


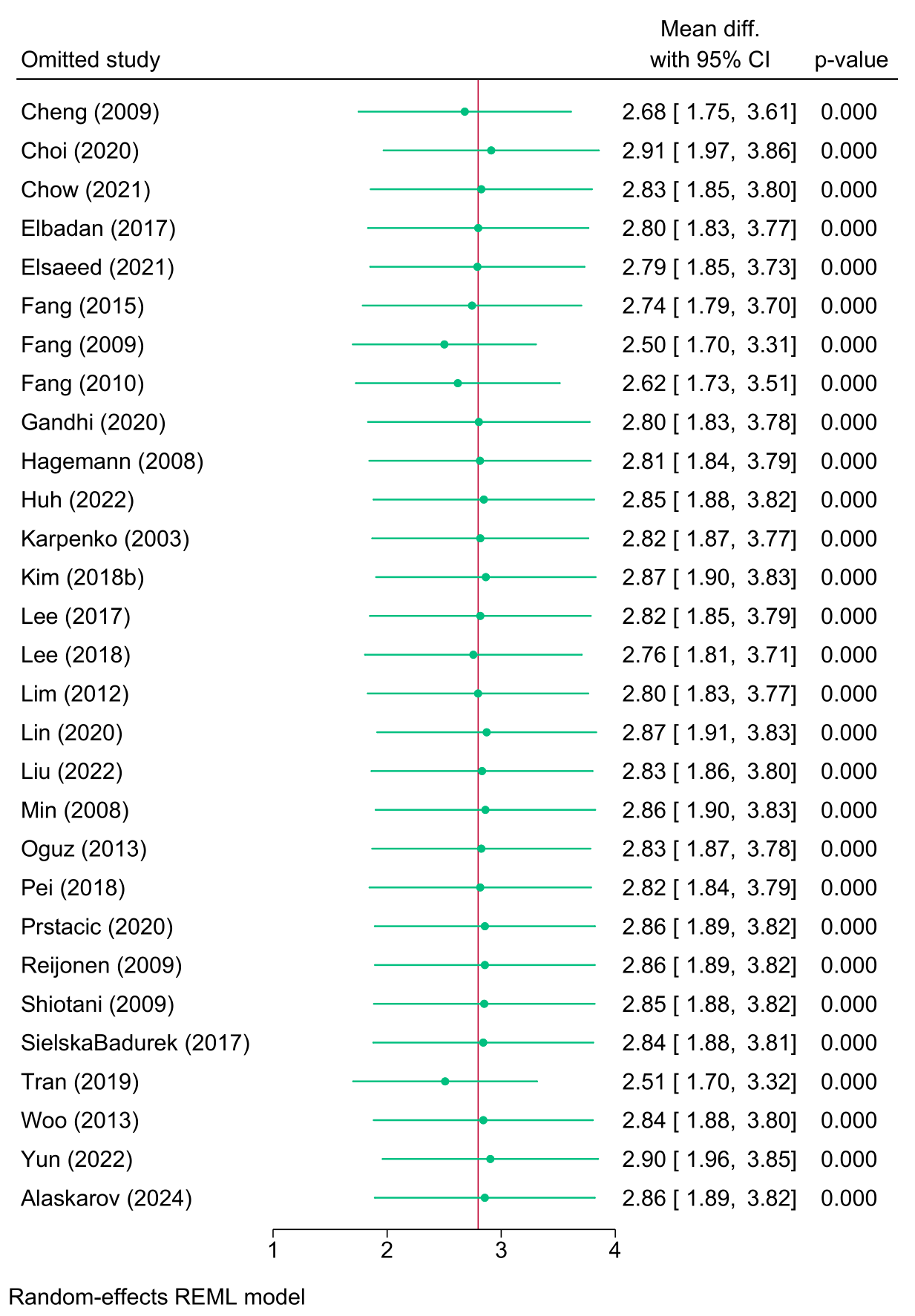


**SDC, Figure 3.** Leave-one-out sensitivity analysis of the difference in harmonics-to-noise ratio post-injection


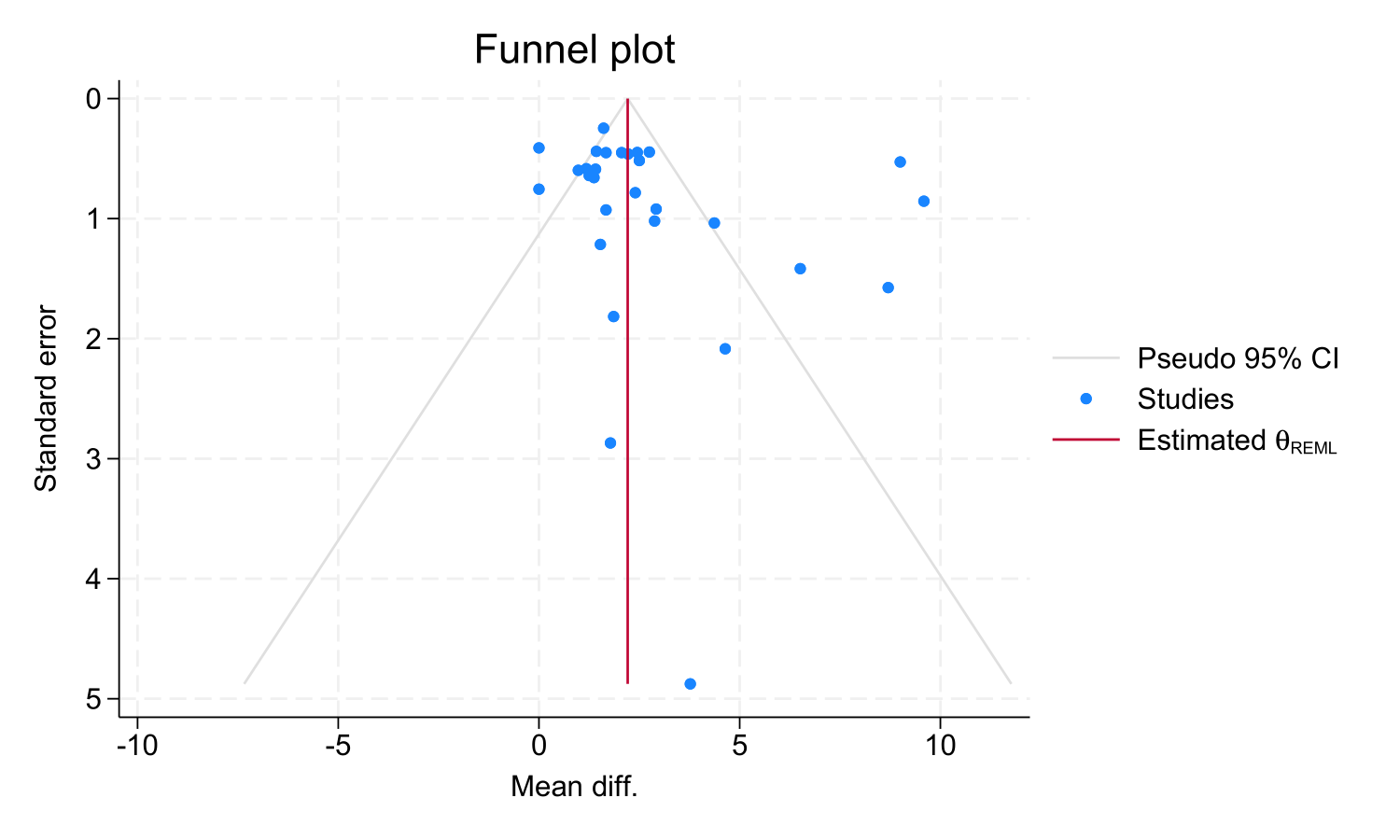


**SDC, Figure 4.** Funnel plot showing the risk of publication bias of harmonics-to-noise ratio


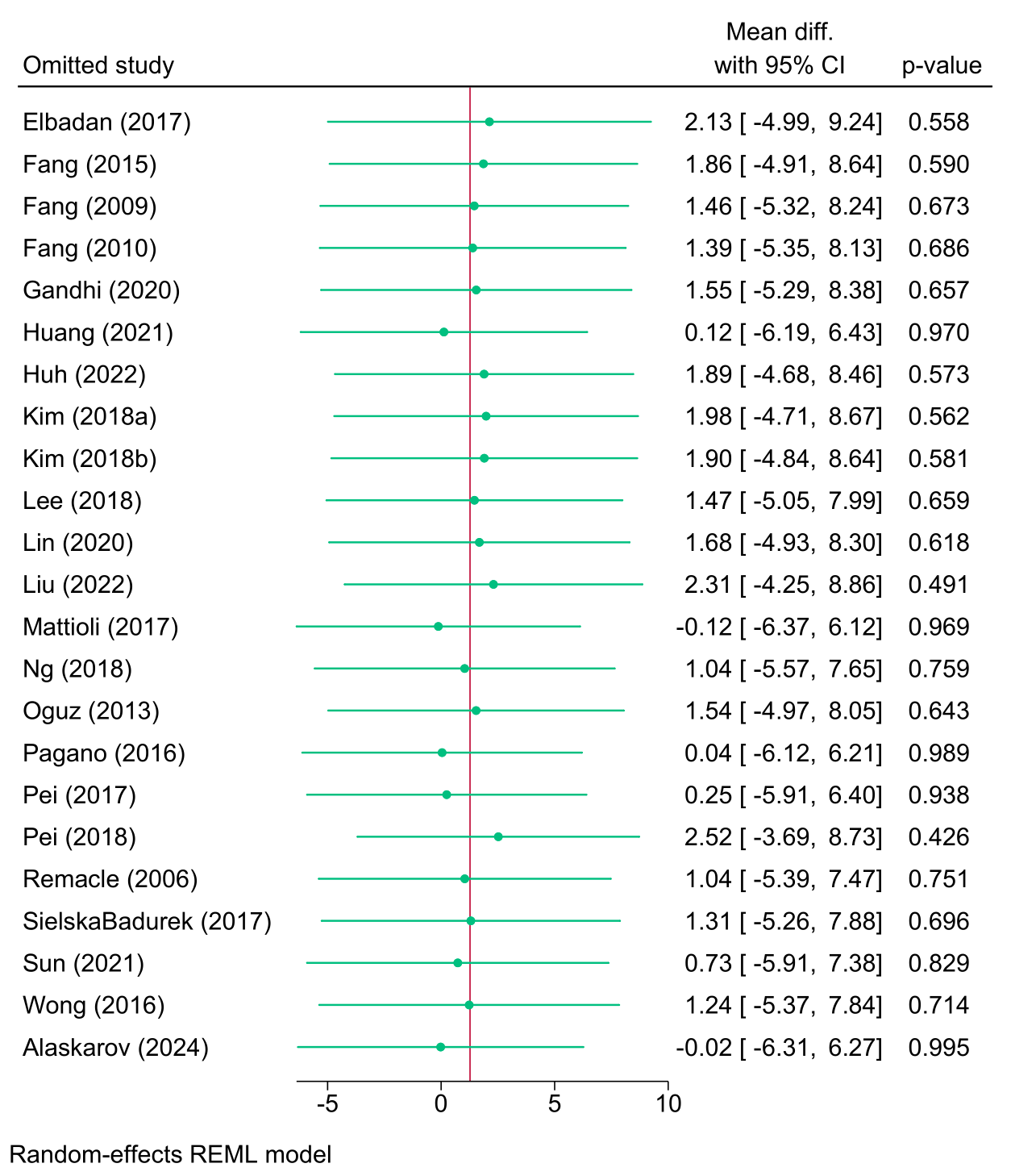


**SDC, Figure 5.** Leave-one-out sensitivity analysis of the difference in fundamental frequency post-injection


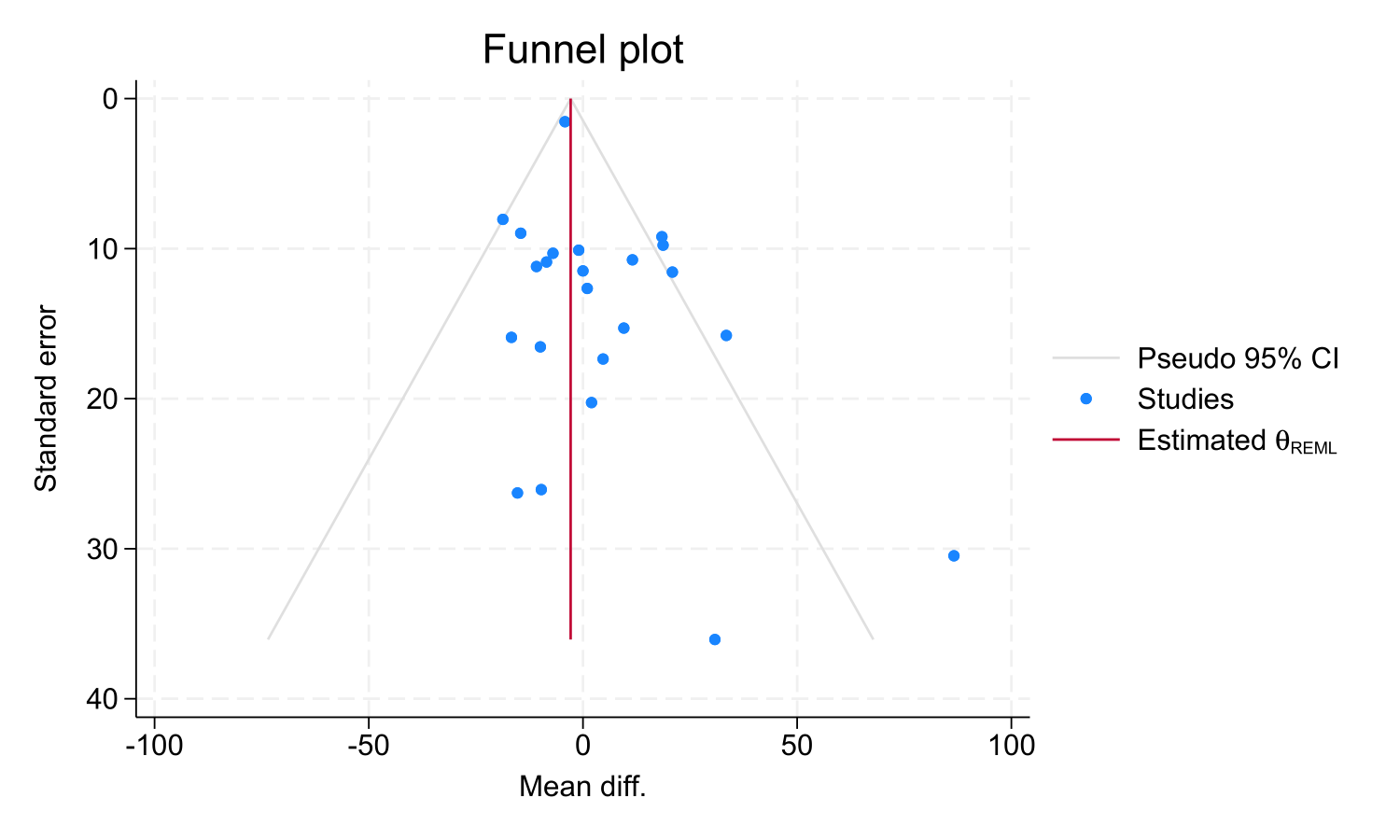


**SDC, Figure 6.** Funnel plot showing the risk of publication bias of fundamental frequency


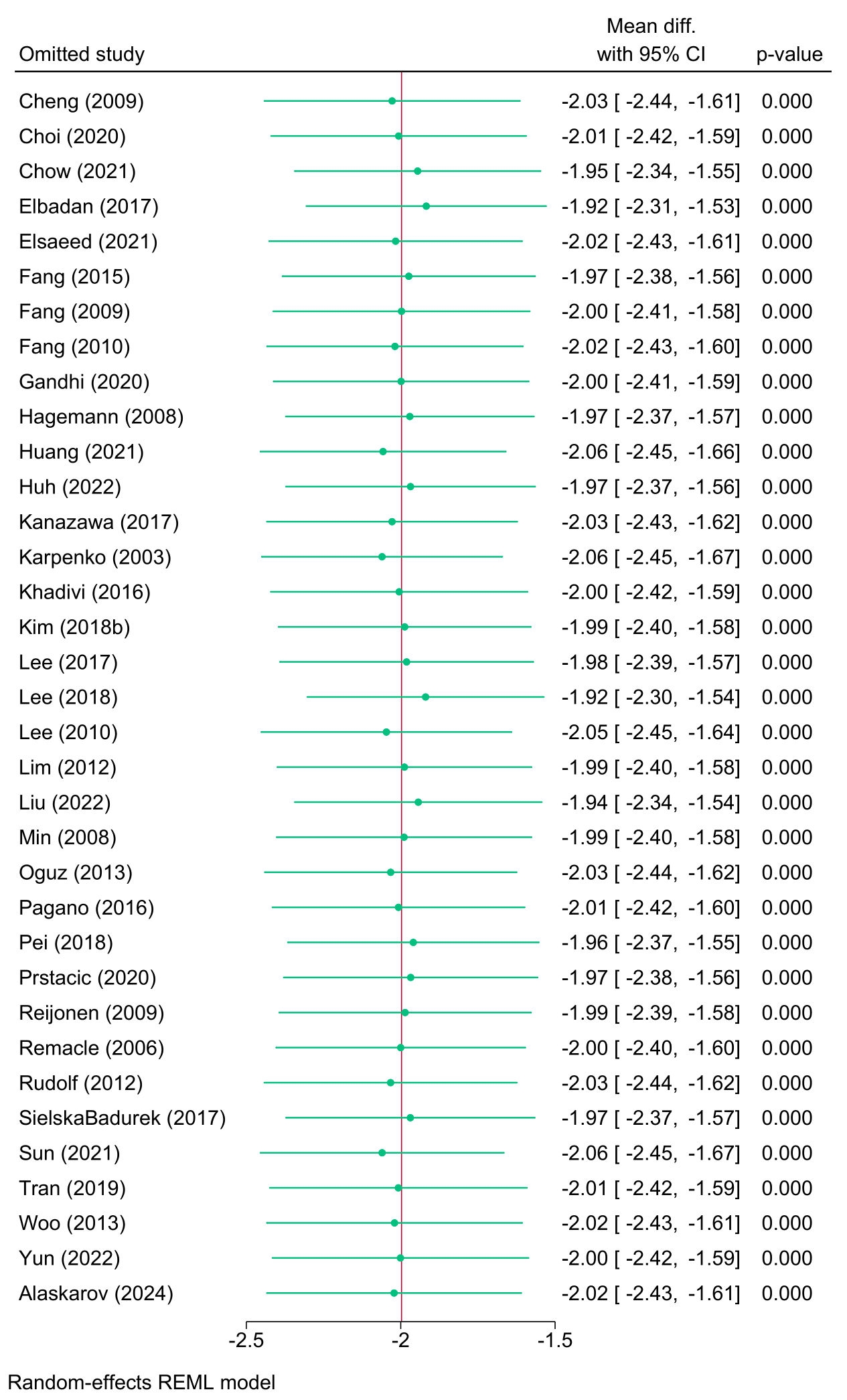


**SDC, Figure 7.** Leave-one-out sensitivity analysis of the difference in Jitter (%) post-injection


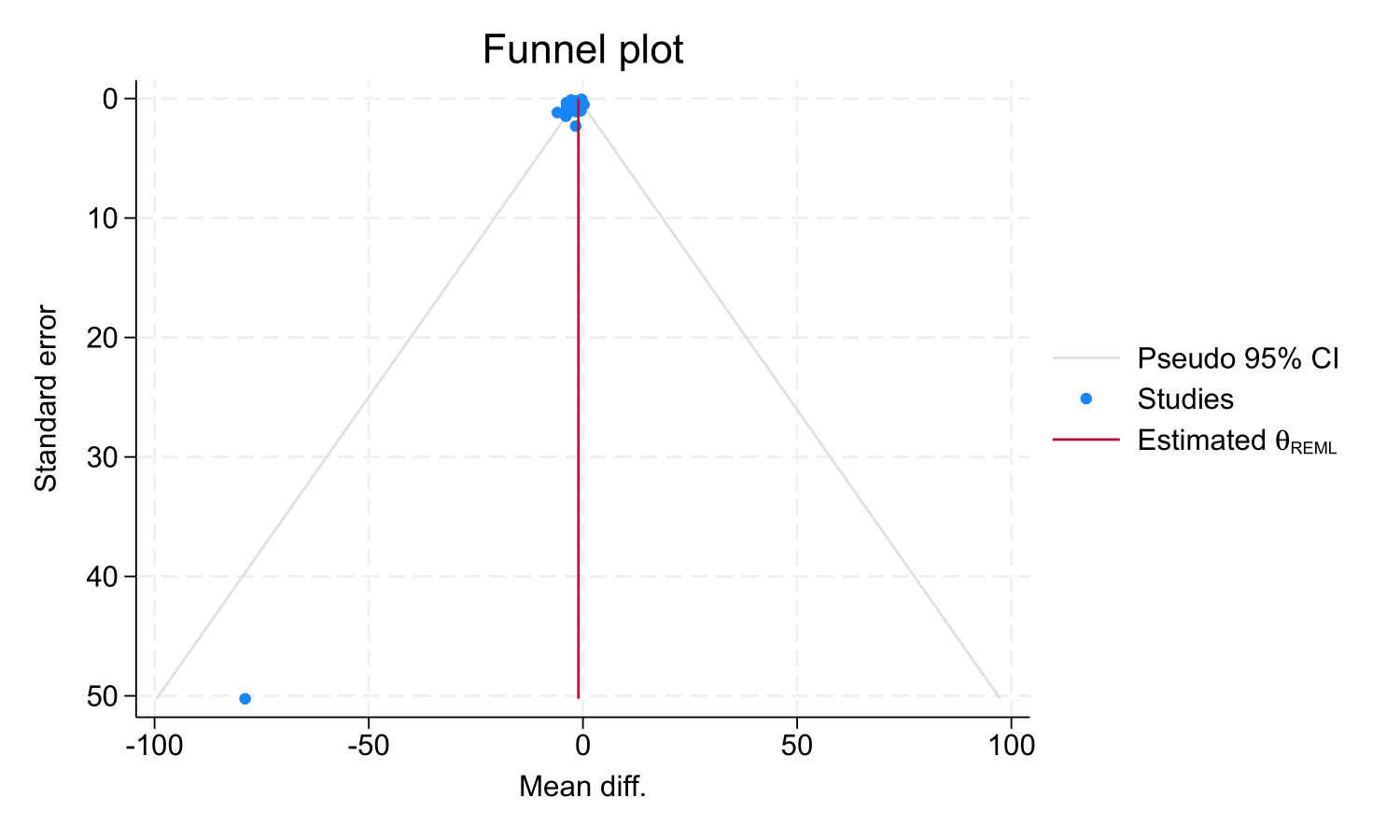


**SDC, Figure 8.** Funnel plot showing the risk of publication bias of Jitter (%)


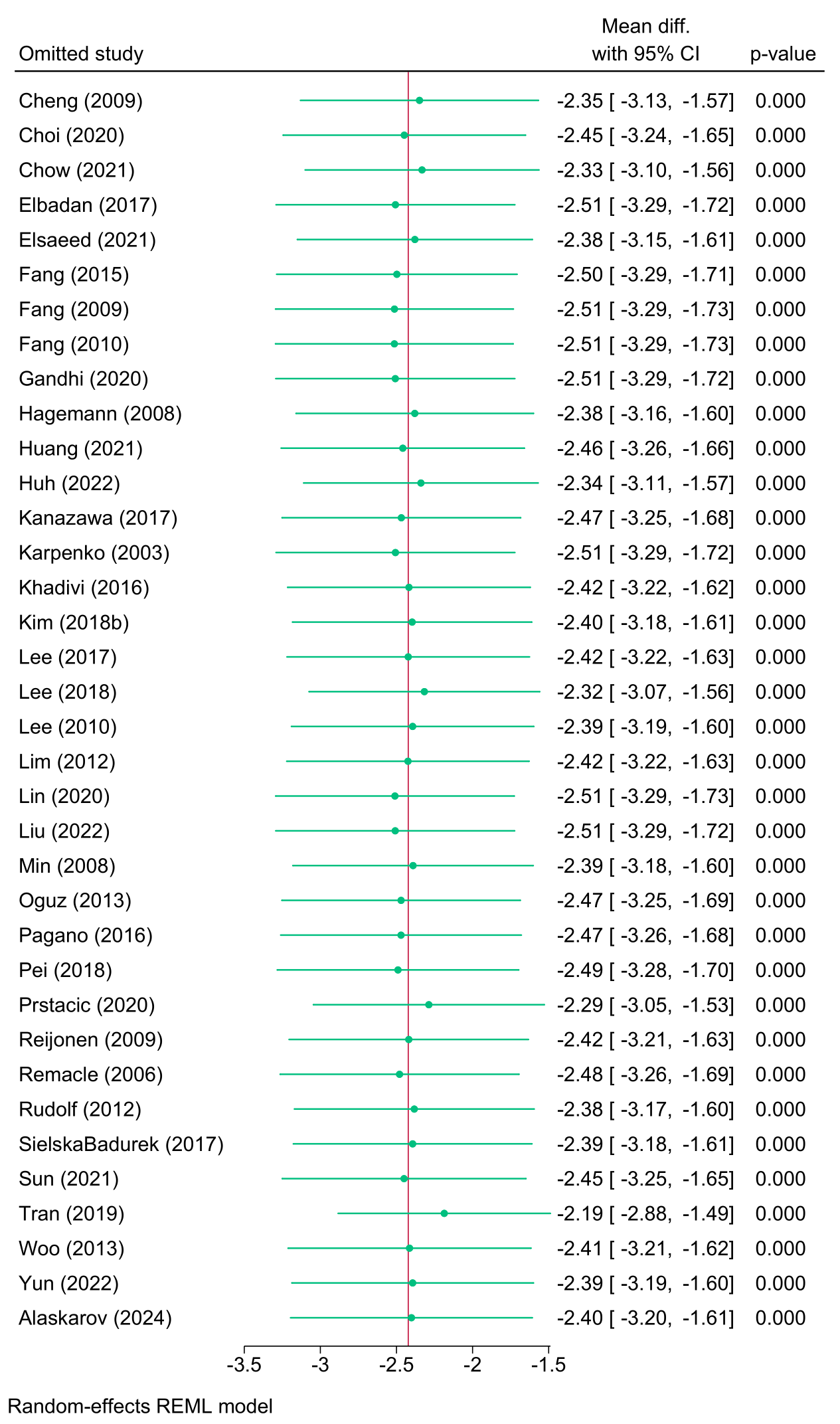


**SDC, Figure 9.** Leave-one-out sensitivity analysis of the difference in Shimmer (%) post-injection


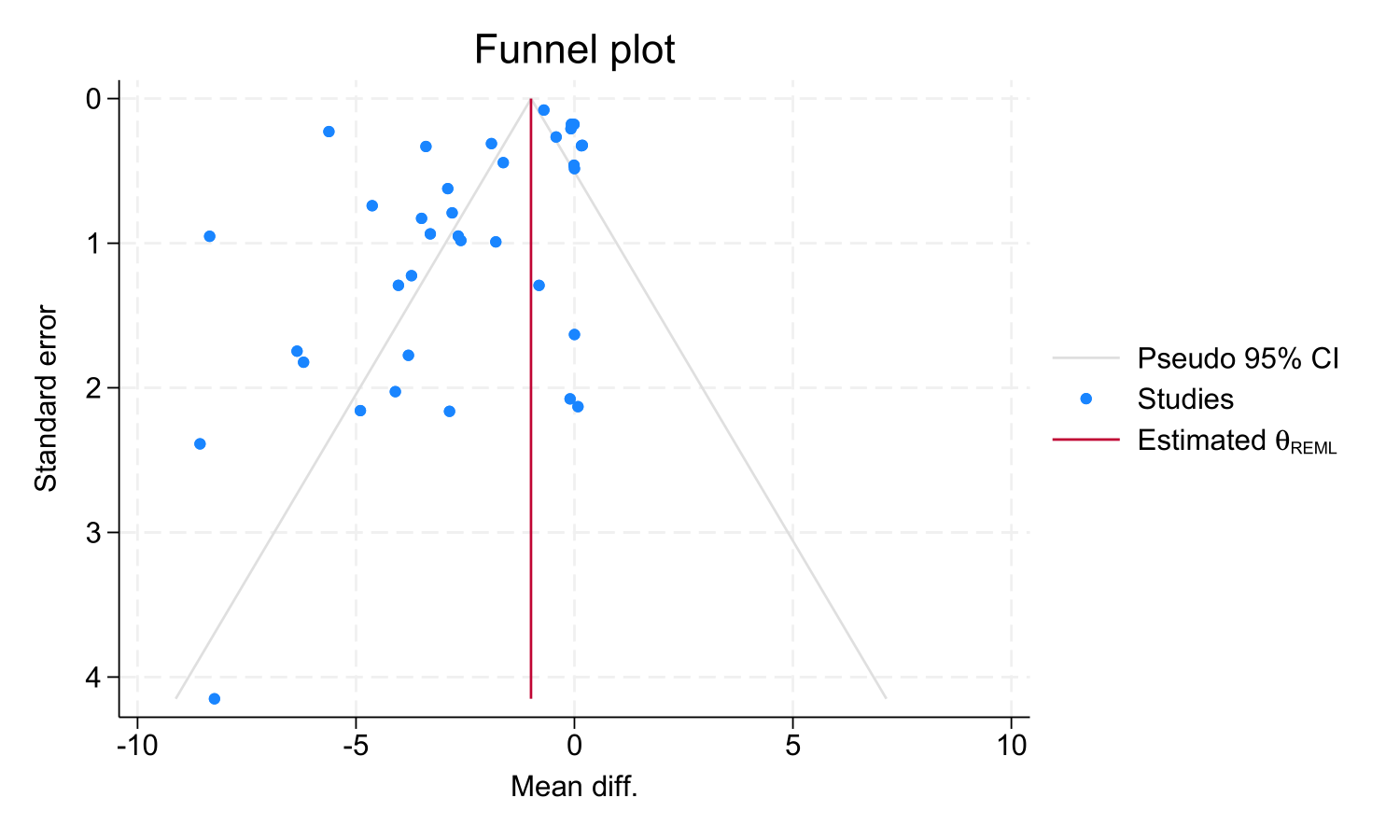


**SDC, Figure 10.** Funnel plot showing the risk of publication bias of Shimmer (%)


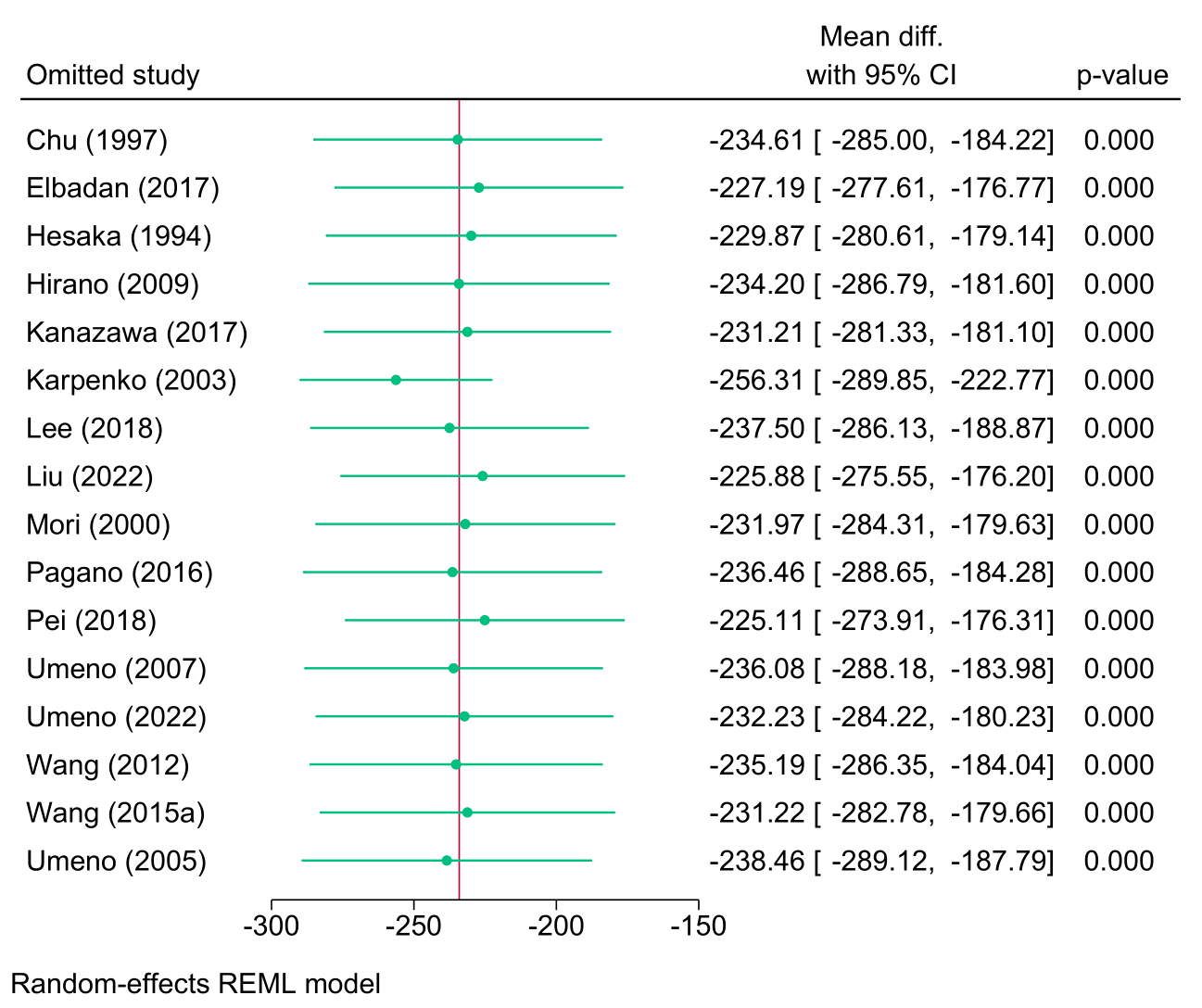


**SDC, Figure 11.** Leave-one-out sensitivity analysis of the difference in mean flow rate post-injection


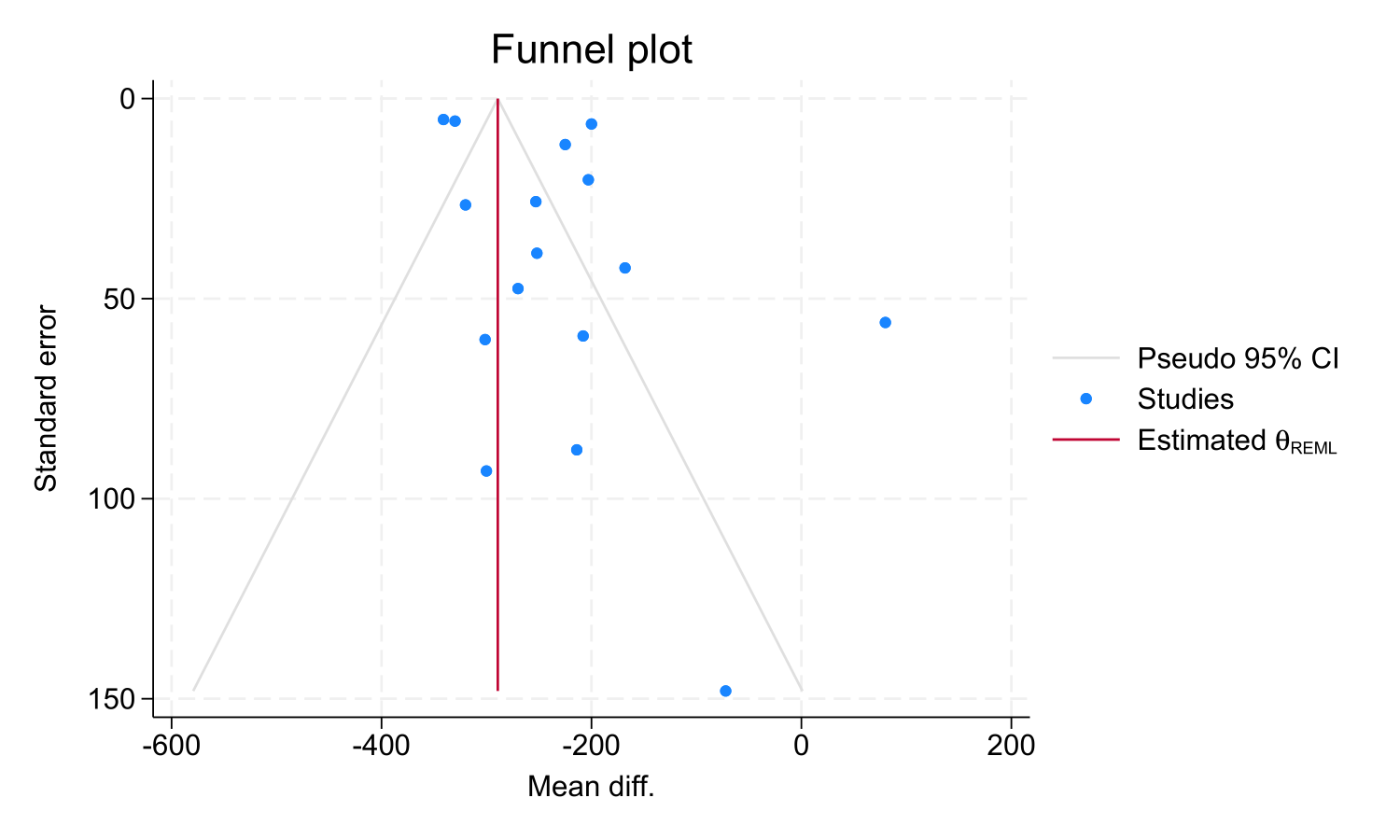


**SDC, Figure 12.** Funnel plot showing the risk of publication bias of mean flow rate


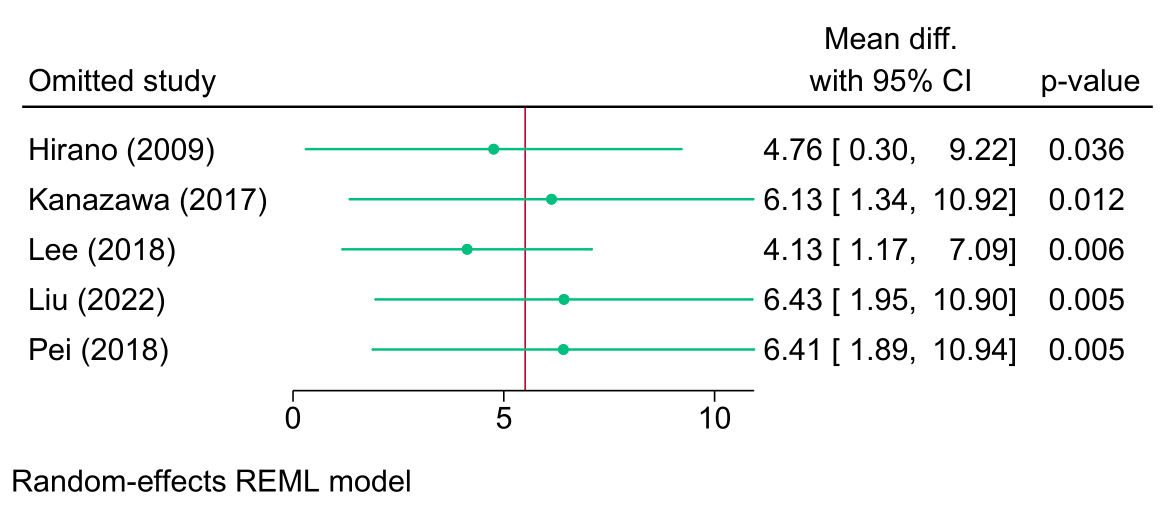


**SDC, Figure 13.** Leave-one-out sensitivity analysis of the difference in sound pressure level post-injection


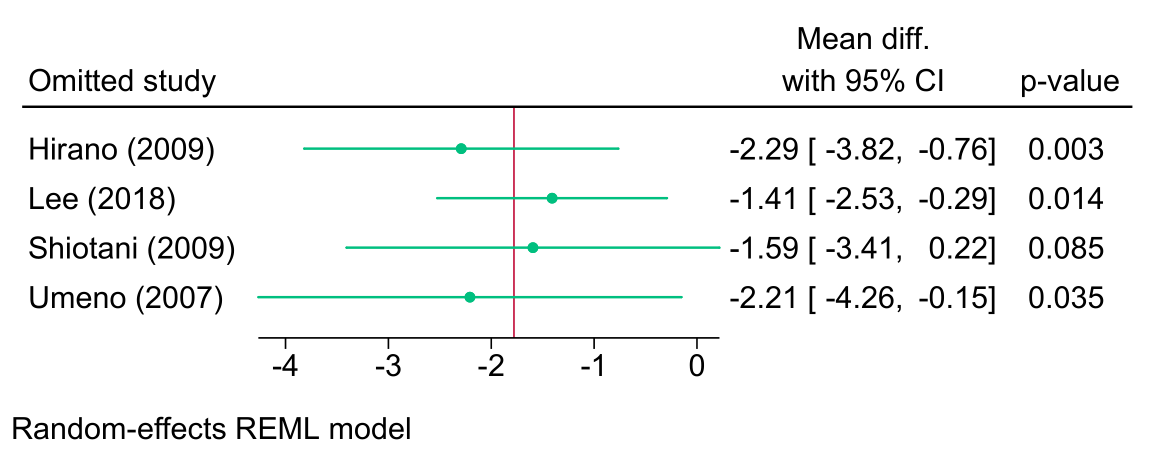


**SDC, Figure 14.** Leave-one-out sensitivity analysis of the difference in period perturbation quotient post-injection


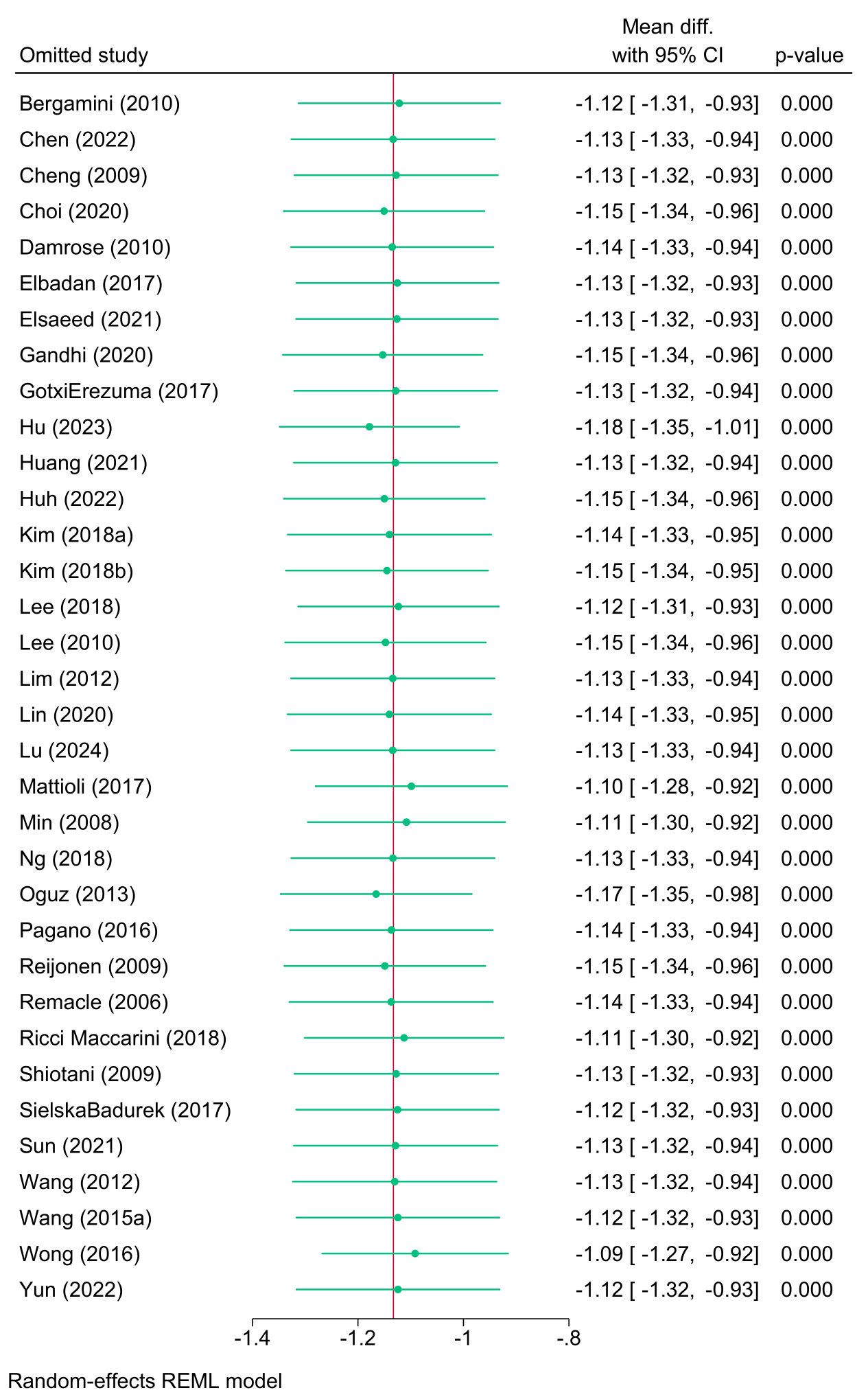


**SDC, Figure 15.** Leave-one-out sensitivity analysis of the difference in grade of dysphonia post-injection


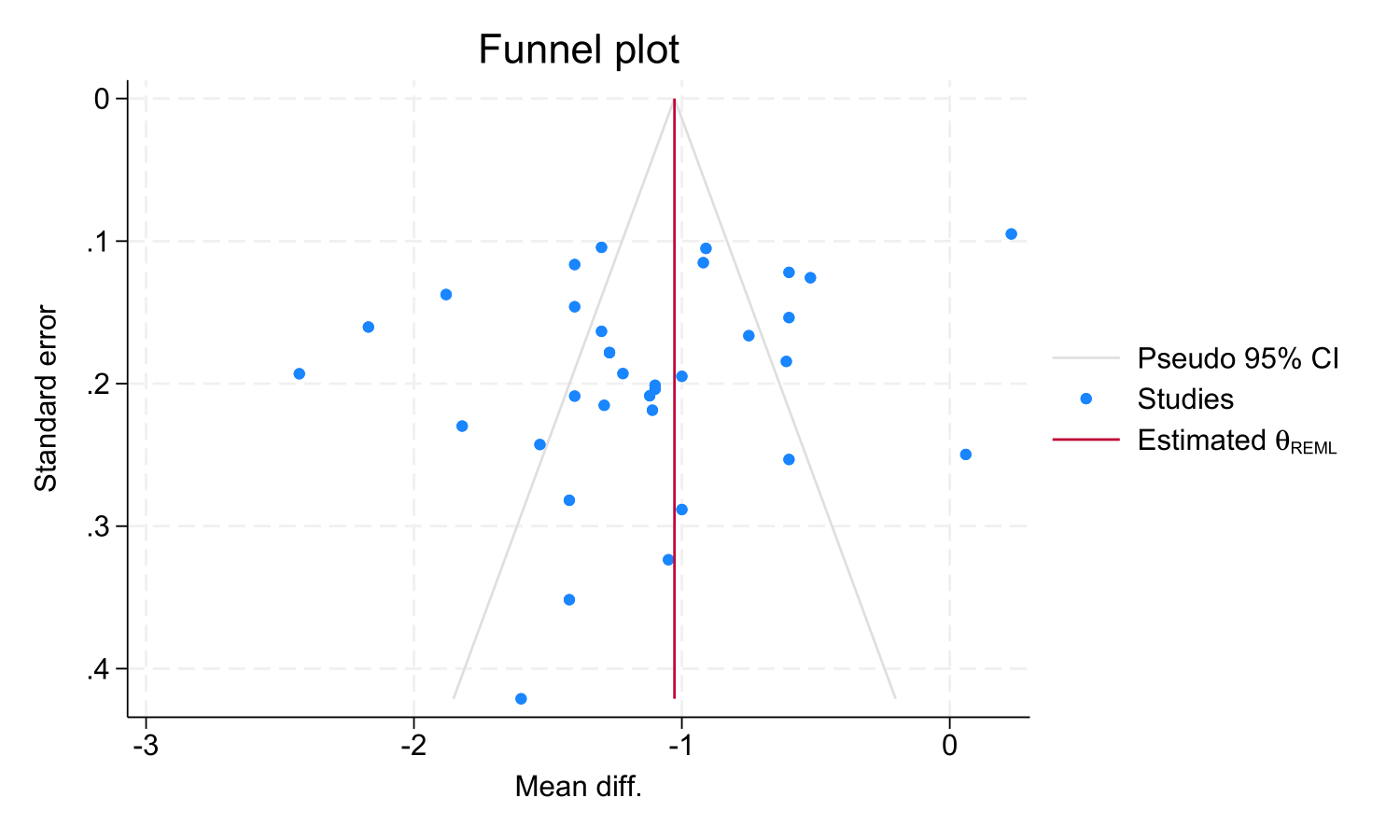


**SDC, Figure 16.** Funnel plot showing the risk of publication bias of grade of dysphonia


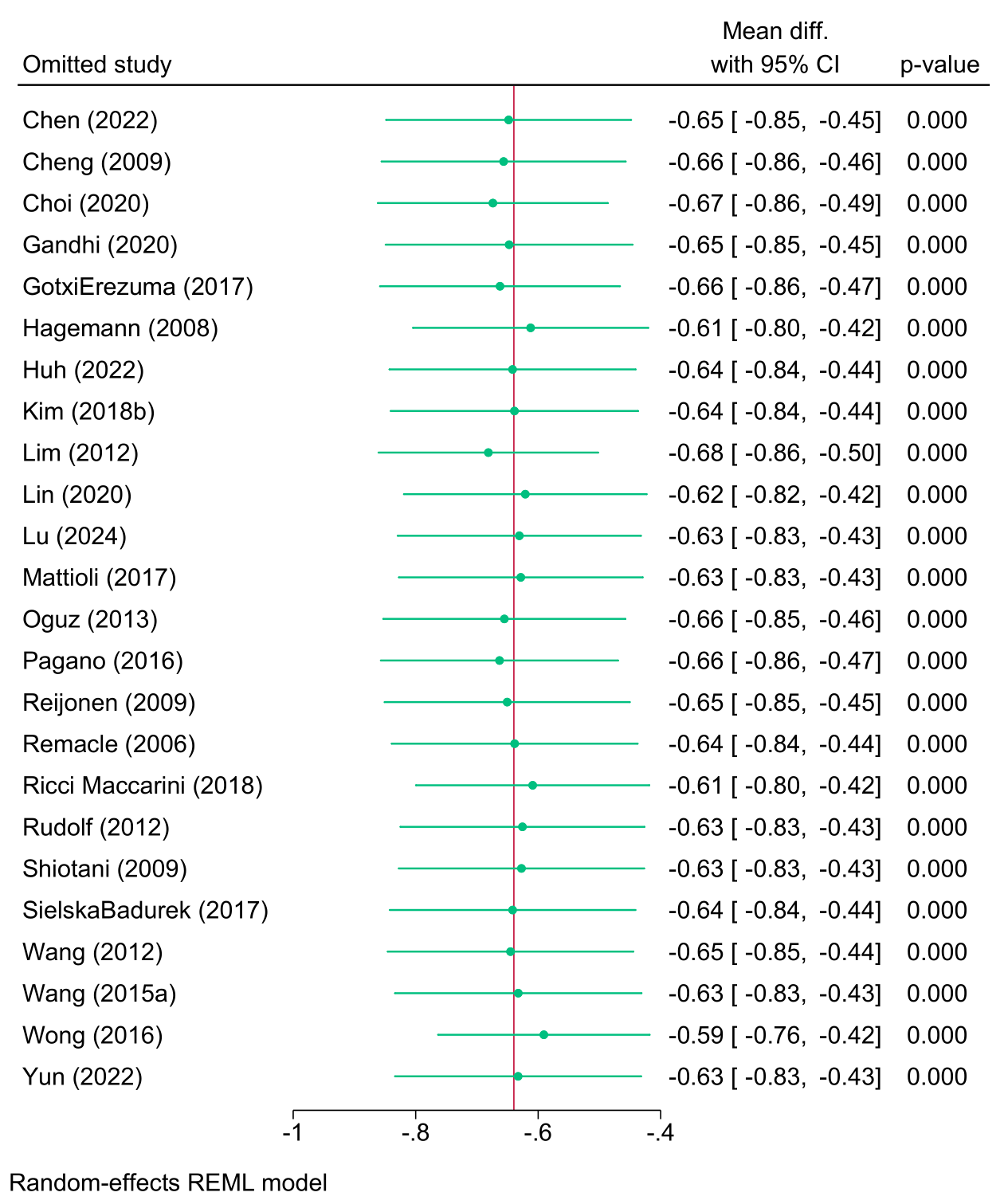


**SDC, Figure 17.** Leave-one-out sensitivity analysis of the difference in roughness post-injection


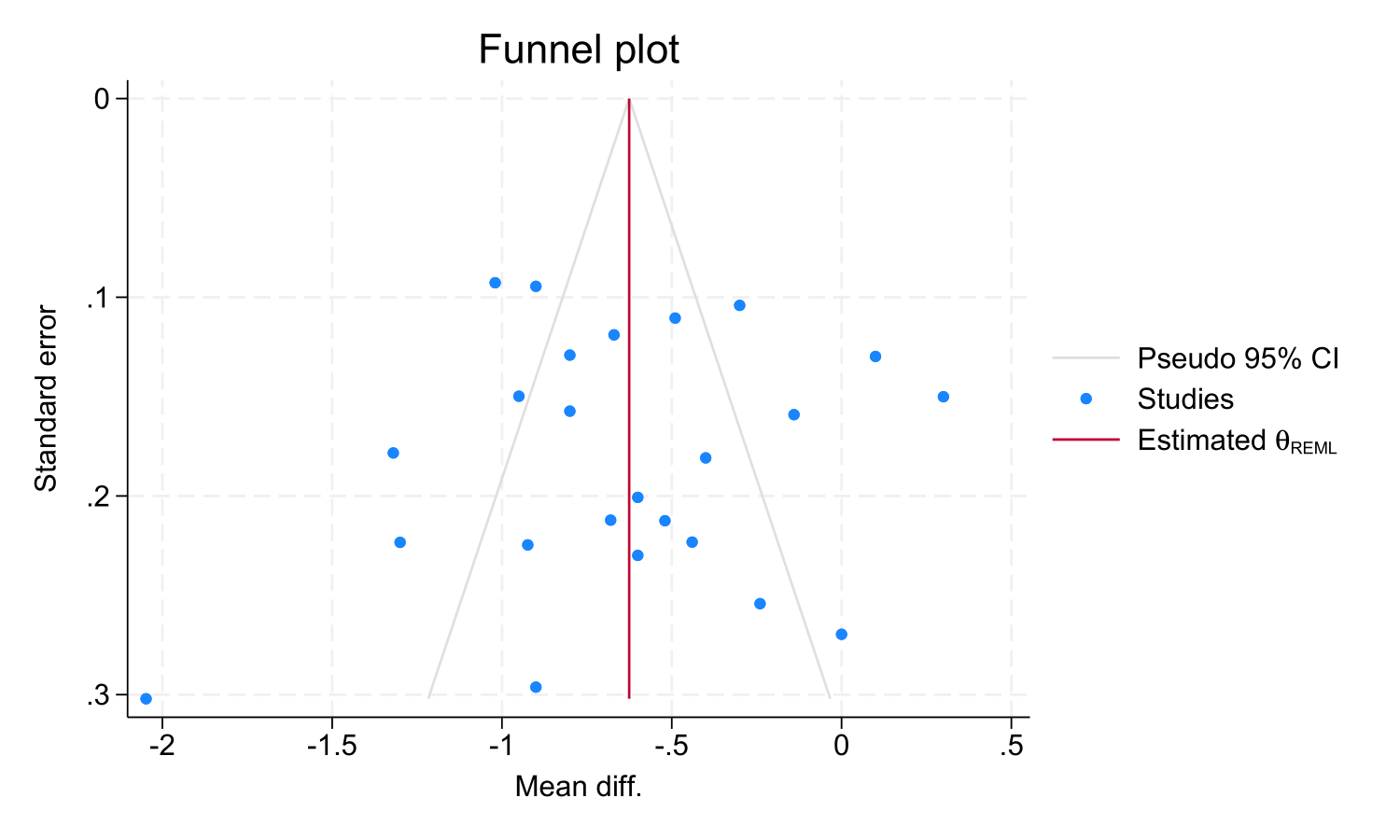


**SDC, Figure 18.** Funnel plot showing the risk of publication bias of roughness


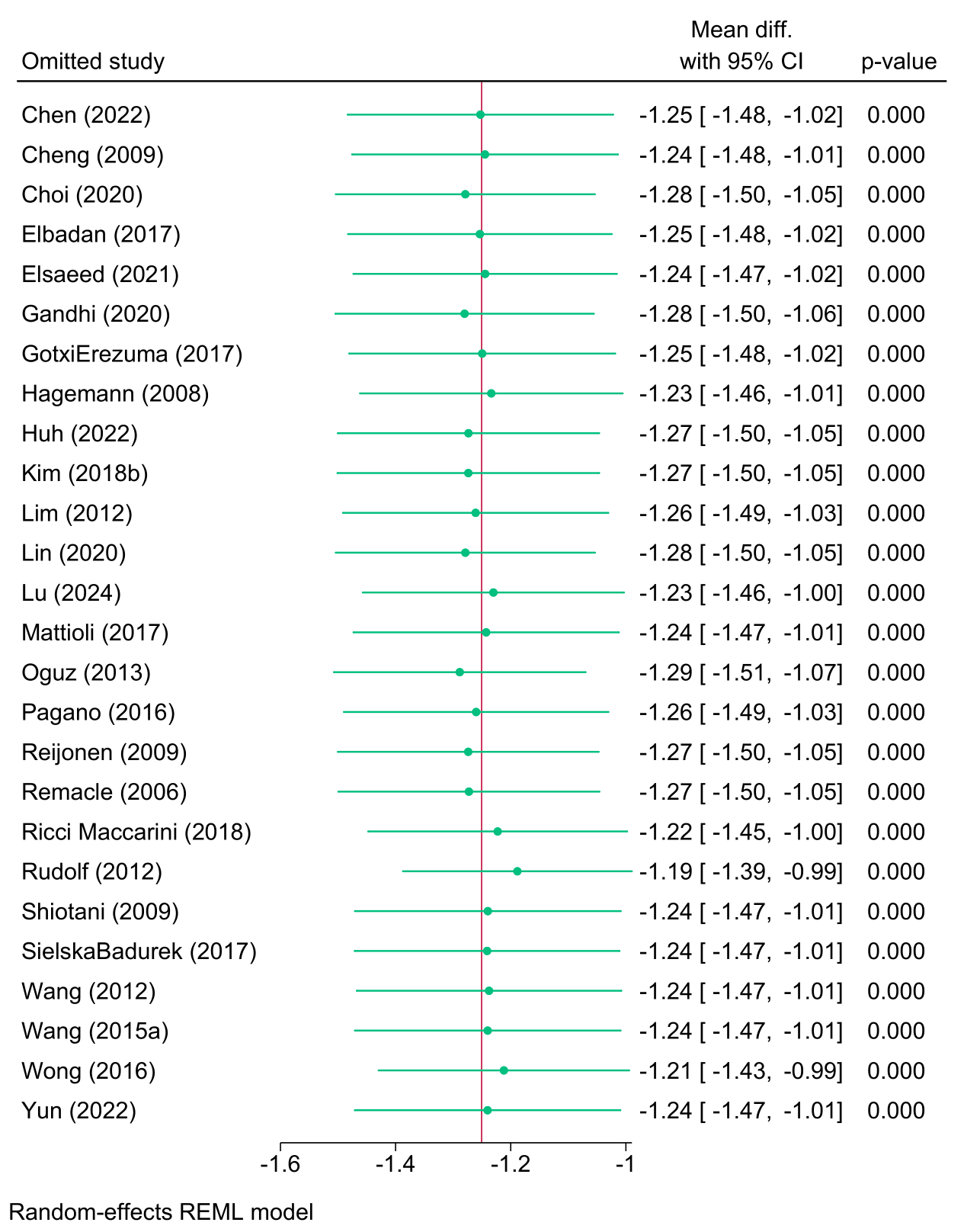


**SDC, Figure 19.** Leave-one-out sensitivity analysis of the difference in breathiness post-injection


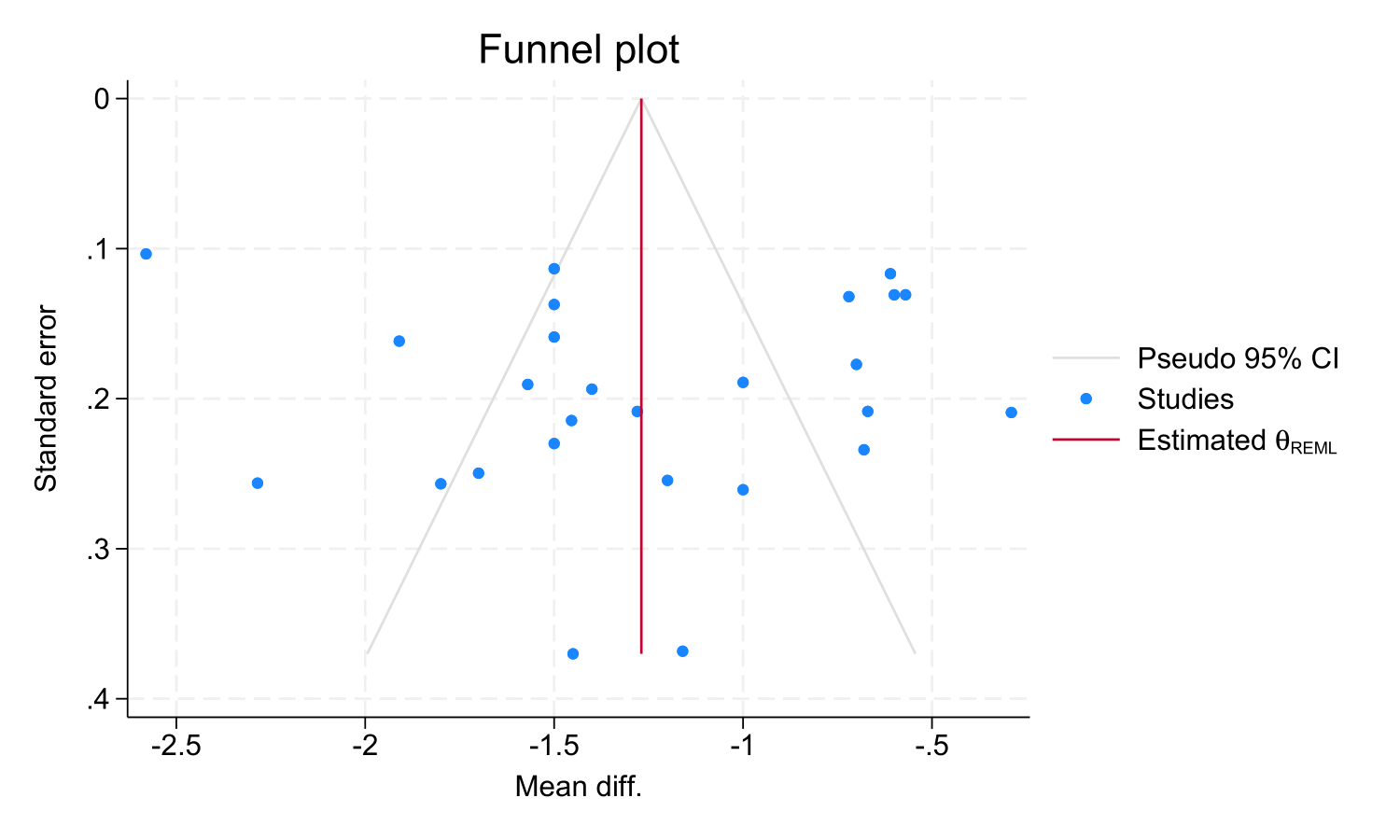


**SDC, Figure 20.** Funnel plot showing the risk of publication bias of breathiness


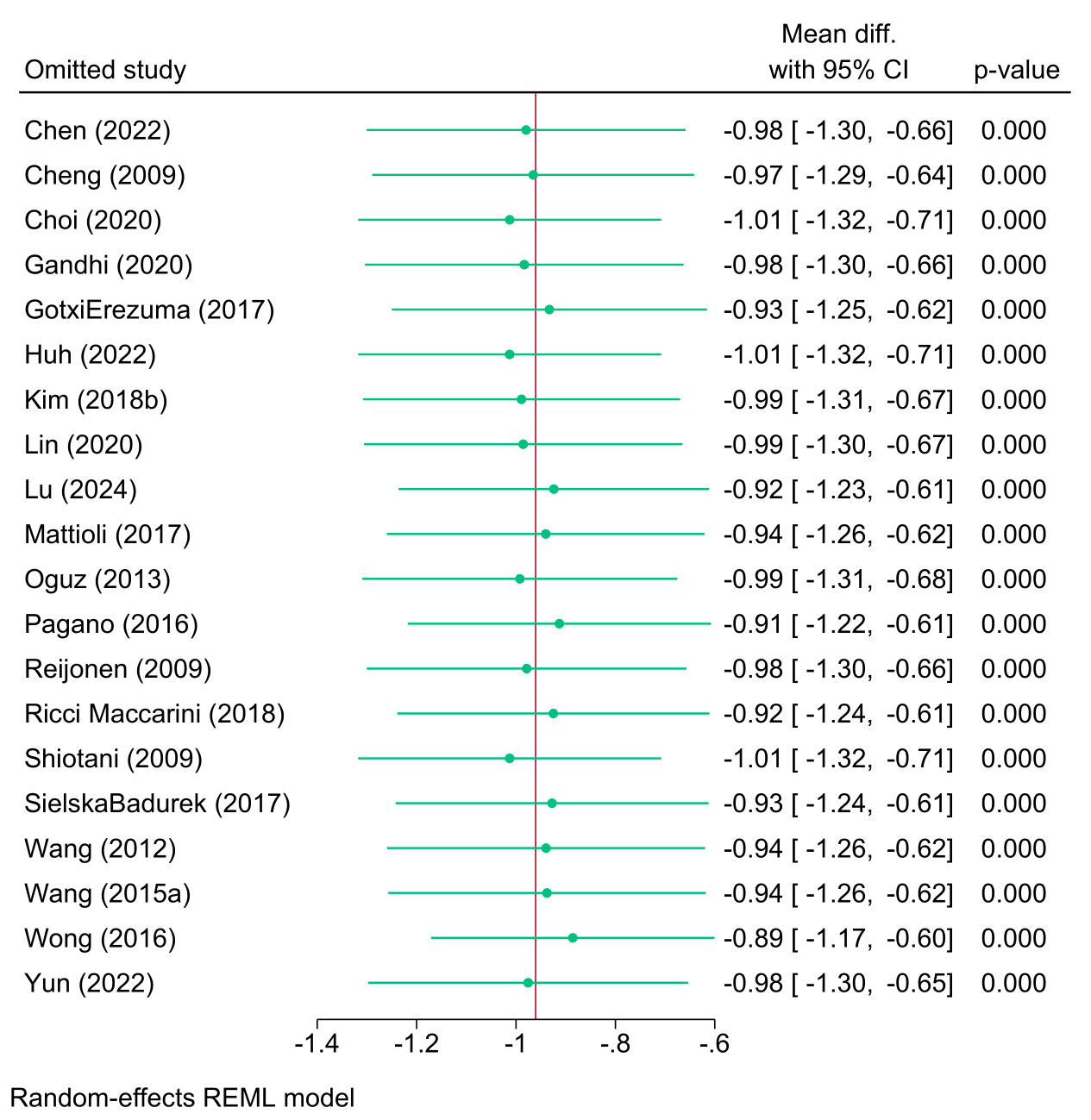


**SDC, Figure 21.** Leave-one-out sensitivity analysis of the difference in asthenia post-injection


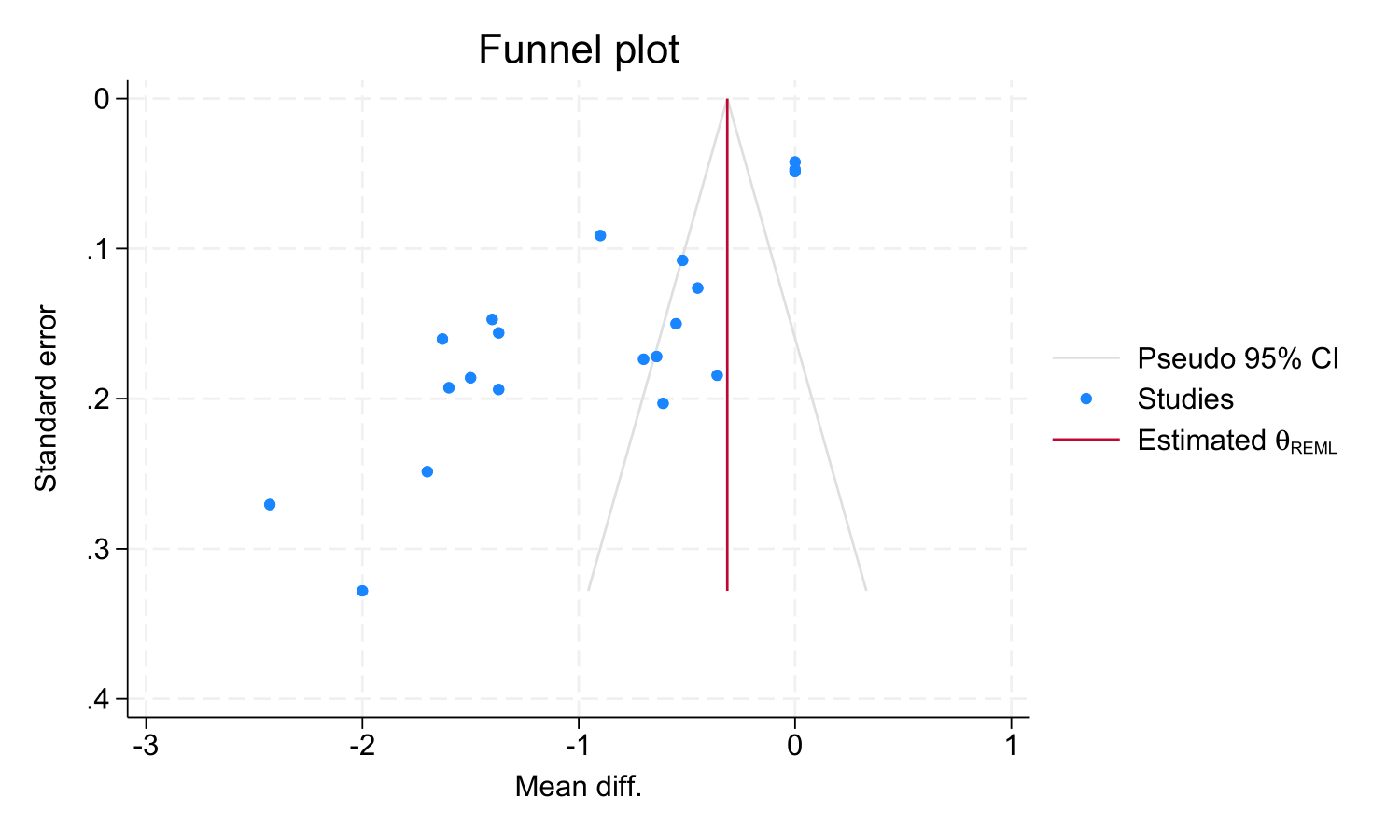


**SDC, Figure 22.** Funnel plot showing the risk of publication bias of asthenia


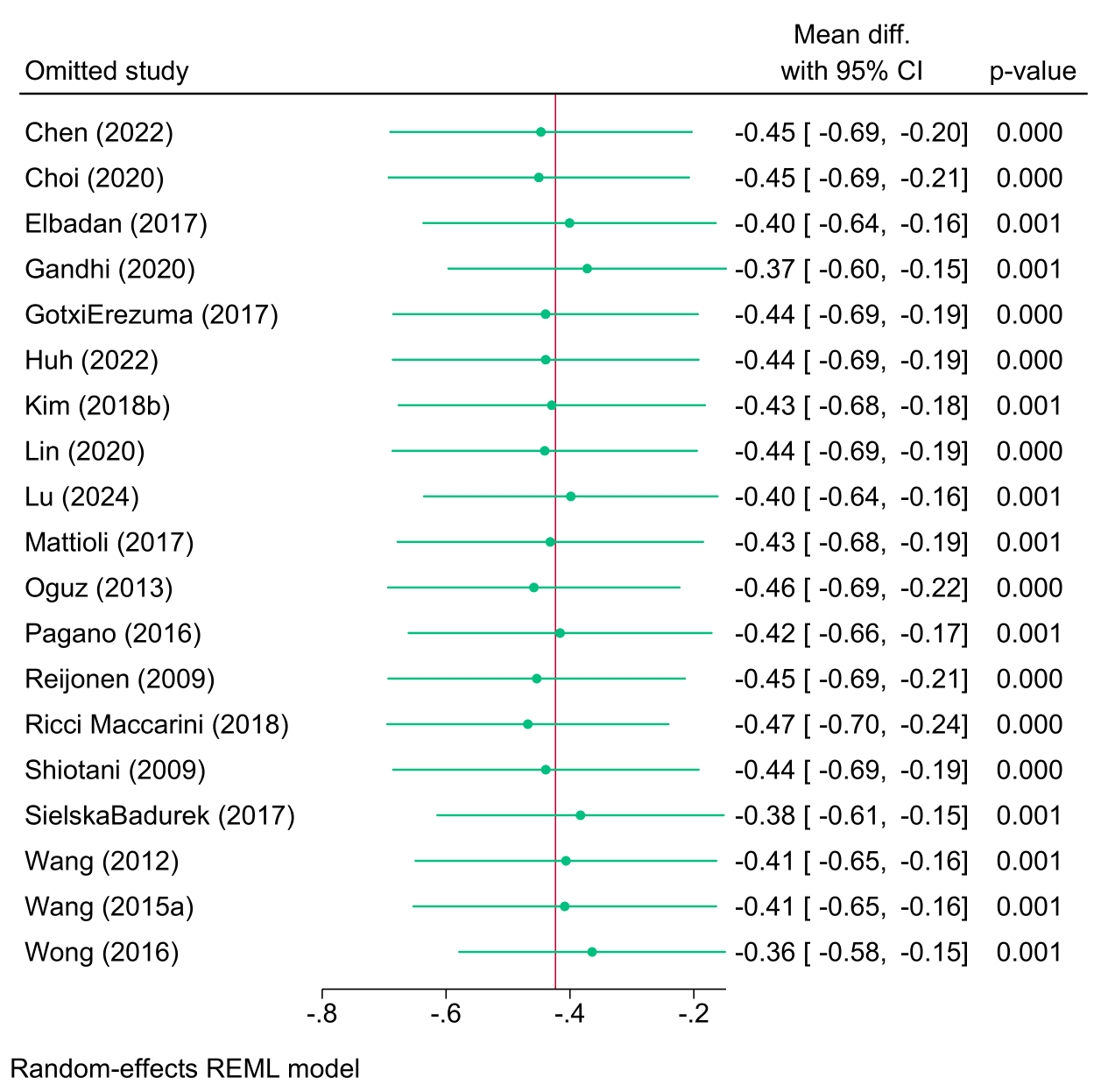


**SDC, Figure 23.** Leave-one-out sensitivity analysis of the difference in strain post-injection


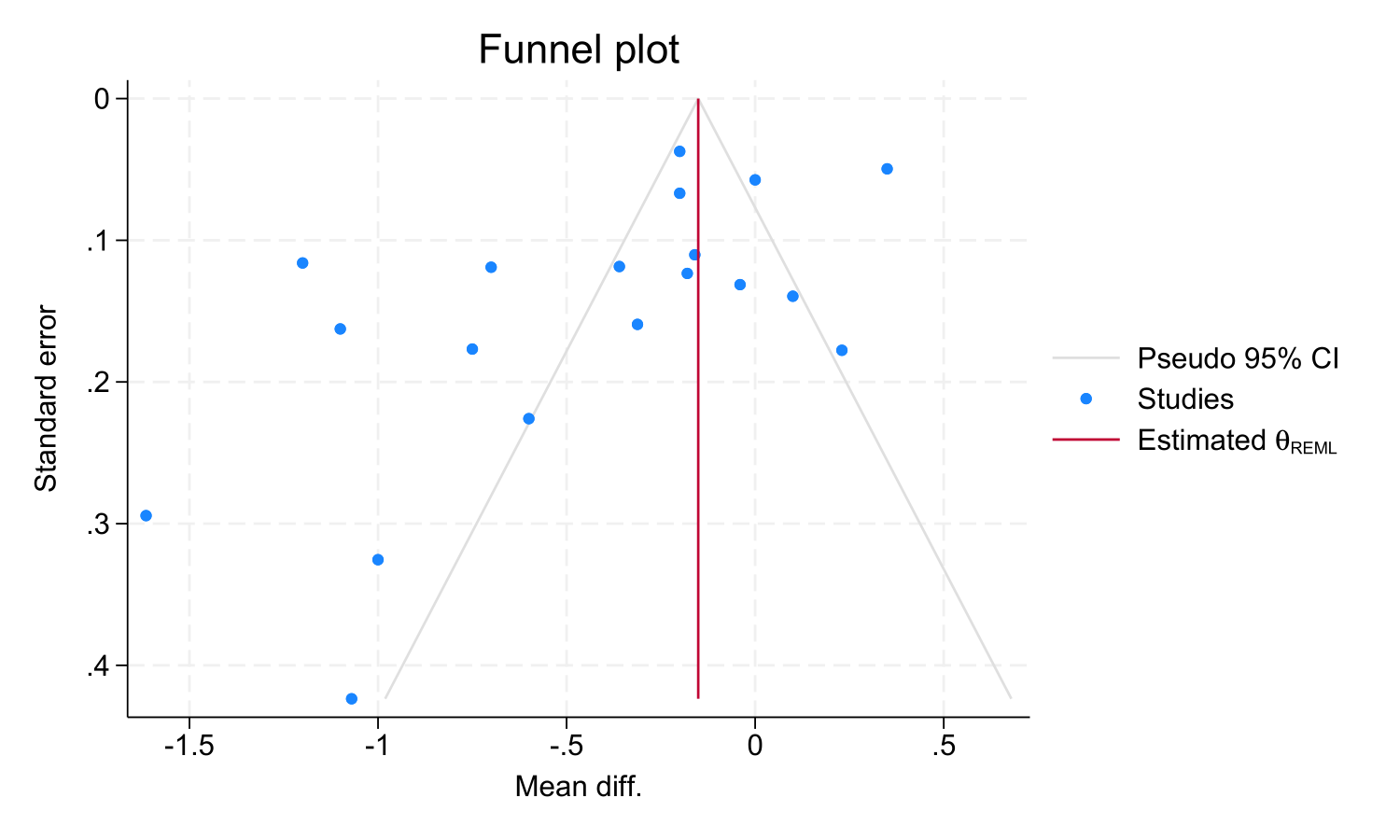


**SDC, Figure 24.** Funnel plot showing the risk of publication bias of strain


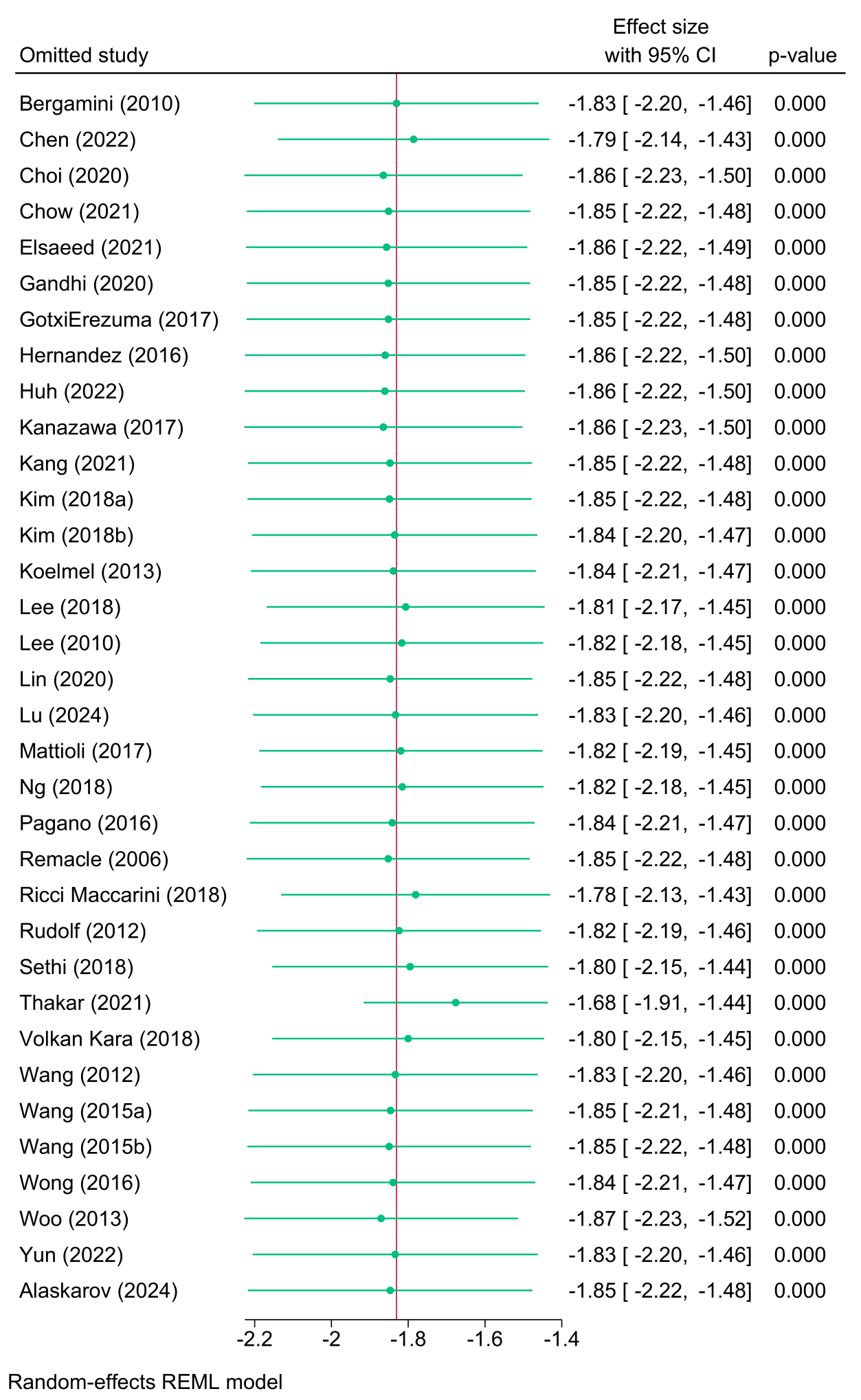


**SDC, Figure 25.** Leave-one-out sensitivity analysis of the difference in voice handicap index post-injection


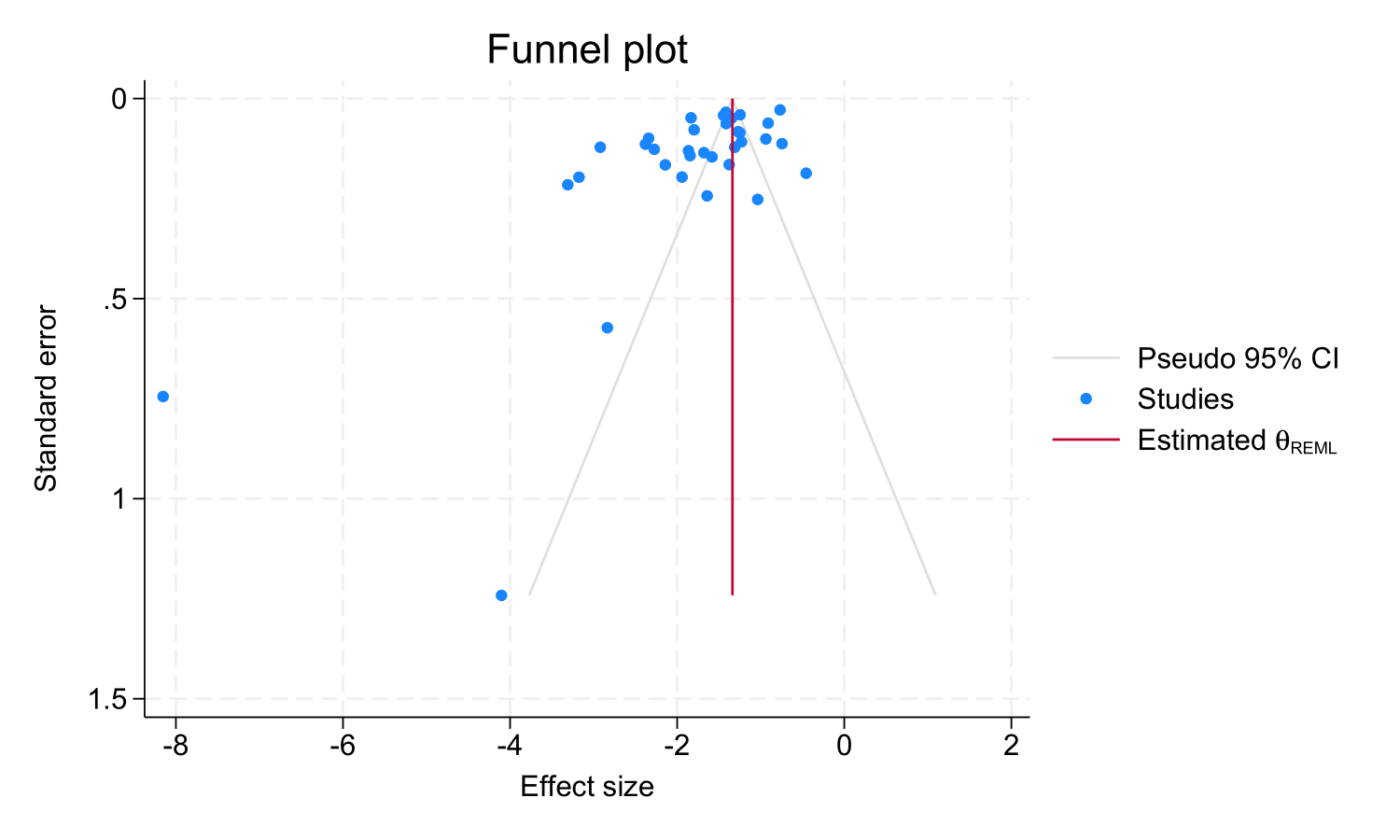


**SDC, Figure 26.** Funnel plot showing the risk of publication bias of voice handicap index


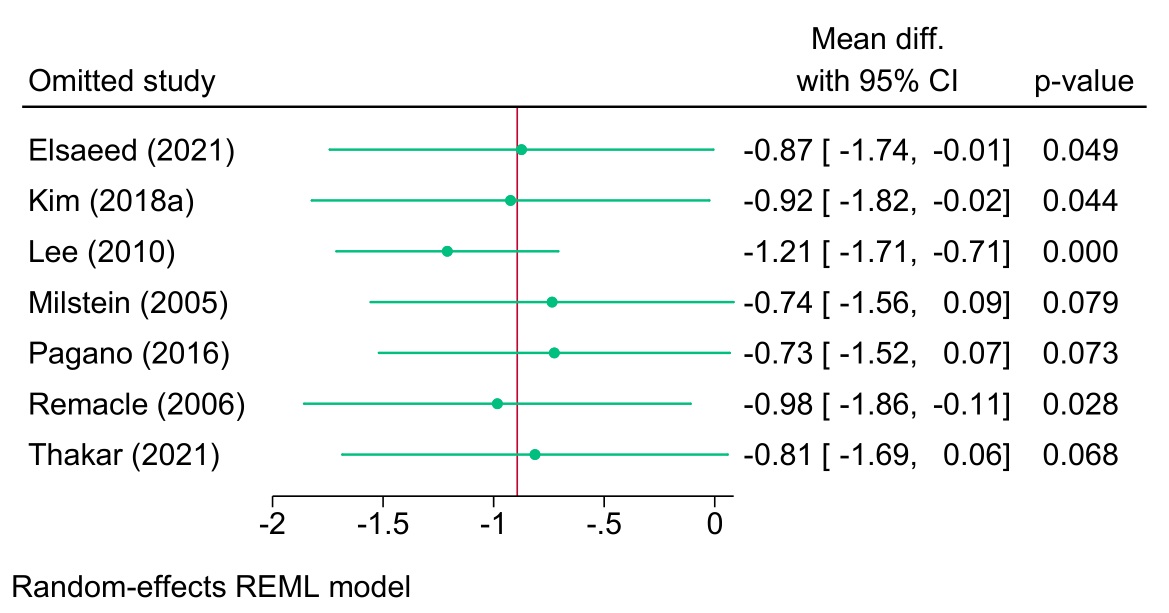


**SDC, Figure 27.** Leave-one-out sensitivity analysis of the difference in glottic gap post-injection


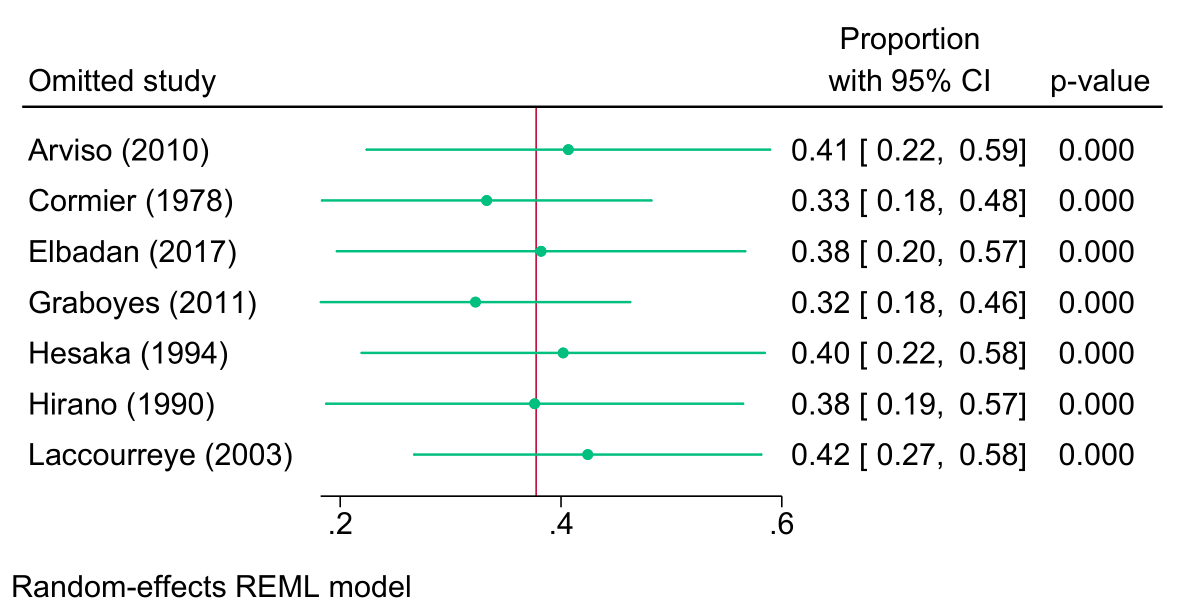


**SDC, Figure 28.** Leave-one-out sensitivity analysis of the prevalence of full recovery of paralysis post-injection


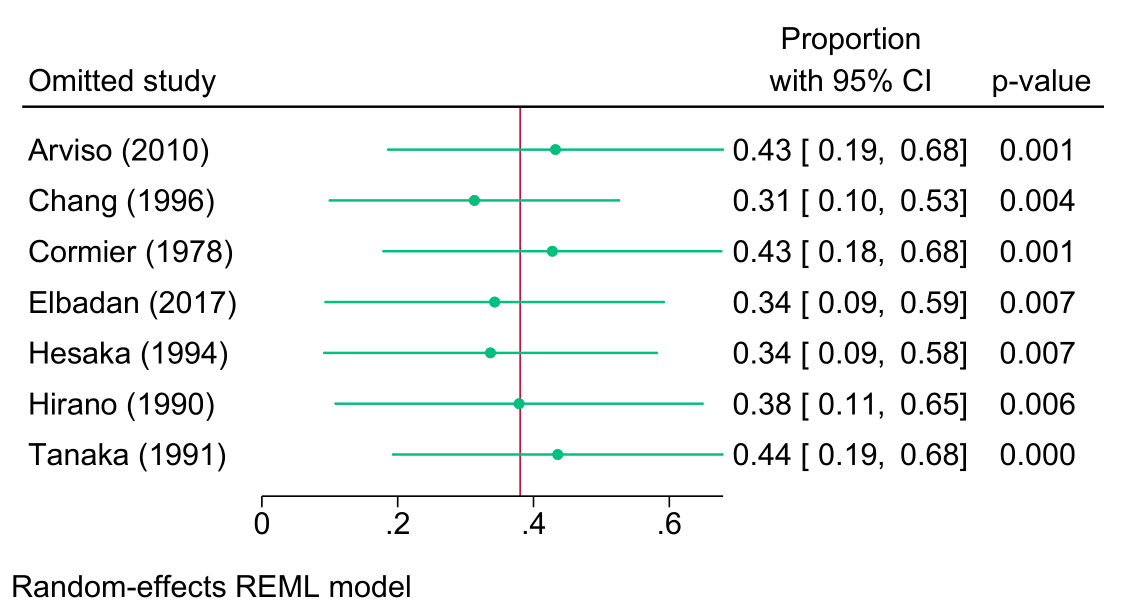


**SDC, Figure 29.** Leave-one-out sensitivity analysis of the prevalence of partial recovery of paralysis post-injection


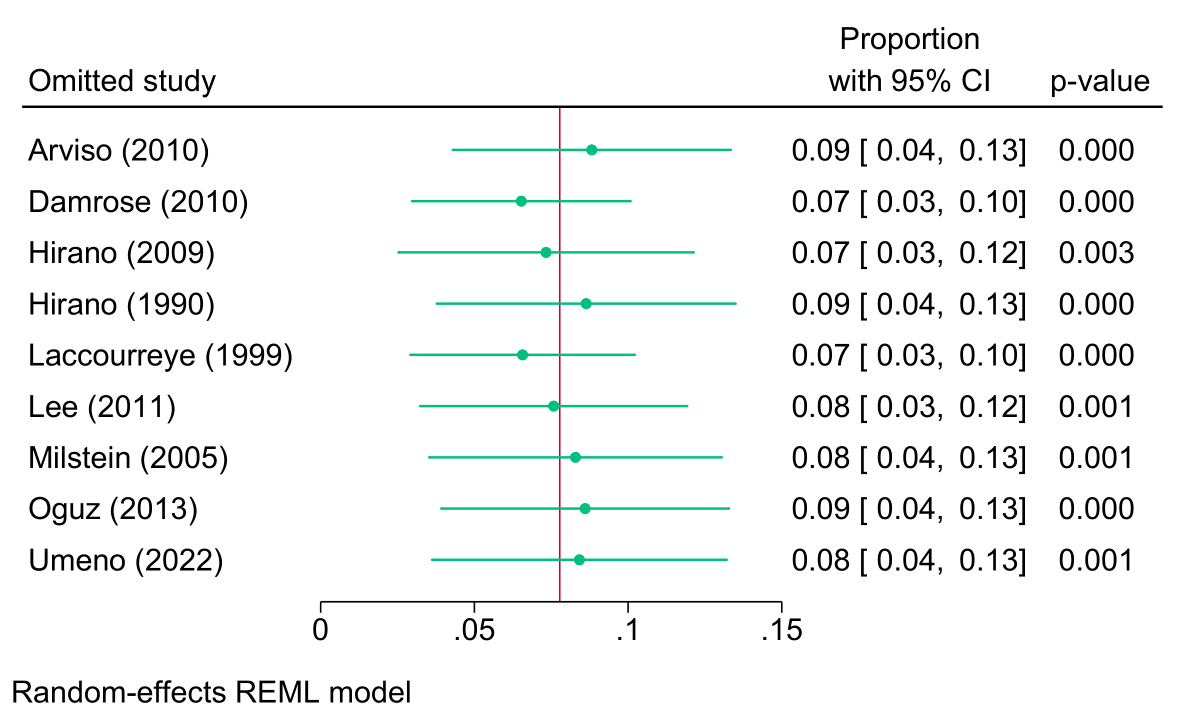


**SDC, Figure 30.** Leave-one-out sensitivity analysis of the prevalence of repeated injection of same material post-injection


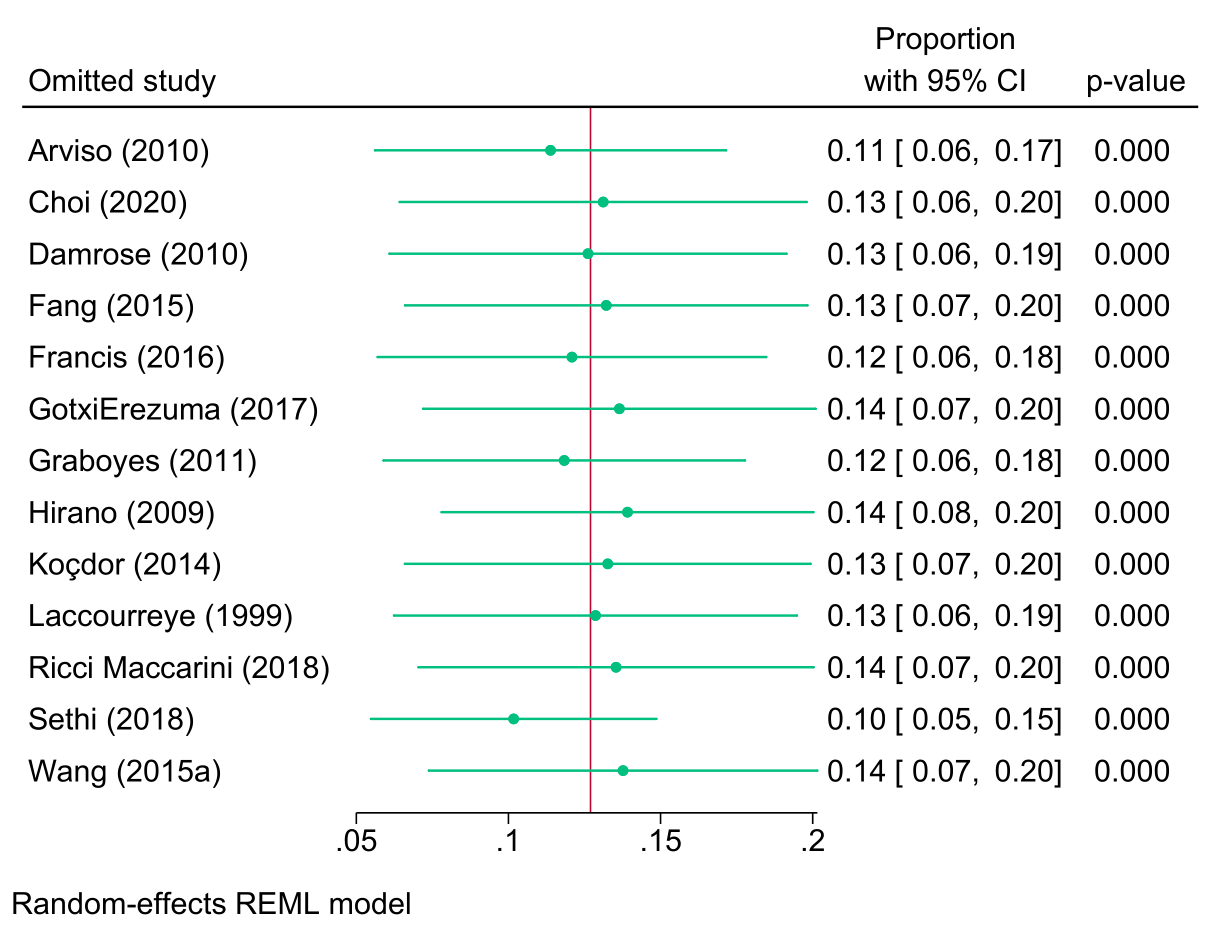


**SDC, Figure 31.** Leave-one-out sensitivity analysis of the prevalence of need for thyroplasty post-injection


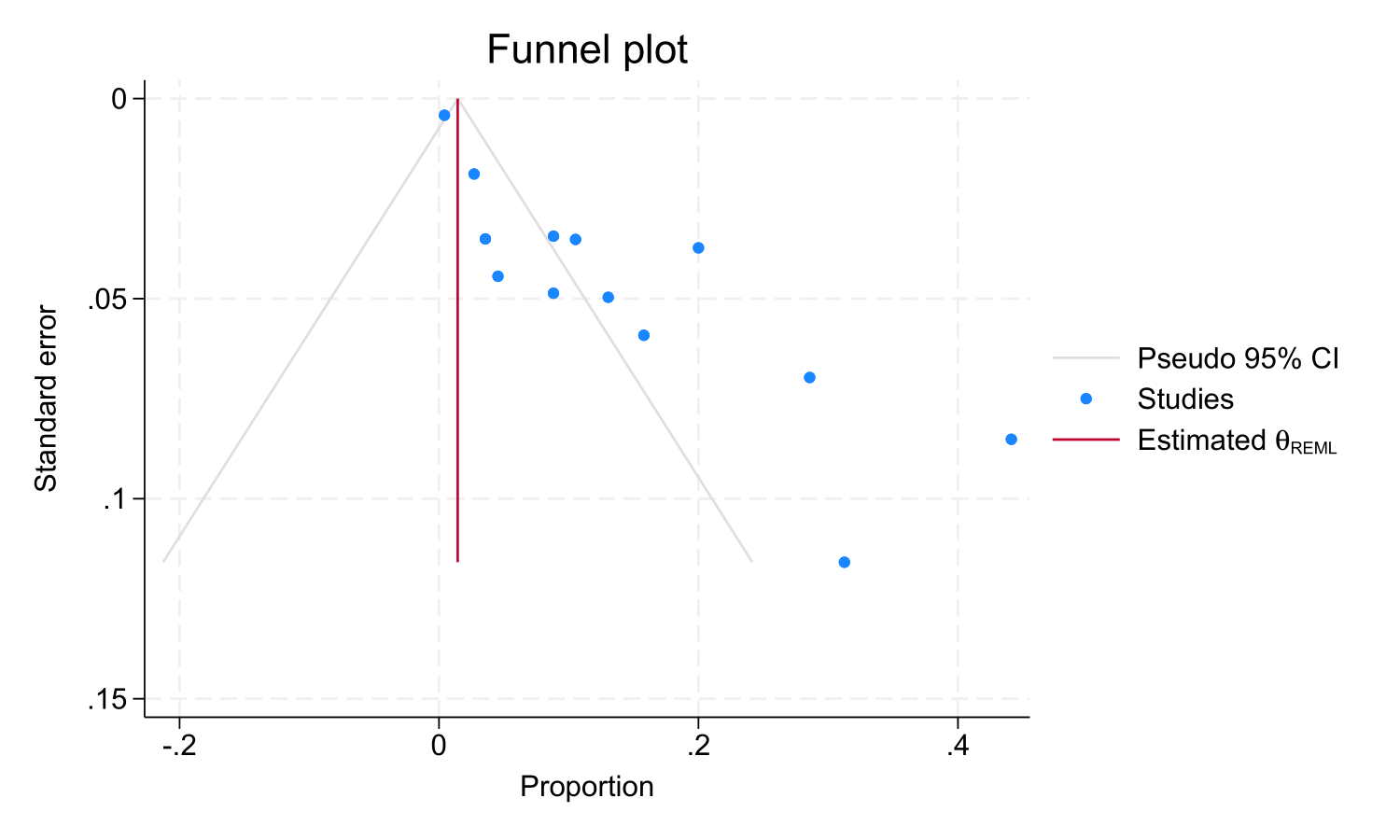


**SDC, Figure 32.** Funnel plot showing the risk of publication bias of the need of thyroplasty post-injection


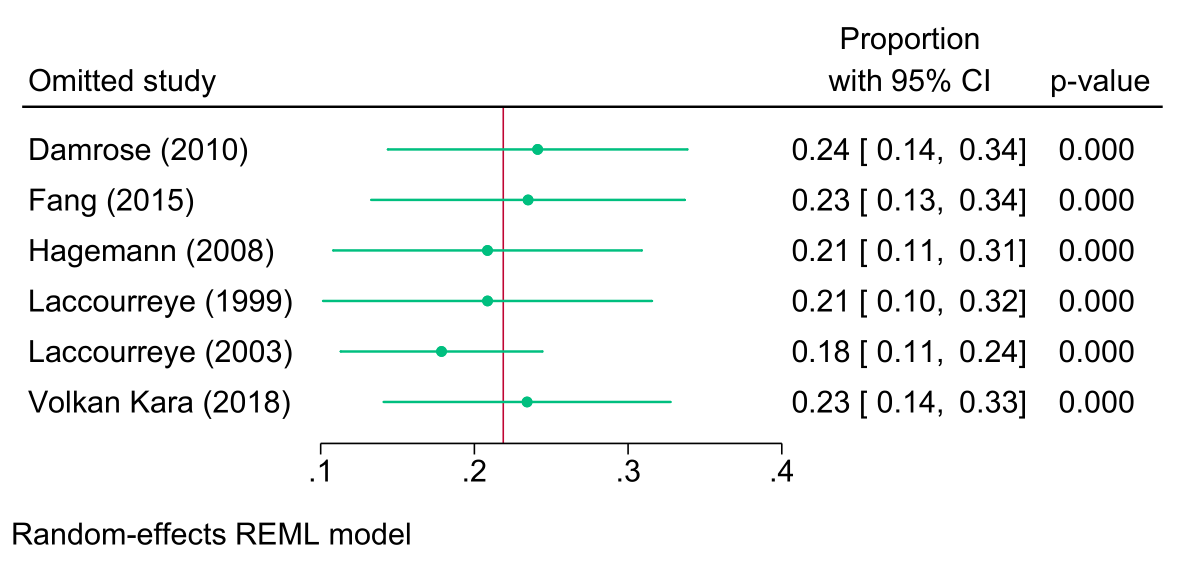


**SDC, Figure 33.** Leave-one-out sensitivity analysis of the prevalence of mortality post-injection
